# Quantification of Noncovalent Interactions in Azide–Pnictogen, –Chalcogen, and –Halogen Contacts

**DOI:** 10.1002/chem.202004525

**Published:** 2021-02-08

**Authors:** Markus Bursch, Lukas Kunze, Amol M. Vibhute, Andreas Hansen, Kana M. Sureshan, Peter G. Jones, Stefan Grimme, Daniel B. Werz

**Affiliations:** ^1^ Mulliken Center for Theoretical Chemistry, Institut für Physikalische und Theoretische Chemie Universität Bonn Beringstraße 4 53115 Bonn Germany; ^2^ Technische Universität Braunschweig Institut für Organische Chemie Hagenring 30 38106 Braunschweig Germany; ^3^ School of Chemistry IISER Thiruvananthapuram Kerala 695551 India; ^4^ Technische Universität Braunschweig Institut für Anorganische und Analytische Chemie Hagenring 30 38106 Braunschweig Germany

**Keywords:** azides, density functional calculations, local energy decomposition, London dispersion, noncovalent interactions

## Abstract

The noncovalent interactions between azides and oxygen‐containing moieties are investigated through a computational study based on experimental findings. The targeted synthesis of organic compounds with close intramolecular azide–oxygen contacts yielded six new representatives, for which X‐ray structures were determined. Two of those compounds were investigated with respect to their potential conformations in the gas phase and a possible significantly shorter azide–oxygen contact. Furthermore, a set of 44 high‐quality, gas‐phase computational model systems with intermolecular azide–pnictogen (N, P, As, Sb), –chalcogen (O, S, Se, Te), and –halogen (F, Cl, Br, I) contacts are compiled and investigated through semiempirical quantum mechanical methods, density functional approximations, and wave function theory. A local energy decomposition (LED) analysis is applied to study the nature of the noncovalent interaction. The special role of electrostatic and London dispersion interactions is discussed in detail. London dispersion is identified as a dominant factor of the azide–donor interaction with mean London dispersion energy‐interaction energy ratios of 1.3. Electrostatic contributions enhance the azide–donor coordination motif. The association energies range from −1.00 to −5.5 kcal mol^−1^.

## Graphical abstract

**The interactions that bind**: The noncovalent interactions between azides and oxygen‐containing moieties are investigated through a computational study based on experimental findings. A set of 44 high‐quality, gas‐phase computational model systems with intermolecular azide–pnictogen, –chalcogen, and –halogen contacts are compiled and investigated through semiempirical quantum mechanical methods, density functional approximations, and wave function theory.

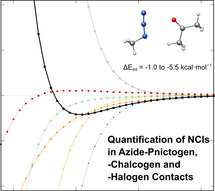

## Introduction

Noncovalent interactions play a very important role in biological, physical, and chemical sciences.[^1^, ^2^, ^3^, ^4^, ^5^] They are crucial for crystal packing, for the self‐assembly of large molecules in solution, and for biological pattern recognition, to name just a few examples.[^6^, ^7^, ^8^] In addition to highly electrostatic interactions (e.g., ion–ion, ion–dipole, dipole–dipole), the most prominent type is hydrogen bonding, but chalcogen–chalcogen[^9^, ^10^, ^11^, ^12^] and halogen–halogen interactions,[^13^, ^14^, ^15^] or combinations thereof, have also received significant attention during the last few decades. Furthermore, moieties that only consist of π*‐*systems often strongly interact with each other through so‐called π–π stacking interactions,[^16^, ^17^] and even between purely sp^3^‐hybridized hydrocarbon moieties one encounters weakly attractive forces that are mainly based on London dispersion.^[18]^

**Figure 1:**
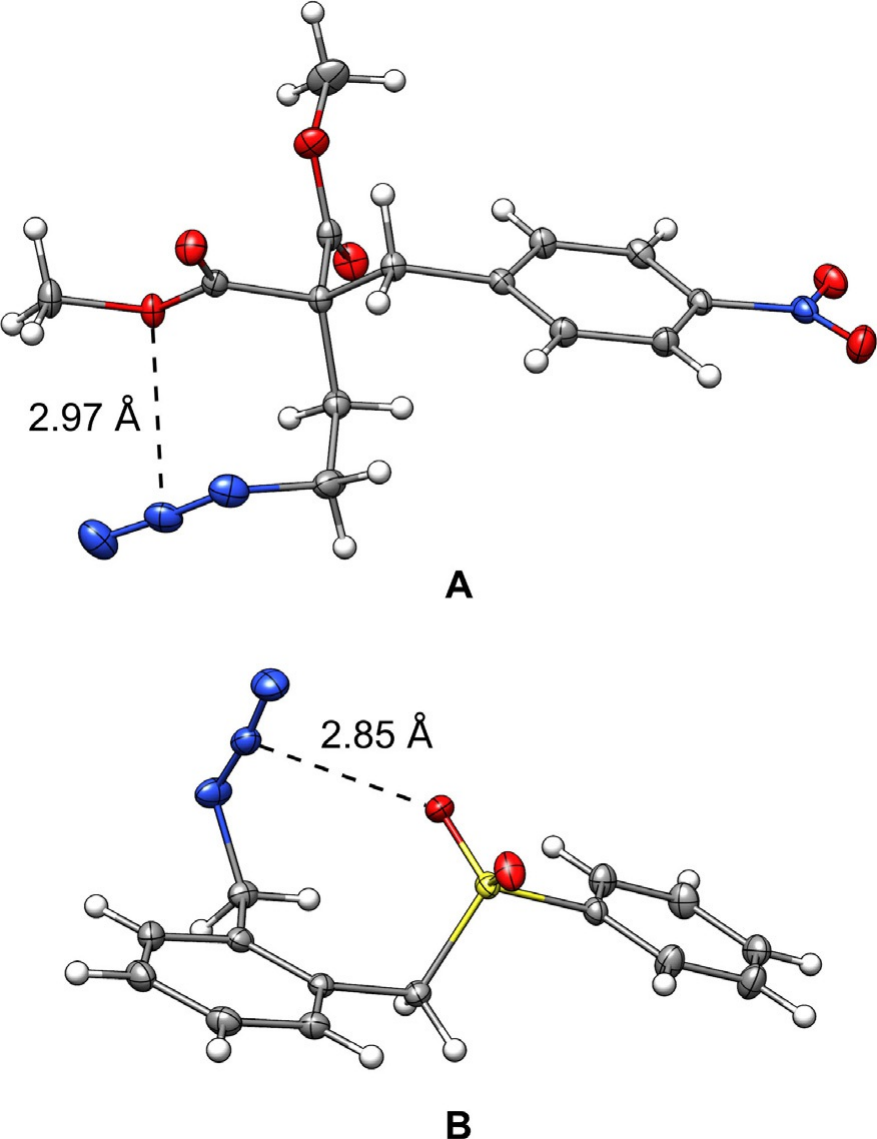
Two representative examples of compounds with close intramolecular oxygen–azide contacts in the solid. Nitrogen is depicted in blue, oxygen in red, sulfur in yellow, carbon in gray. The interatomic distances between N2 of the azide and the closest oxygen atom are given. Thermal ellipsoids are shown at the 50 % probability level.

**Figure 2:**
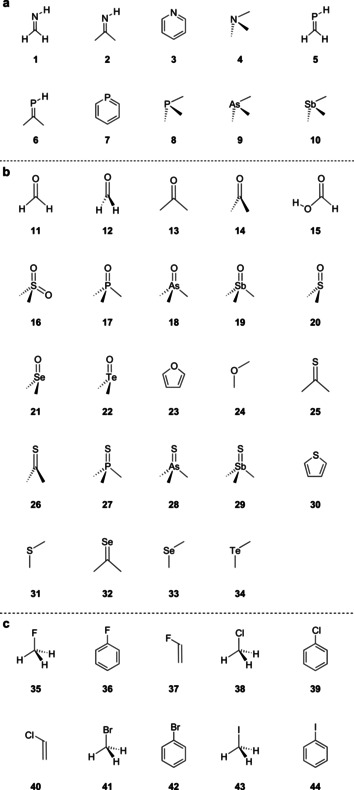
Overview of the investigated interacting model compounds and their orientations: a) pnictogens, b) chalcogens, and c) halogens.

**Figure 3:**
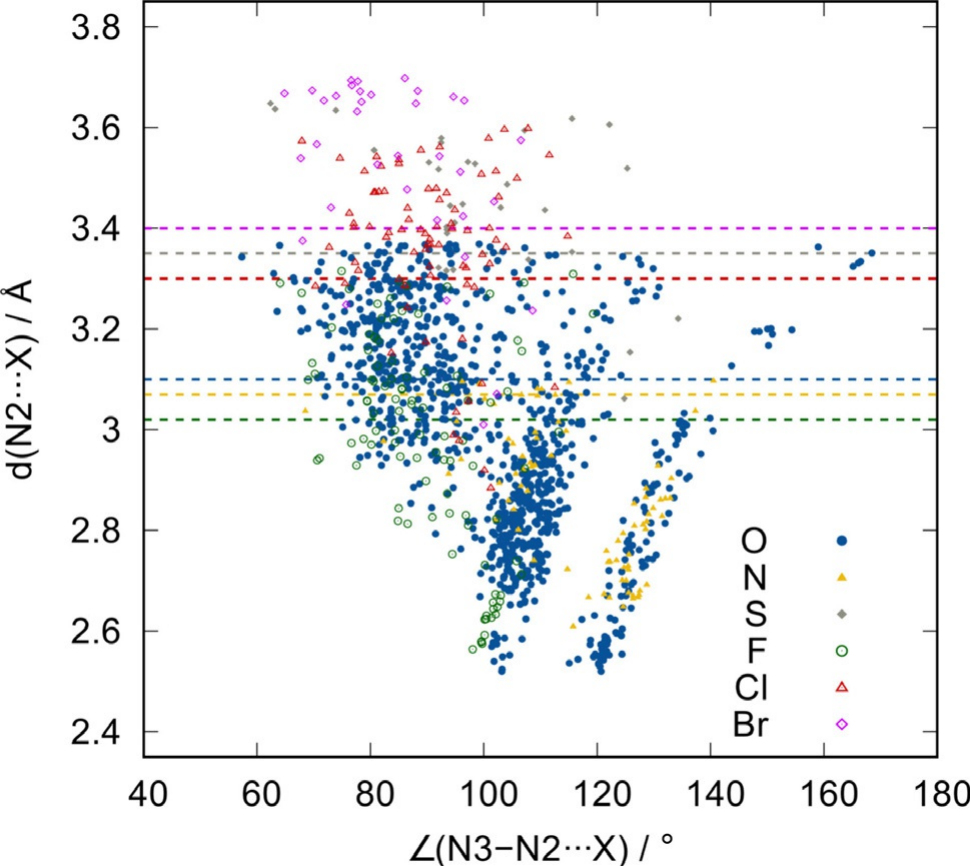
A plot of azide–oxygen, –nitrogen, –sulfur, –fluorine, –chlorine, and –bromine contacts of all structures deposited in the CCDC: N3−N2⋅⋅⋅X angle (in °) versus N2⋅⋅⋅X distance (in Å). The dotted lines show the corresponding sum of the van der Waals radii.

**Figure 4:**
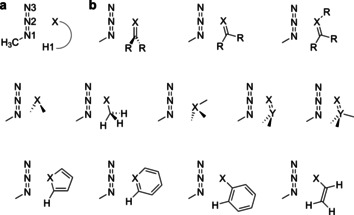
a) Numbering of relevant atom positions; b) schematic orientations of the interacting methylazide compound pairs. X represents the position of the directly interacting and Y of the indirectly interacting chalcogen, pnictogen, or halogen atom. R=H, Me.

All of these interactions have been successfully employed in crystal engineering and supramolecular assembly of compounds,[^19^, ^20^, ^21^, ^22^, ^23^, ^24^, ^25^, ^26^, ^27^, ^28^] and to facilitate catalysis.[^29^, ^30^, ^31^, ^32^, ^33^, ^34^, ^35^] During our solid‐state studies of some compounds containing flexible azide moieties and oxygen atoms, we noticed that mono‐ and divalent oxygen functionalities often displayed a close intramolecular contact with the central nitrogen atom (N2) of the azide moiety. In many of these cases, numerous other conformations would have been possible, but, nonetheless, the molecular conformation with the closest contact between N2 and O seems to be preferred. Two representative examples of molecules that show close azide–oxygen contacts in the solid state are depicted in Figure 1.^[36]^ In 2017, a close intermolecular contact between azide moieties and the oxygen of cucurbit[6]uril was discovered by Keinan and co‐workers, but not investigated in detail.^[37]^ To investigate whether these interactions led to a significant energy gain, we compiled a larger set of structures involving intermolecular contacts (Figure 2). Structures deposited in the Cambridge Crystallographic Data Centre (CCDC)^[38]^ were evaluated with respect to pnictogen–, chalcogen–, and halogen (henceforth denoted as PCH)–azide contacts. These contacts include, for example, divalent oxygen moieties, as in ethers or esters, or monovalent oxygen, as found in carbonyls, phosphine oxides, and sulfoxides. Figure 3 depicts data obtained for azide interacting moieties. It is observed that many of these contacts are much shorter than the sum of the van der Waals radii^[39]^ (3.07 Å for nitrogen⋅⋅⋅oxygen, blue dashed line in Figure 3). For the N3−N2⋅⋅⋅X angle (for a definition, see Figure 4), there is a strong accumulation of data points between 85 and 130°. Some of these might have to be interpreted with care because not every structure has been individually evaluated.

Furthermore, in some cases, the close contacts may have other causes than attractive oxygen–azide interactions. However, the strength and the nature of these interactions have not yet been investigated. Herein, we close this gap using quantum chemical computations, which indicate the preferred arrangements and respective interaction energies of PCHs interacting with azides.

## Results and Discussion

### Computational investigations

The intermolecular interactions between organic azide and PCH‐containing moieties in 44 model systems (10 pnictogen, 24 chalcogen, and 10 halogen; Figure 2) were investigated with respect to structural parameters and interaction energies. A schematic representation of the investigated orientations is depicted in Figure 4.

#### Structural properties

For all 44 azide adducts, the structural features of gas‐phase local minimum structures (SCS‐MP2[^40^, ^41^]/def2‐QZVPP^[42]^) were analyzed (Table 1). The model systems, which were chosen to resemble observed solid‐state interaction motifs, mostly show distances close to or only slightly below the sum of the van der Waals radii of the central nitrogen atom (N2; approx. 3.07 Å for O⋅⋅⋅N) and the interacting moiety. In the solid‐state structures, a large number of very short oxygen–nitrogen distances are observed. Nevertheless, the slight shortening, relative to the sum of van der Waals radii, hints at a relevant attractive interaction between the central nitrogen (N2) and the chalcogenide moiety. The predominant orientation of the PCH moiety at N3−N2⋅⋅⋅X angles of around 80–90° underlines the role of the central nitrogen as an interacting atom. For some cases involving a possible interaction with heavier elements, such as Sb or I, distortions of the N3−N2⋅⋅⋅X angles result from slight shifts of the X atom away from the azide moiety; this may result from the increased size and/or electronic properties of the corresponding moieties. The slight bending of the azide moiety (*θ*(N1‐N2‐N3)_mean_=174.1°) is almost unaffected by the interacting partner. The C‐N1‐N2‐X dihedral angle indicates the planarity of the coordination pattern. All investigated systems display an in‐plane orientation with the azide moiety.

**Table 1:** Structural parameters of azide adducts **1**–**44** optimized at the SCS‐MP2/def2‐QZVPP level of theory.

Adduct	X−N2 [Å]	X‐N2‐N3 [°]	X‐N1‐C [°]	*ϕ*(N1⋅⋅⋅H−X) [°]
**1**	3.125	90.6	173.1	120.7
**2**	3.170	82.8	179.2	157.4
**3**	3.052	89.3	174.3	119.4
**4**	3.071	87.3	175.1	114.6
**5**	3.501	98.7	161.1	120.3
**6**	3.562	89.6	168.9	162.8
**7**	3.498	97.6	162.4	123.2
**8**	3.546	100.5	159.7	125.8
**9**	3.581	102.4	157.2	125.2
**10**	3.706	110.2	147.7	122.2
**11**	3.068	89.8	174.0	117.3
**12**	3.104	86.4	175.0	72.8
**13**	3.118	81.6	179.2	154.8
**14**	3.033	86.9	176.1	104.8
**15**	3.082	87.8	175.6	176.0
**16**	3.045	84.7	178.8	142.1
**17**	3.008	84.3	179.6	135.0
**18**	2.973	84.1	178.6	132.9
**19**	2.923	87.2	179.1	124.5
**20**	3.038	81.7	178.0	146.6
**21**	2.981	81.0	176.6	149.5
**22**	2.963	80.9	176.1	150.6
**23**	2.961	88.5	174.6	115.8
**24**	2.878	86.9	172.6	118.2
**25**	3.594	81.1	177.1	167.6
**26**	3.451	80.4	177.5	114.2
**27**	3.531	82.7	177.6	142.6
**28**	3.523	82.7	177.9	139.7
**29**	3.456	85.6	175.5	129.3
**30**	3.337	98.4	161.4	113.0
**31**	3.309	95.2	165.4	126.8
**32**	3.669	81.8	176.3	169.1
**33**	3.382	98.5	161.2	125.7
**34**	3.474	101.8	157.1	124.0
**35**	3.020	87.6	175.7	126.1
**36**	3.119	80.7	177.8	150.5
**37**	3.169	79.5	177.3	149.1
**38**	3.439	88.9	171.7	139.0
**39**	3.443	80.5	178.2	160.5
**40**	3.471	79.8	178.5	157.2
**41**	3.559	89.2	170.4	141.9
**42**	3.538	81.2	176.9	162.5
**43**	3.724	90.6	168.2	144.2
**44**	3.678	82.7	174.5	164.5

#### Association and interaction energies

**Figure 5:**
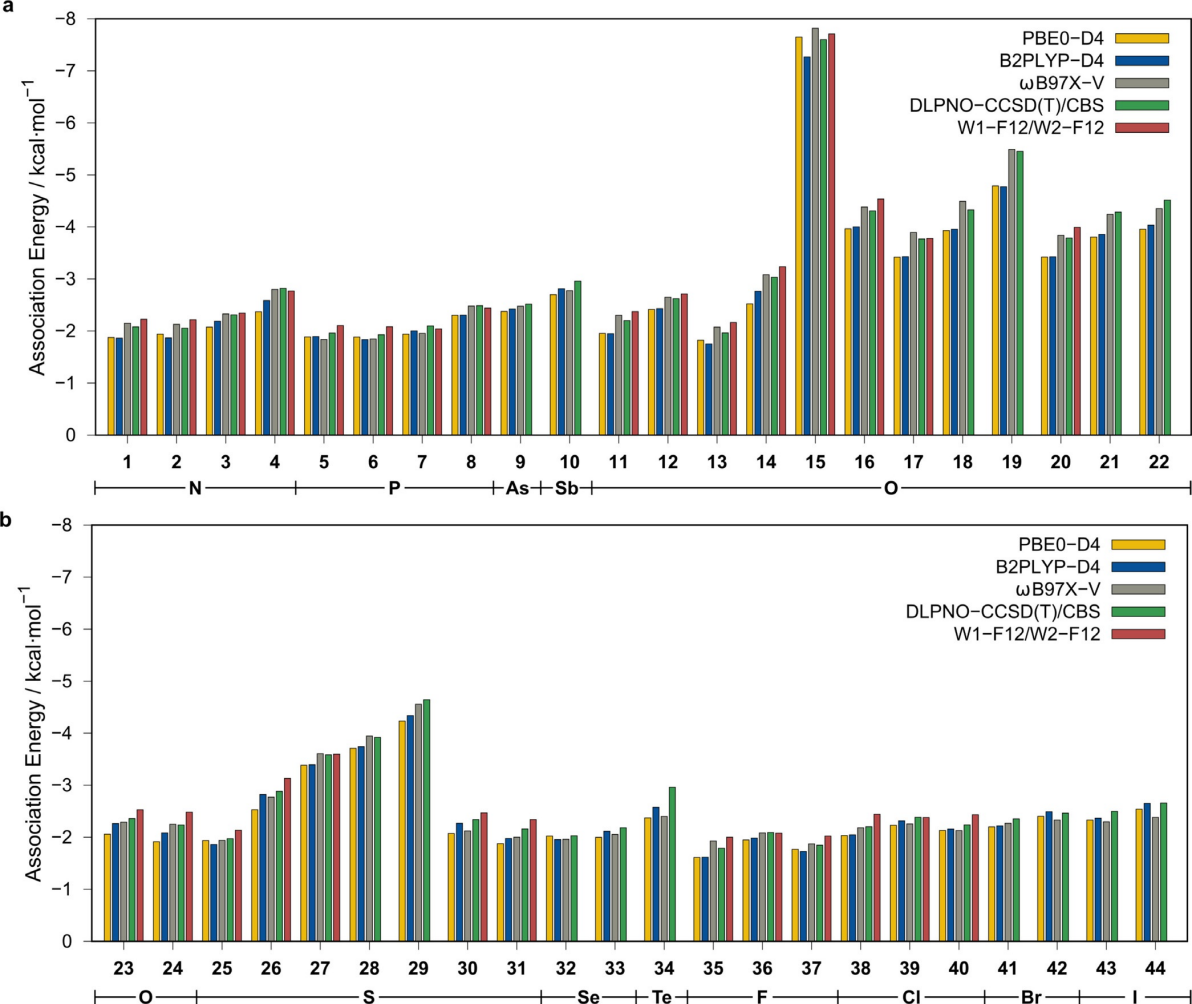
Gas‐phase association energies of pairs **1**–**44**. All DFT results were obtained with the def2‐QZVPP basis set.

**Figure 6:**
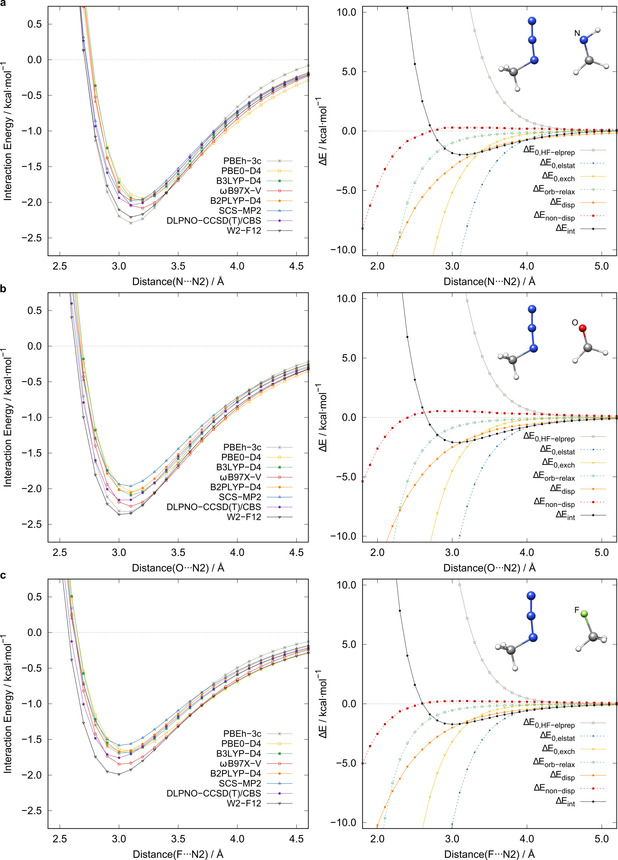
Interaction energy scans and local energy decomposition (LED) analyses (DLPNO‐CCSD(T)) for a) aldimine **1**, b) formaldehyde (**11**), and c) fluoromethane (**35**). All results were obtained with the def2‐QZVPP basis set, except for the composite methods.

The gas‐phase association energies for azide–donor pairs **1**–**44**, with respect to the relaxed dissociated monomer geometries (interaction energies are calculated with respect to the unrelaxed fragments), were calculated at the W2‐F12, W1‐F12,^[43]^ and DLPNO‐CCSD(T)[^44^, ^45^]/VeryTightPNO^[46]^ levels extrapolated to the complete basis set (CBS) limit (Figure 5). Deformation energies upon coordination are mainly small, on average, representing only <3 % of the association energies, with a maximum value of −9.3 % for phosphabenzene (**7**). For complexes including heavier elements, for which the W2‐F12 or W1‐F12 approaches are not applicable, DLPNO‐CCSD(T)/CBS association energies were calculated. This alternative approach proved satisfactorily accurate to reproduce the highly accurate association energies calculated at the W2‐F12/W1‐F12 level with very small statistical deviations (mean absolute deviation (MAD)=0.14 kcal mol^−1^) in agreement with recent benchmark studies.[^47^, ^48^] The calculated association energies range from −1.00 to −8.00 kcal mol^−1^ and several trends are observed. First, chalcogenide compounds seem to yield larger association energies compared with those of pnictogens and halogens. This is specifically the case for chalcogen atoms involved in highly polar bonds, as in pnictogen–chalcogenides. In systems **17**, **18**, and **19**, for example, the increasing electronegativity difference between the oxygen atom and central pnictogen upon descending Group 15 is well reflected by the increasing association energies, indicating the important role of electrostatic interactions. For less polar bound atoms, this trend is not as pronounced and, in some cases, even reversed, for example, for furan (**23**) and thiophene (**30**). Nevertheless, comparable electronegativity trends are observed for the pnictogens, although to a smaller extent (e.g., aldimine **1** compared with phosphaalkene **5**). If heavier fourth and fifth row elements are involved, the association energies become systematically larger, even though the electronegativity difference decreases or is even reversed. This indicates that, in addition to electrostatic interactions, dispersion interactions can play a dominant role in determining the association energies. Furthermore, the correlation of secondary pnictogen–pnictogen or pnictogen–chalcogen interactions with electronegativity can influence the observed trends. Overall, decomposition of the interaction energies indicates that a balance of electrostatic interactions and London dispersion determines the strength of the PCH–azide interaction. Sophisticated wave function theory (WFT)‐based methods are routinely applicable only to quite small systems (W2/W1‐F12: <30 atoms; DLPNO‐CCSD(T) with tight threshold settings and large basis set: <150 atoms) because of the high computational cost. Systems of more realistic size often include several hundreds or even thousands of atoms. Therefore, we assessed the reproduction of the association energy by common density functional approximations (DFAs) (applicable for <500 atoms) and tight‐binding‐based semiempirical quantum mechanical (SQM) methods of the GFNn‐xTB method family^[49]^ (applicable for <5000 atoms; Table 2). It was found that the range‐separated hybrid functional ωB97X‐V^[50]^ (MD=0.13, MAD=0.15 kcal mol^−1^) best reproduces the coupled‐cluster (CC) based association energies. Furthermore, all other tested D4‐corrected[^51^, ^52^] hybrid functionals perform reasonably well. The application of more costly double‐hybrid methods does not improve the results. Generally, a slight underestimation of the association energy, with respect to the CC reference values, is observed. Efficient small basis set composite DFT methods, such as B97‐3c^[53]^ and, specifically, PBEh‐3c,^[54]^ yield comparably good results to the methods applying a large quadruple‐*ζ* basis set. The SQM methods show a poorer performance, but both GFN1‐^[55]^ and GFN2‐xTB^[56]^ yield at least reasonable association energies. Furthermore, the capability of several DFT‐ and WFT‐based methods to reproduce the unrelaxed dissociation curve (W2‐F12//SCS‐MP2/def2‐ QZVPP level) of the azide–donor adduct was assessed (Figure 6). Almost all tested methods yield a satisfactory reproduction of the minimum distance, with a consistently very slight overestimation of the X⋅⋅⋅N2 distance. The depth and shape of the dissociation potential is generally reproduced well, with deviations below 0.5 kcal mol^−1^. Nevertheless, the error has to be seen in the context of the generally small interaction and association energies. Overall, the range‐separated hybrid functional ωB97X‐V reproduces the reference dissociation curves best. Surprisingly, the low‐cost small basis set composite method PBEh‐3c outperforms most of the other methods, with respect to the potential depth and position of the equilibrium distance, even though the interaction energy at increased distances is underestimated.

**Table 2:** Statistical measures for all tested DFAs, SQM methods, and force fields. All values are given in kcal mol^−1^.^[a]^

	Method	MD	MAD	SD	AMAX
WFT	SCS‐MP2	0.14	0.23	0.24	0.70
	DLPNO‐CCSD(T)	0.13	0.14	0.10	0.25
DFT	PBE‐D4	0.39	0.39	0.30	1.30
	TPSS‐D4	0.60	0.60	0.29	1.20
	B3LYP‐D4	0.26	0.26	0.15	0.54
	PBE0‐D4	0.32	0.32	0.18	0.71
	ωB97X‐V	0.13	0.15	0.14	0.56
	PWPB95‐D4	0.45	0.45	0.15	0.80
	B2PLY‐D4	0.28	0.28	0.16	0.68
composite	HF‐3c	−0.76	1.06	1.61	5.54
	B97‐3c	0.76	0.76	0.33	1.41
	PBEh‐3c	−0.20	0.29	0.30	0.85
SQM	GFN1‐xTB	0.90	1.23	1.16	4.59
	GFN2‐xTB	1.08	1.32	1.06	3.28
	PM6‐D3H4X	3.85	3.96	11.24	49.11
	PM7	2.51	3.10	7.42	32.24

[a] MD=mean deviation; SD=standard deviation; AMAX=maximum absolute deviation. If no W2‐F12/W1‐F12 reference values were available, DLPNO‐CCSD(T) values were used as a reference. The DLPNO‐CCSD(T) values are evaluated with reference to W2‐F12/W1‐F12 values, if available.

#### Interaction energy decomposition analysis

Generally, various components can be postulated for the interactions with the azide, principally 1) donor–acceptor interactions (orbital relaxation); 2) electrostatic interactions; 3) electron correlation, including specifically long‐range correlation effects, such as London dispersion, and 4) Pauli exchange repulsion. Energy decomposition schemes can help to quantify these components, and thus, help to understand the nature of the interactions. Specifically, canonical energy decomposition analysis (EDA),^[57]^ symmetry‐adapted perturbation theory (SAPT),^[58]^ and the DLPNO‐CCSD(T)‐based local energy decomposition (LED)[^59^, ^60^] have proved to yield reasonable insights.^[61]^ Therefore, we applied LED to all complex equilibrium geometries and to three representative model systems, **1**, **11**, and **35**, as a function of the X⋅⋅⋅N2 distance (Figure 6). The LED scheme decomposes the interaction energy into contributions including the electronic preparation energy at the Hartree–Fock (HF) level (*E*_HF‐elprep_), which represents the repulsive part of the exchange interaction and can be conceptionally referred to as “Pauli repulsion”. Other major components are the attractive exchange energy, *E*_exch_; electrostatic interactions, *E*_elstat_; and London dispersion interactions, *E*_disp_, henceforth named dispersion for simplicity. Minor contributions are represented by the perturbative triple correction to the interaction energy, *E* ^(T)^, and *E*_non‐disp_, which mainly represents a correction to errors in the permanent electrostatic interactions originating from HF overestimation of dipole moments. Another decomposition into unrelaxed (“frozen state”) and relaxed (“SCF state”) energy contributions is also possible, allowing the quantification of an orbital relaxation energy contribution, *E*_orb‐relax_, which may be, to some extent, comparable to the orbital relaxation term of Morokuma‐type EDA schemes and includes charge‐transfer (CT), polarization, and induction effects.^[62]^ The frozen‐state energy contributions are indicated by the suffix “0”. The total decomposition of the interaction energy is given by Equation 1:

(1) $$E{}_{\mathrm{tot}}=E{}_{0,\mathrm{HF}\text{-}\mathrm{elprep}}+E{}_{0,\mathrm{elstat}}+E{}_{0,\mathrm{exch}}+E{}_{\mathrm{orb}\text{-}\mathrm{relax}}+E{}_{\mathrm{disp}}+E{}_{\mathrm{non}\text{-}\mathrm{disp}}$$

The total dispersion energy term, *E*_disp_, is calculated by summing the CCSD dispersion energy part; the weak‐pair contributions; and the scaled intermolecular triple correction, *E* ^(T)^, given by Equation 2. The correction factor, *γ*, is the ratio of the strong‐pair dispersion contribution and the total intermolecular strong‐pair contribution.

(2) $$E_{\mathrm{disp}}=E_{\mathrm{disp}}^{C-\mathrm{SP}}+E^{C-\mathrm{WP}}+\gamma E^{\left(T\right)}$$

**Figure 7:**
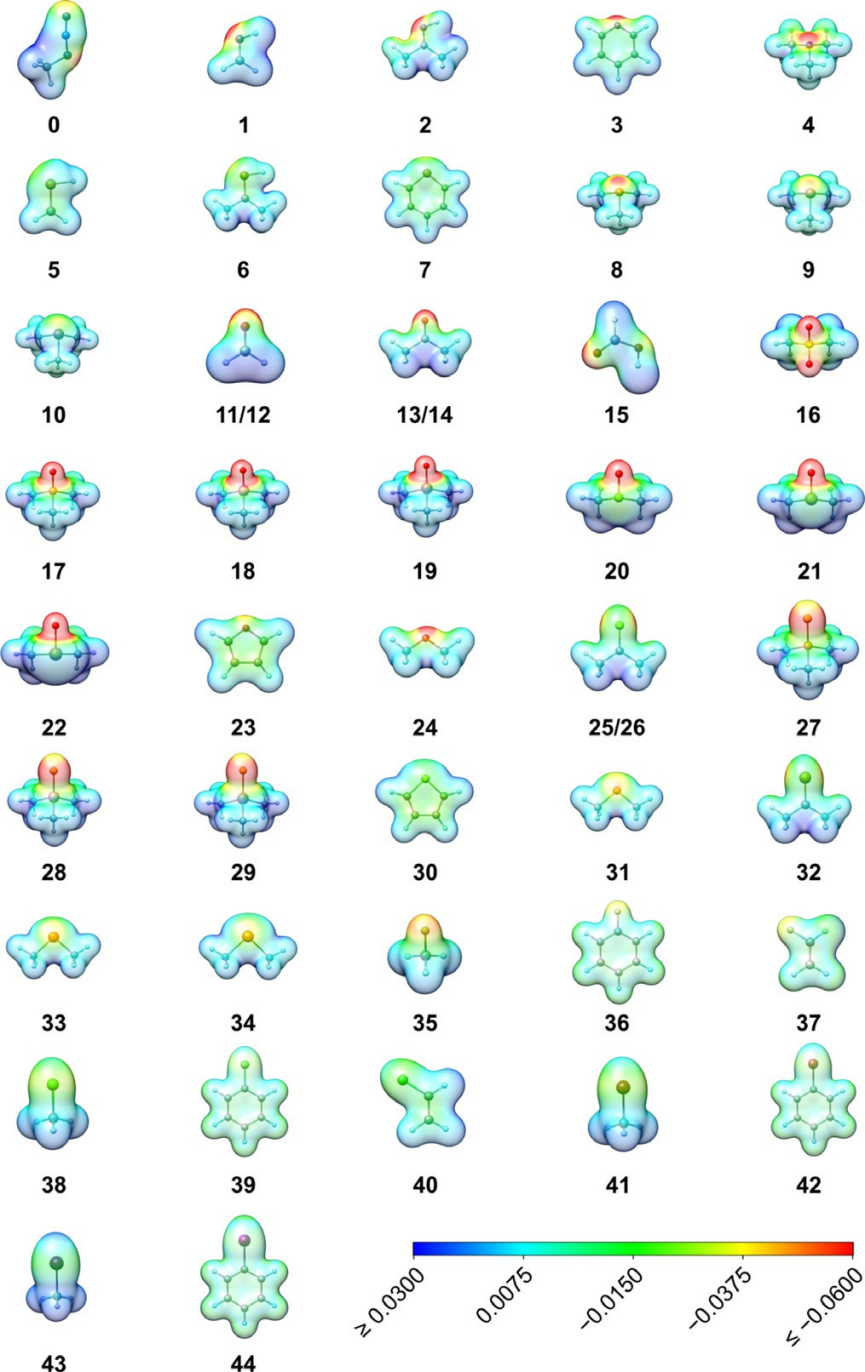
Molecular ESP (in a.u.) plots at the PBE0/def2‐SVP//SCS‐MP2/def2‐QZVPP level with respect to a positive probe charge. Red indicates attractive regions and blue indicates repulsive regions.

The third term of Equation (2) is an estimate of the perturbative triple contribution to the intermolecular dispersion energy. The remaining part of the triple contribution is incorporated in the *E*_non‐disp_ contribution (for further details of the energy decomposition, see the Supporting Information). For all investigated systems, the LED scheme identifies exchange, London dispersion, and electrostatic interactions as the dominant attractive components of the interaction energy, whereas orbital relaxation effects seem to play a minor role. In the following, we investigate the role of these three attractive contributions in some detail. On inspecting the molecular electrostatic potentials (ESPs; Figure 7), a clear correlation of the structural features and the electrostatic properties of the interacting atoms can be recognized. The ESPs show the expected electron‐poor region at the central nitrogen atom (N2) of the azide moiety. Thus, the electrostatic interactions of electron‐rich regions of the interacting molecule should be improved with increased electron density difference between N2 and X. This is in line with the observed structural properties of an angular fixation and shortened distances in the case of strong differences in the ESP regions of the interacting moieties.

#### Hydrogen‐bonding and orbital interactions

In some structures, secondary hydrogen bonds (HBs) contribute to the overall interaction energy. To qualitatively estimate this contribution, a natural bond orbital^[63]^ (NBO) based approach was applied, as recommended by Weinhold and Glendening,^[64]^ at the PBE0‐D4/def2‐QZVPP level of theory. In this context, a second‐order perturbation theory (SOPT) estimate of the stabilization energy ($\sum E_{n\to \sigma {}^{*},\mathrm{HB}}^{\left(2\right)}$ ), resulting from CT from the lone pair (LP), n, at N1 of the azide moiety into the antibonding orbital, $\sigma_{X-H}{}^{*}$ , of the HB donor, is applied.[^65^, ^66^] CT is mostly proposed as the dominant attractive energy contribution in hydrogen bonding.^[67]^ Nevertheless, this attractive component has to be offset against the steric repulsion component ($\sum E_{\sigma \to n,\mathrm{HB}}^{\mathrm{rep}}$ ), involving the corresponding bonding, $\sigma_{X-H}$ , orbital, and is estimated from NBO analysis.[^68^, ^69^] The resulting hydrogen‐bonding strength estimate ($E_{\mathrm{HB}}^{\mathrm{NBO}}$ ) is calculated from Equation 3.

(3) $$E_{\mathrm{HB}}^{\mathrm{NBO}}=\sum E_{n\to \sigma {}^{*},\mathrm{HB}}^{\left(2\right)}+\sum E_{\sigma \to n,\mathrm{HB}}^{\mathrm{rep}}$$

**Figure 8:**
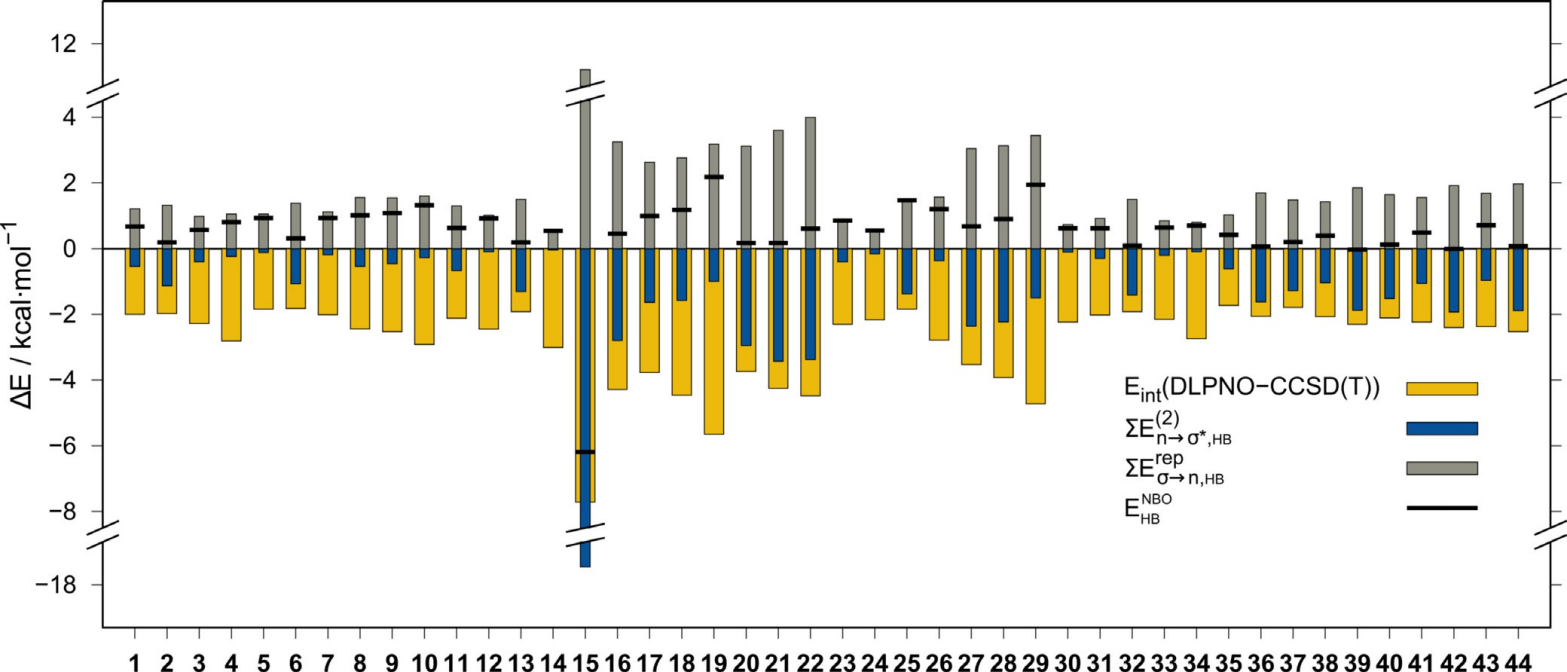
NBO‐based HB strength estimates for **1**–**44** with respect to LED interaction energies.

**Figure 9:**
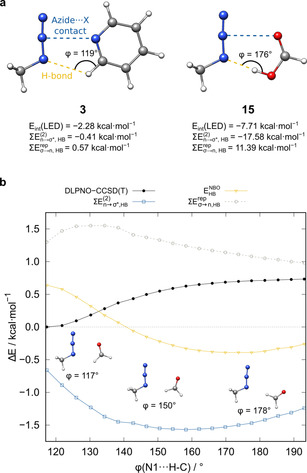
a) Relative LED interaction energies, CT stabilization energies, and steric repulsion energy estimates for hydrogen bonding in **3** and **15**. b) Interaction energy, CT, and steric repulsion estimates as a function of angle *ϕ*(N1⋅⋅⋅H−X) for **11**.

Notably, further much smaller, attractive components, such as the London dispersion, are not included in this estimate; thus, for very weak hydrogen‐bonding contacts, repulsive estimates are expected. This is mostly the case if the corresponding N1⋅⋅⋅H−X angle deviates strongly from the optimum 180° region,^[70]^ which is the case for most of the presented model systems (cf. Figures 8 and 9 a, *ϕ*(N1⋅⋅⋅H‐X)_mean_=136°, Table 1). The angle dependence of the HB strength estimates for **11** is depicted in Figure 9 b. A clear relationship between CT and steric repulsion estimates with N1⋅⋅⋅H−X is observed. Furthermore, the attractive nature of the oxygen⋅⋅⋅azide contact is verified by the total interaction energy increase with larger *ϕ*, even though hydrogen bonding is enhanced and approaching linearity. Except for the strongly hydrogen‐bonded formic acid adduct (*E*_NBO_=−6.19 kcal mol^−1^), NBO analysis yields mainly slightly repulsive HB estimates for almost all other model systems (Figure 8). Overall, this reveals only a minor role of hydrogen bonding in the investigated model systems, which is in agreement with the observed small angles, *ϕ*, and the comparably large N1⋅⋅⋅H distances. Nevertheless, due to other weakly attractive components, such as the London dispersion, the secondarily hydrogen‐bonded adducts are slightly preferred on the potential‐energy surface (PES) in the intermolecular case. Thus, to investigate true local minima on the PES, this secondary effect was accepted for the model systems.

**Figure 10:**
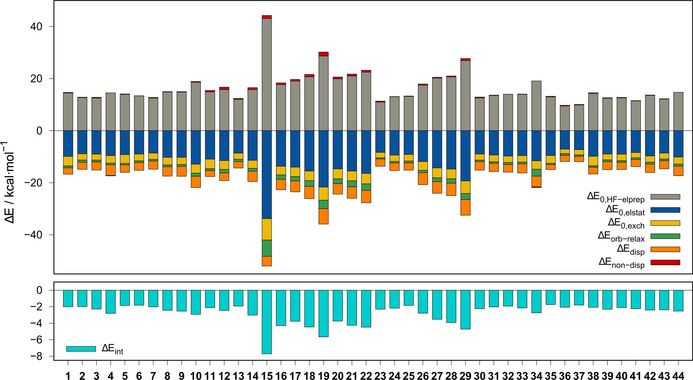
LED contributions and interaction energies for **1**–**44** at the DLPNO‐CCSD(T)/def2‐QZVPP level.

**Figure 11:**
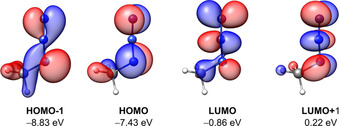
Selected Kohn–Sham molecular orbitals at the PBE0/def2‐QZVPP//SCS‐MP2/def2‐QZVPP level of theory for MeN_3_. Isosurface value=0.05 e^−1/2^ bohr^−3/2^.

Deviations from local minimum structures may result in artificially less attractive or even slightly repulsive adduct structures, even though the general nature of the azide–X interaction is clearly attractive. Therefore, a perfect transfer of the crystal‐structure motifs into the gas phase is not possible. Compared with the chalcogen–azide interaction, other interactions, such as chalcogen–chalcogen, have a significant Lewis‐like donor–acceptor bonding character, and thus, a greater influence of orbital relaxation effects is observed. For the azide–donor interaction, this is not generally the case. The LED analysis consistently yields minor orbital relaxation contributions for all 44 systems (Figure 10). This is attributable to the nature of the frontier molecular orbitals involved (Figure 11). The LUMO of the azide moiety is delocalized over the whole azide function and the orbital contribution at the N2 atom competes with the occupied HOMO‐1, which represents the LP at N1, which is generally involved in secondary hydrogen bonding. Thus, the overall donor–acceptor interaction cannot benefit from X→N2 donation. This picture may change upon orientation changes to some interaction partners with double‐bond moieties. For the latter, if the orientation changes from an in‐plane to an orthogonal structure, the azide can become an electron‐donating moiety and a small n→π✶ contribution is observed (cf. systems **12**, **14**, and **26**). Comparable behavior is observed for systems that allow for n→σ✶(X−C/H) donation, such as **5**. Here, a small CT contribution of −0.38 kcal mol^−1^ for n→σ✶(P−H) is observed. These secondary interactions (pnictogen–pnictogen and pnictogen–chalcogen, see Table S13 in the Supporting Information) explain some comparably large interaction energies observed for systems such as **10**, which involves an n→σ✶(Sb−C) CT contribution of −0.72 kcal mol^−1^, or **19**, with a contribution of −1.58 kcal mol^−1^. These observations are qualitatively in line with larger orbital relaxation terms in the LED analyses for the corresponding systems. Considering these contributions, the corresponding systems end up in a comparable interaction energy range to that of systems lacking such secondary interactions.

#### London dispersion interactions

**Figure 12:**
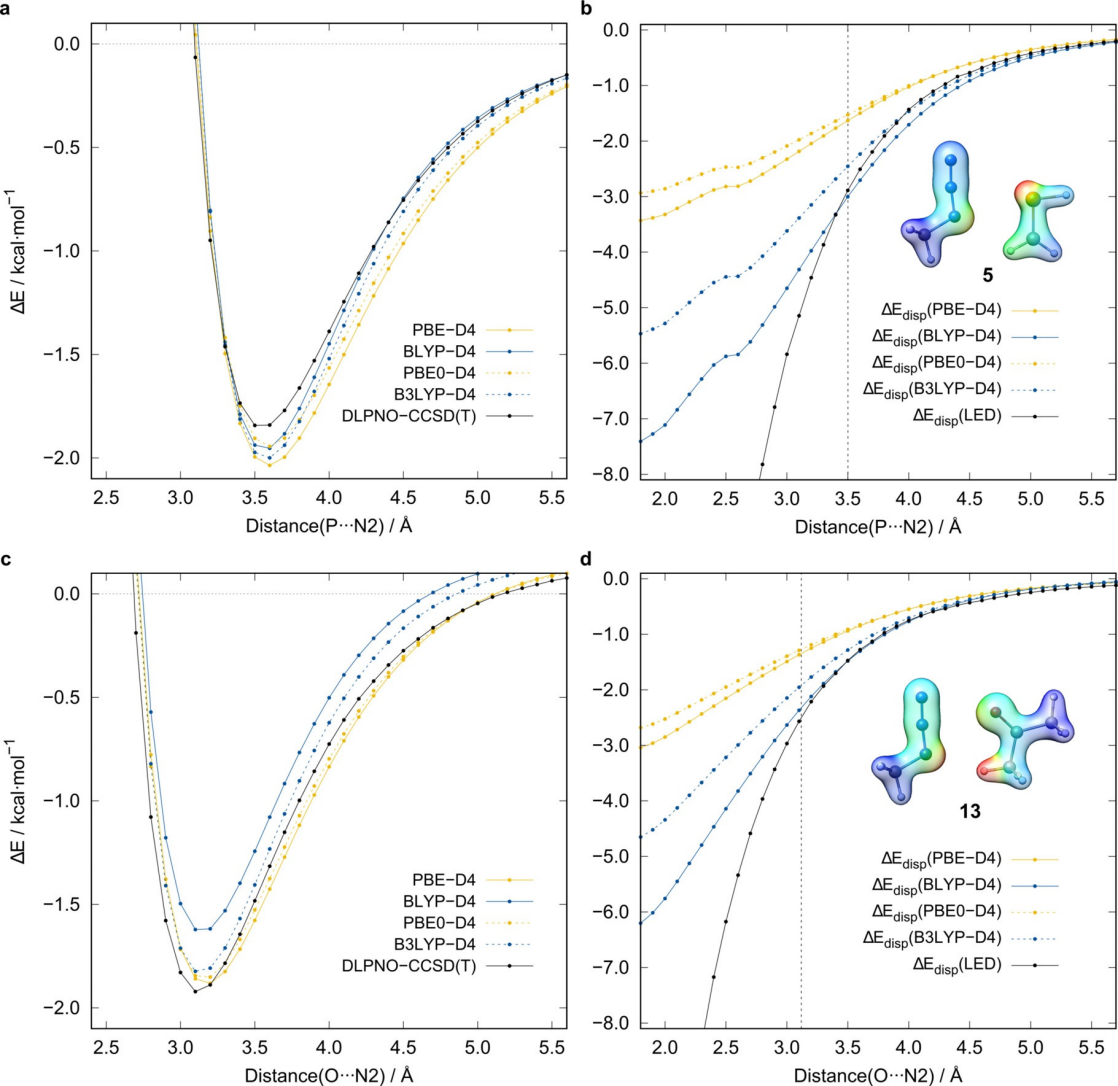
a) Interaction energy and b) London dispersion interaction estimates as functions of the P⋅⋅⋅N2 distance with the dispersion interaction density (DID) plot for **5**. c) Interaction energy and d) London dispersion interaction estimates as functions of the O⋅⋅⋅N2 distance with a DID plot for **13**. Isosurface value=0.1 e bohr^−3^; blue indicates low and red indicates high London dispersion interactions. The vertical dashed lines indicate the respective X⋅⋅⋅N2 distance.

**Figure 13:**
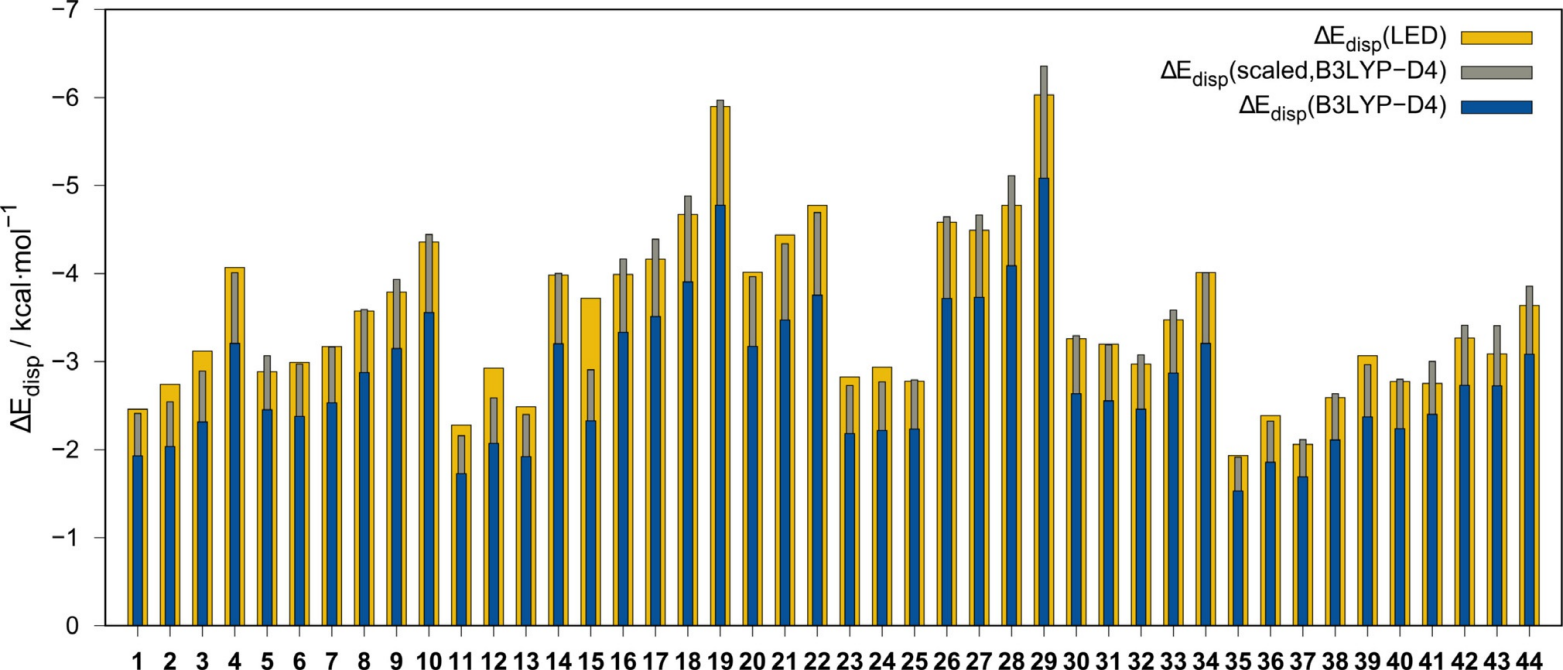
A comparison of the LED London dispersion contribution and the D4 correction to B3LYP for the interaction energy. Scaling factor applied for Δ*E*_disp_(scaled,B3LYP‐D4) is 1.25.

Another important attractive component of the interaction energy is the London dispersion. The extent of its influence, as estimated from LED analysis, is shown in Figures 10 and 12. Prior studies indicated that the DFT‐D4 dispersion correction for a somewhat repulsive DFA, such as B3LYP (with almost no indirect, intrinsic reproduction of London dispersion effects), correlated well with the LED dispersion energy and could be applied for a low‐cost estimate of the dispersion interaction energy.^[71]^ To verify this estimate, we analyzed the correlation of the B3LYP‐D4[^72^, ^73^] correction and the LED dispersion interaction energy for all 44 systems (Figure 13). On average, 80 % of the LED dispersion energy is covered by the D4 correction to B3LYP; only for **15** is this value underestimated at 63 %. A scaling of the B3LYP‐D4 correction by a factor of 1.25 yields 100 % reproduction, on average, for the investigated systems. Nevertheless, this estimate should be used with caution because the quality may vary for more exotic systems. The Δ*E*_disp_/Δ*E*_tot_ ratio can be further utilized to identify whether the interaction is dominated by the London dispersion. A value of >1 indicates an interaction dominated by the London dispersion (cf. 1.92 for the methane dimer^[59]^). For all systems, except **15**, this ratio is close to or above 1.00, with a maximum of 1.65 for **26** and a mean value of 1.30 (for individual data, see the Supporting Information). Furthermore, the N2⋅⋅⋅X distance dependence of the LED dispersion energy and the D4 dispersion correction for four representative DFAs (PBE,^[74]^ PBE0,^[75]^ BLYP,[^76^, ^77^, ^78^] B3LYP) was investigated (Figure 12) for **5** and **13**, as examples. Both repulsive DFA (BLYP, B3LYP) D4 corrections correlate very well with the LED dispersion energy in the dissociative regions. Specifically, at the equilibrium distance, the corrections match the LED estimate well. For less repulsive functionals, such as PBE and PBE0, the D4 corrections are much smaller. Because B3LYP reproduces the association and interaction energies much better than those with BLYP, the former is recommended for an estimate of the dispersion interaction energy.

#### Conformational studies

**Figure 14:**
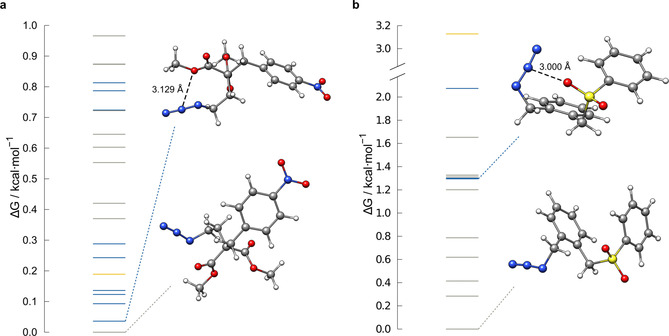
Relative gas‐phase conformational free energies for a) **A** and b) **B**. The position of the optimized molecular X‐ray structure is highlighted in yellow. All conformers containing an azide–oxygen contact are highlighted in blue, all without are in gray. All free energies were calculated at the DLPNO‐CCSD(T)/CBS//SCS‐MP2/def2‐TZVPP level for *T*=25 °C.

**Figure 15:**
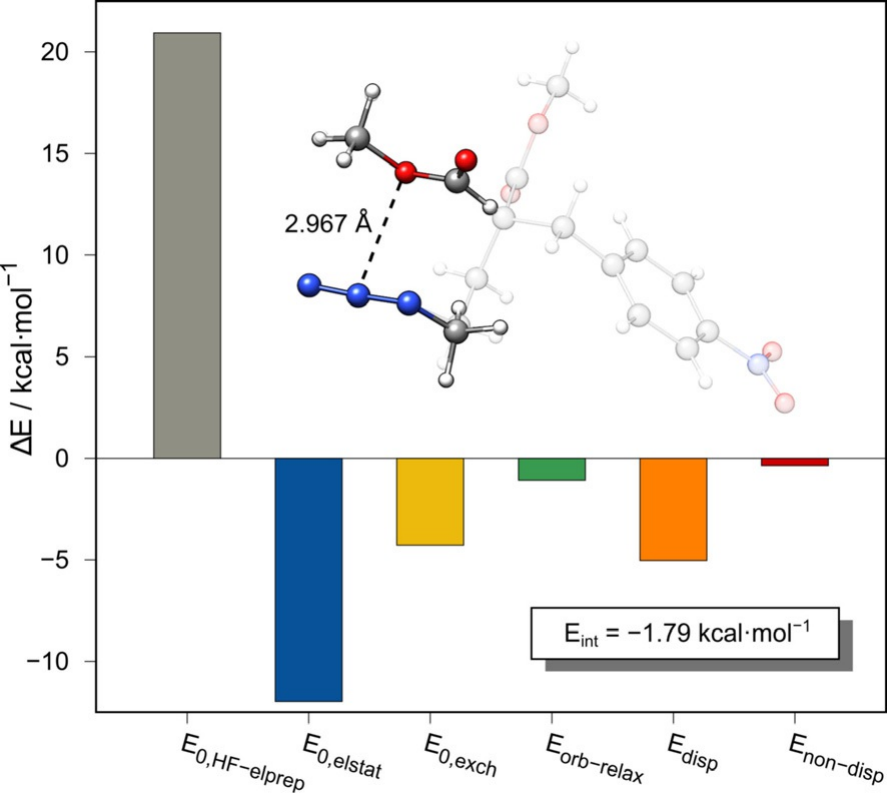
LED analysis of an intermolecular model system of **A** at the DLPNO‐CCSD(T)/def2‐QZVPP//SCS‐MP2/def2‐QZVPP level. The corresponding X‐ray structure is depicted transparently.

To estimate the transferability of the observed structural motif preference from the solid to gas phase, example conformational studies were conducted by starting from the molecular solid‐state structures of **A** and **B** (Figure 1). For both structures, a conformer ensemble was generated fully automatically by applying the CREST[^79^, ^80^] program at the GFN2‐xTB level. The obtained conformers were reranked according to Gibbs free energy at the DLPNO‐CCSD(T)/CBS//SCS‐MP2/def2‐TZVPP level and analyzed for the presence of an azide–donor contact (Figure 14). Although for both compounds, according to the Gibbs free energy, more favorable gas‐phase conformers were found, only for **A** is the low free energy conformer region dominated by structures with short azide–donor contacts. Here, the lowest conformer shows proximity of the donor moiety to the azide, but, because the distance is long, it was classified as a nonmotif structure. For **B**, the lowest conformers do not show pronounced azide–donor contacts. Here, the first conformer with this motif is approximately 1.3 kcal mol^−1^ higher in free energy than that of the lowest. Thus, we conclude that the structural features only partly originate from azide–donor interactions present in the gas phase, indicating a possible role of solid‐state effects, such as crystal packing. Crystal effects may be large enough to favor an azide–donor arrangement, even though the attractive interaction may not be large enough to fix this structural motif in the gas phase or in solution. Furthermore, structural strain and steric repulsion effects are influenced by the bridging moiety between the azide and donor. LED analysis of a simplified intermolecular model of **A** optimized with a constraint on the O⋅⋅⋅N2 distance and essential dihedral angles reveals an interaction energy of −1.79 kcal mol^−1^, which is on the same scale as that of comparable model systems (e.g., **24**; Figure 15).

## Conclusion

The structural motif of an azide moiety in close contact with an electron‐rich donor moiety is observed in surprisingly many solid‐state structures. To understand the interaction between the azide and the partner molecule or moiety, we created 44 intermolecular model systems to investigate computationally the nature of the interaction in the gas phase. Furthermore, several new organic compounds were synthesized, with the aim of systematically creating a structural motif that was found empirically in the CCDC. Overall, the calculated (W2‐F12/W1‐F12/DLPNO‐CCSD(T)/CBS) association energies for side‐on azide–X complexes varied between −1.00 and −5.50 kcal mol^−1^ (formic acid (**15**) excluded). The LED analysis of the interaction identifies electrostatic and London dispersion interactions as the dominating attractive contributions to the interaction energy. If the electrostatic contribution is large enough to overcome less favorable steric motifs, and secondary hydrogen bonding is not dominant, the X⋅⋅⋅N2 interaction motif is preserved. In this motif, little or no Lewis‐like donor–acceptor bonding is observed, which is reflected by the comparably small orbital relaxation contributions to the interaction energies. This flexibility of the interaction components further explains the large variety of structural patterns found in many solid‐state structures. A comparison of D4 London dispersion corrections to the LED results indicates good comparability of both energy contributions for the investigated systems, in accordance with previous studies. Conformational studies on the newly synthesized compounds indicate that other intermolecular crystal (packing) effects may play an important role in stabilizing this weak azide interaction. Overall, considering the complexity of further decomposing single contributions to the interaction energy into specific parts of the molecular systems, the unbiased azide–X interaction is estimated to be 1.5 to 3.0 kcal mol^−1^. We are convinced that these stabilizing interactions are of importance, not only for the arrangement of azide moieties in supramolecular assemblies and crystal engineering (e.g., click chemistry in the solid state),[^81^, ^82^, ^83^, ^84^, ^85^] but are also the basis for conformational bias in azido‐functionalized nucleic acids, peptides, proteins, and carbohydrates. Future investigations will reveal whether azido‐based interactions have a similar potential to facilitate catalysis, as has been extensively and successfully explored for halogen and chalcogen bonding.

## Computational Details

All quantum‐mechanical calculations were performed with the ORCA 4.2.1[^86^, ^87^] (DFT, MP2, DLPNO‐CCSD(T), DID^[88]^), MOL‐PRO2015.1[^89^, ^90^] (W2‐F12, W1‐F12), TURBOMOLE 7.3.1[^91^, ^92^] (ESP) and xtb 6.2.1^[93]^ (conformational search, GFN2‐xTB) program packages. All adduct and monomer structures were optimized by using spin‐component‐scaled second‐order Møller–Plesset Perturbation Theory (SCS‐MP2), applying the large def2‐QZVPP basis set; for the larger conformer geometries, the def2‐TZVPP basis set was employed. LED analyses in the DLPNO‐CCSD(T)/def2‐QZVPP framework were conducted with VeryTightPNO settings. An automated conformer search was conducted with the CREST[^79^, ^80^] program by applying the iMTD‐GC algorithm with the GFN2‐xTB tight‐binding semiempirical method. Energetic presorting of the conformer rotamer ensemble was performed with the ENSO[^94^, ^95^] script at the PBEh‐3c level of theory (free energies obtained by inclusion of thermostatistical and zero‐point vibrational energy corrections). Final conformational free energies were calculated at the DLPNO‐CCSD(T)/CBS//SCS‐MP2/def2‐TZVPP level with Very‐TightPNO settings. The basis set extrapolation method used for DLPNO‐CCSD(T) association energies was a two‐point extrapolation scheme, which was applied to single‐point energies calculated with the def2‐TZVPP and def2‐QZVPP basis sets. *α*_34_ and *β*_34_ parameters for extrapolating the SCF energy and the correlation energy were 7.88 and 2.97, respectively, as suggested by Valeev et al.^[46]^ W2/W1‐F12 calculations were conducted according to the protocol suggested in the respective original publications.^[43]^

## Conflict of interest

The authors declare no conflict of interest.

## Supporting information

As a service to our authors and readers, this journal provides supporting information supplied by the authors. Such materials are peer reviewed and may be re‐organized for online delivery, but are not copy‐edited or typeset. Technical support issues arising from supporting information (other than missing files) should be addressed to the authors.

Supplementary

Supplementary
