# Supplementary material for: Quantification of Noncovalent Interactions in Azide–Pnictogen, –Chalcogen, and –Halogen Contacts
Source: Chemistry. 2021 Feb 8;27(14):4627–39. doi: 10.1002/chem.202004525 (PMC7986704; doi:10.1002/chem.202004525)
Supplement: Supplementary file 2 — Supplementary [file CHEM-27-4627-s001.pdf]

Chemistry–A European Journal

Supporting Information

Quantification of Noncovalent Interactions in Azide–Pnictogen, –Chalcogen, and –Halogen Contacts

Markus Bursch,*[a] Lukas Kunze+,[a] Amol M. Vibhute+,[b] Andreas Hansen,[a]
Kana M. Sureshan,[c] Peter G. Jones,[d] Stefan Grimme,*[a] and Daniel B. Werz*[b]

Contents

1 Synthesis and Characterization of New Azide-Containing Compounds **S-5**

1.1 General Information S-5

1.2 Synthesis of S2 . . . . . S-5

1.3 Synthesis of A S-7

1.4 Synthesis of B S-9

1.5 Synthesis of C S-11

1.6 Synthesis of D S-13

1.7 Synthesis of E and F S-15

1.8 Crystal Structure Determination S-19

2 Quantification of the Interaction by QM Methods **S-25**

2.1 Computational Details S-25

2.2 Statistical measures S-26

2.3 Local Energy Decomposition S-27

2.4 Association Energies S-28

2.5 Potential Energy Surface Scans S-31

2.6 Local Energy Decomposition Scans S-34

2.7 NBO SOPT Hydrogen Bonding Estimate S-37

2.8 Local Energy Decomposition for the Dimer Systems S-39

2.9 DFT-D4 Dispersion Energy vs LED Dispersion Energy (Dimer Systems) S-40

2.10 DFT-D4 Corrected DFAs vs DLPNO-CCSD(T) Scans S-41

2.11 DFT-D4 Dispersion Energy vs LED Dispersion Energy (Scans) S-43

2.12 Gas Phase Conformational Study S-45

2.13 LED Analysis of Constrained X-ray Structure Fragments S-46

3 References **S-48**

List of Tables

S1 Crystallographic data and structure refinement details for compounds **A**, **B** and **C**. S-20

S2 Crystallographic data and structure refinement details for compounds **D**, **E** and **F**. S-21

S3 Association energies of systems **1-44** for various methods in kcal·mol^{-1}. Except for semi-empirical, composite and F12 methods the def2-QZVPP basis set was used. S-28

S4 Association energies of systems **1-44** for various methods in kcal·mol^{-1}. Except for semi-empirical, composite and F12 methods the def2-QZVPP basis set was used. S-29

S5 Association energies of systems **1-44** for various methods in kcal·mol^{-1}. Except for semi-empirical, composite and F12 methods the def2-QZVPP basis set was used. S-30

S6 Interaction energies of the potential energy surface scan of system **1** for various methods at N···N2 distances between 1.8 and 6.0 Å, energies in kcal·mol^{-1}. DLPNO-CCSD(T)/CBS is abbreviated as DLPNO/CBS. S-31

S7 Interaction energies of the potential energy surface scan of system **11** for various methods at O···N2 distances between 1.8 and 6.0 Å, energies in kcal·mol^{-1}. DLPNO-CCSD(T)/CBS is abbreviated as DLPNO/CBS. S-32

S8 Interaction energies of the potential energy surface scan of system **35** for various methods at F···N2 distances between 1.8 and 6.0 Å, energies in kcal·mol^{-1}. DLPNO-CCSD(T)/CBS is abbreviated as DLPNO/CBS. S-33

S9 Local Energy Decomposition analysis contributions to the DLPNO-CCSD(T) interaction energy of system **1** at N···N2 distances between 1.8 and 6.0 Å, energies in kcal·mol^{-1}. S-34

S10 Local Energy Decomposition analysis contributions to the DLPNO-CCSD(T) interaction energy of system **11** at O···N2 distances between 1.8 and 6.0 Å, energies in kcal·mol^{-1}. S-35

S11 Local Energy Decomposition analysis contributions to the DLPNO-CCSD(T) interaction energy of system **35** at F···N2 distances between 1.8 and 6.0 Å, energies in kcal·mol^{-1}. S-36

S12 Dimer interaction energies for all systems at PBE0-D4/def2-QZVPP and DLPNO-CCSD(T)/def2-QZVPP level of theory, NBO SOPT estimate of the hydrogen bonding contribution to the interaction energy at the PBE0-D4/def2-QZVPP level and a scaled estimate $SOPT_{scaled}$(PBE0-D4), the scaling factor results from the ratio of E_{int}(LED) to E_{int}(PBE0-D4), the last line gives the mean values, all values in kcal·mol^{-1}. S-37
S13 Local Energy Decomposition analysis contributions to the DLPNO-CCSD(T) interaction energy of all dimer systems in kcal·mol^{-1}. The last column shows the ratio between the dispersion contribution and the total interaction energy. . S-39
S14 B3LYP-D4 dispersion corrections for the dimer and both fragments of the systems **1-44** as well as the resulting D4 energy differences, unscaled and scaled by 1.25 respectively. The last column shows the LED dispersion contribution, all values in kcal·mol^{-1}. S-40
S15 Interaction energy for system **13** for various DFAs and DLPNO-CCSD(T) at O$\cdots$N2 distances between 1.8 and 6.0 Å, all energies in kcal·mol^{-1}. S-41
S16 Interaction energy for system **5** for various DFAs and DLPNO-CCSD(T) at P$\cdots$N2 distances between 1.8 and 6.0 Å, all energies in kcal·mol^{-1}. S-42
S17 D4 dispersion energy differences for system **13** at O$\cdots$N2 distances between 1.8 and 6.0 Å, all energies in kcal·mol^{-1}. The last column shows the LED dispersion energy difference for comparison. S-43
S18 D4 dispersion energy differences for system **5** at P$\cdots$N2 distances between 1.8 and 6.0 Å, all energies in kcal·mol^{-1}. The last column shows the LED dispersion energy difference for comparison. S-44
S19 Relative free energies of gas phase conformers for **A** and **B**, final rerank at the DLPNO-CCSD(T)/CBS//SCS-MP2/def2-TZVPP level of theory, duplicate conformers (generated by reoptimization) were sorted out, all energies in kcal·mol^{-1}. S-45
S20 LED analysis on intermolecular model systems for X-ray structures **A** and **B** at DLPNO-CCSD(T)/def2-QZVPP//SCS-MP2/def2-QZVPP, the descriptor (X,Y) indicates if the fragments (PCH and azide moiety) were saturated with hydrogen or methyl groups. **B(Me-rep.)** is a methane dimer obtained by constrained optimization of hydrogen-saturated methyl groups from system **B(Me,Me)** and gives an estimate of the methyl group repulsion, all values in kcal·mol^{-1}. S-46

1 Synthesis and Characterization of New Azide-Containing Compounds

1.1 General Information

Solvents were purchased as synthetic grade solvents and stored over suitable molecular sieves. All reactions were performed in oven-dried glassware, septum-capped and under atmospheric pressure of argon. Commercially available compounds were used without further purification unless otherwise stated. Proton (^{1}H) and carbon (^{13}C) NMR spectra were recorded on a Bruker Avance II 300, a Bruker Avance III 400, a Bruker Avance III HD 500, a Bruker Avance II 600 and a Bruker Ascend 300 spectrometer using the signals from tetramethylsilane as internal references for 1H and 13C chemical shifts, respectively. Abbreviations for multiplicities were used as following s = singlet, d = doublet, t = triplet, m = multiplet. HRMS (ESI) mass spectrometry was carried out on a FTICR instrument and IR spectra were received from an ATR spectrometer. Because of potential danger of the azide-containing compounds on heating **melting points were not determined**.

1.2 Synthesis of S2

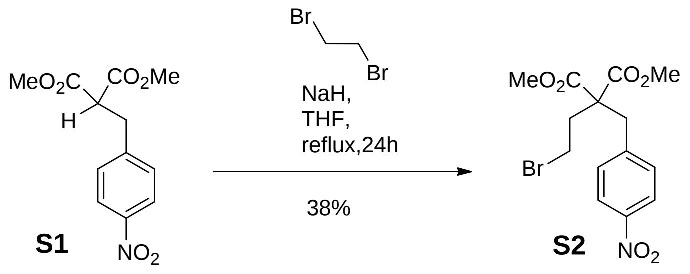

To a stirred solution of dimethyl dicarboxylate **S1** (2.0 g, 7.48 mmol) and 1,2-dibromo- ethane (1.93 mL, 22.45 mmol) in anhydrous THF (50 mL), sodium hydride (0.389 g, 9.73 mmol, 60% in mineral oil) was added at room temperature under inert atmosphere. The reaction was refluxed for 24 h. When TLC showed complete disappearance of the starting material, the reaction mixture was carefully quenched with water. The THF was evaporated under reduced pressure and the resulting residue was dissolved in ethyl acetate. The organic layer was washed with water frequently and finally with brine. The combined organic layer was dried over anhydrous Na_2SO_4 and concentrated under reduced pressure. The crude product was purified by

flash column chromatography (silica gel, 10:1 pentane/ethyl acetate) to get the desired product **S2** (1.06 g, 38%) as colorless liquid.

^{1}H NMR (500 MHz, $CDCl_3$) δ = 8.16 (d, J = 8.8, 2H), 7.28 (d, J = 8.9 Hz, 2H), 3.75 (s, 6H), 3.61 - 2.85 (m, 4H), 2.65 - 2.09 (m, 2H).

^{13}C NMR (126 MHz, $CDCl_3$) δ = 170.09, 147.29, 143.02, 130.81, 123.62, 58.82, 52.90, 39.46, 36.69, 26.50, −0.01.

IR (ATR): $\tilde{\nu}$ (cm^{-1}) = 2954, 2858, 1726, 1603, 1519, 1448, 1378, 1344, 1307, 1264, 1220, 1167, 1106, 1061, 1019.

$C_{14}H_{16}BrNO_6$ (MW = 374.18) calcd: 396.00532, found: 396.00570 $[M+Na]^+$ (ESI-HRMS).

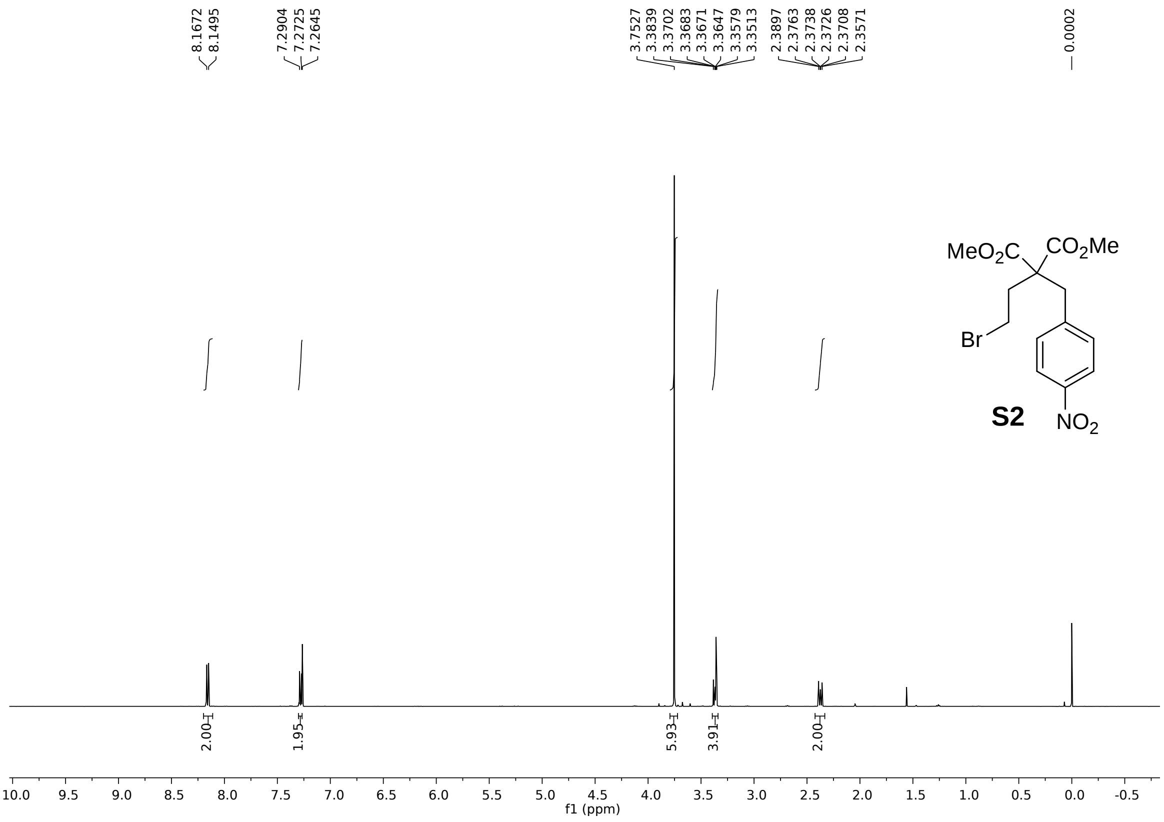

Figure 1.1: **^{1}H NMR** of **S2** in $CDCl_3$ at 500 MHz

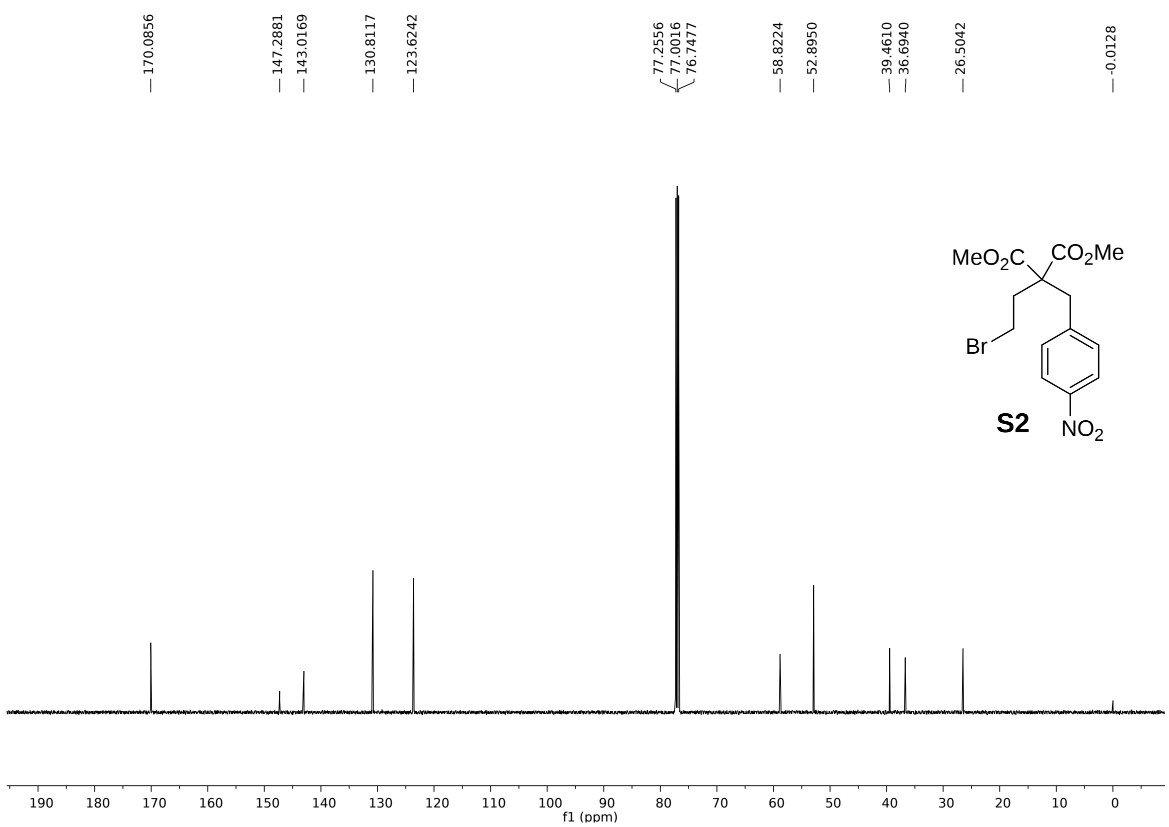

Figure 1.2: ^{1}**C NMR** of **S2** in $CDCl_3$ at 126 MHz

1.3 Synthesis of A

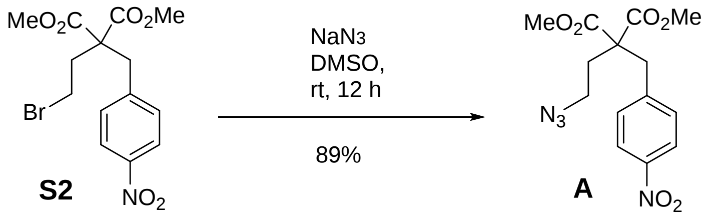

To a solution of bromide **S2** (0.89 g, 2.37 mmol), in anhydrous DMSO (100 mL), sodium azide (0.31 g, 4.75 mmol) was added at room temperature and the reaction was stirred at the same temperature for 12 h. When TLC (8:1, pentane/EtOAc) showed complete disappearance of starting material, the reaction mixture was extracted with ethyl acetate and washed with water several time to remove DMSO and finally with brine. The combined organic layer was dried over anhyd. Na_2SO_4 and concentrated under reduced pressure. The crude product thus obtained was purified by flash column chromatography to get the desired product as colorless solid. This solid was then re-crystallized from dichloromethane and pentane to get colorless needle-shaped crystals of **A** (0.713 g, 89%).

^{1}**H NMR** (400 MHz, $CDCl_3$) δ = 8.15 (d, J = 8.8 Hz, 2H), 7.29 (d, J = 8.9 Hz, 2H), 3.75 (s,

6H), 3.41 (t, J = 7.1 Hz, 2H), 3.38 (s, 2H), 2.08 (t, J = 7.1 Hz, 2H).

^{13}C NMR (101 MHz, $CDCl_3$) δ = 170.39, 147.22, 143.23, 130.82, 123.53, 77.32, 77.00, 76.68, 57.17, 52.79, 47.34, 39.25, 32.26.

IR (ATR): $\widetilde{\nu}$ (cm$_{-1}$) = 3120, 3084, 3025, 2957, 2846, 2103, 1726, 1600, 1508, 1438, 1342, 1265, 1204, 1098, 1034.

$C_{14}H_{16}N_4O_6$ (MW = 336.30) calcd: 359.09621,
found: 359.09644 $[M+Na]^+$ (ESI-HRMS).

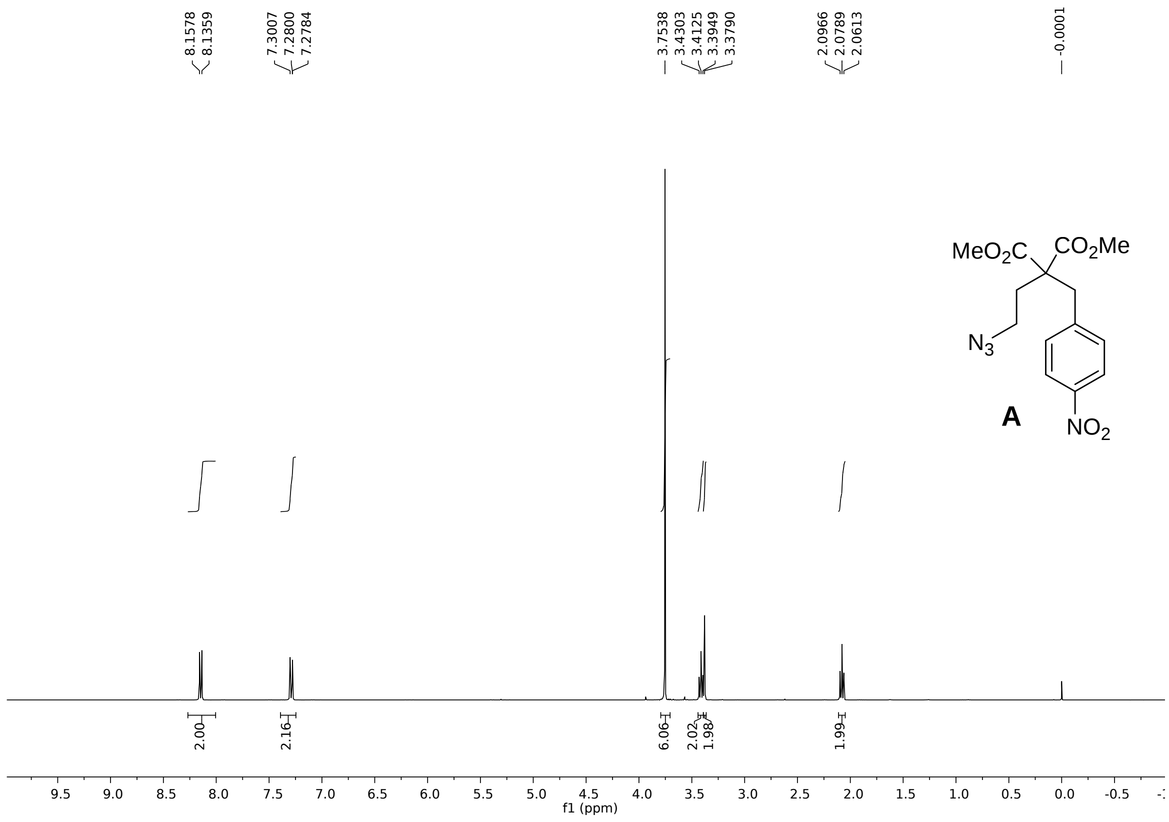

Figure 1.3: **^{1}H NMR** of **A** in $CDCl_3$ at 400 MHz

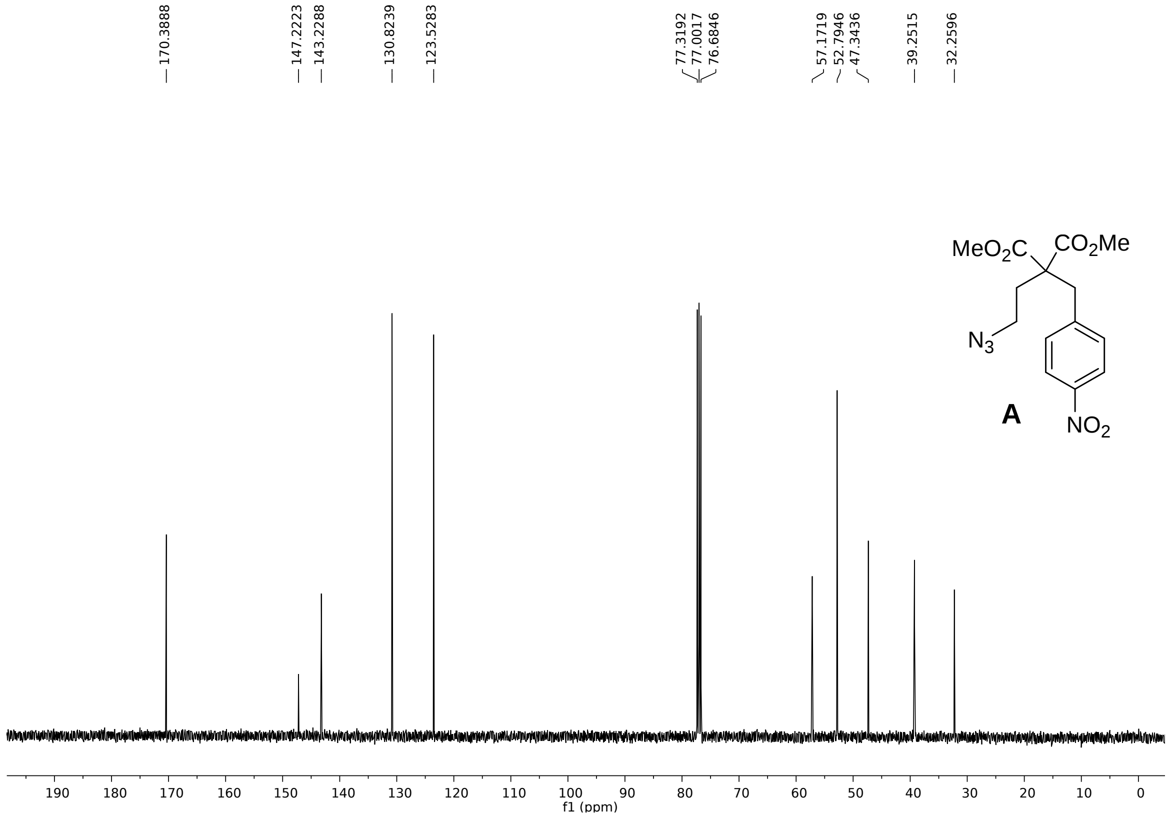

Figure 1.4: **^{13}C NMR** of **A** in $CDCl_3$ at 101 MHz

1.4 Synthesis of B

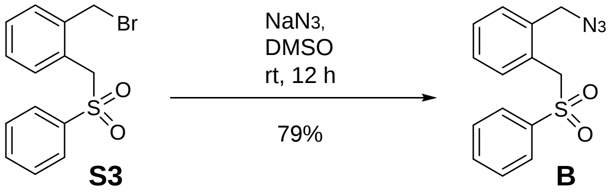

To a solution of commercially available bromide **S3** (0.30 g, 0.922 mmol), in anhydrous DMSO (20 mL), sodium azide (0.12 g, 1.840 mmol) was added at room temperature and the reaction was stirred at the same temperature for 12 h. When TLC (5:1, pentane/EtOAc) showed complete disappearance of starting material, the reaction mixture was extracted with ethyl acetate and washed with water several time to remove DMSO and finally with brine. The combined organic layer was dried over anhyd. Na_2SO_4 and concentrated under reduced pressure. The crude product thus obtained was purified by flash column chromatography to get desired product as colorless solid. This solid was then re-crystallized from dichloromethane and pentane to get colorless needle-shaped crystals of **B** (0.21 g, 79%).

^{1}H NMR (500 MHz, $CDCl_3$): δ = 7.74 - 7.68 (m, 2H), 7.67 - 7.59 (m, 1H), 7.53 - 7.43 (m, 2H), 7.42 - 7.28 (m, 2H), 7.28 - 7.18 (m, 1H), 7.04 (dd, J = 7.6, 1.3 Hz, 1H), 4.46 (s, 2H), 4.35 (s, 2H).

^{13}C NMR (126 MHz, $CDCl_3$): δ = 138.08, 135.60, 133.96, 132.88, 130.15, 129.40, 129.09, 128.62, 128.49, 126.73, 59.42, 52.55.

IR (ATR): $\tilde{\nu}$ (cm^{-1}) = 3063, 2084, 1487, 1446, 1354, 1298, 1248, 1144, 1082, 995, 880, 837, 749, 679, 617, 576.

$C_{14}H_{13}N_3O_2S$ (MW = 287.33) calcd: 310.06207,
found: 310.06214 $[M+Na]^+$ (ESI-HRMS).

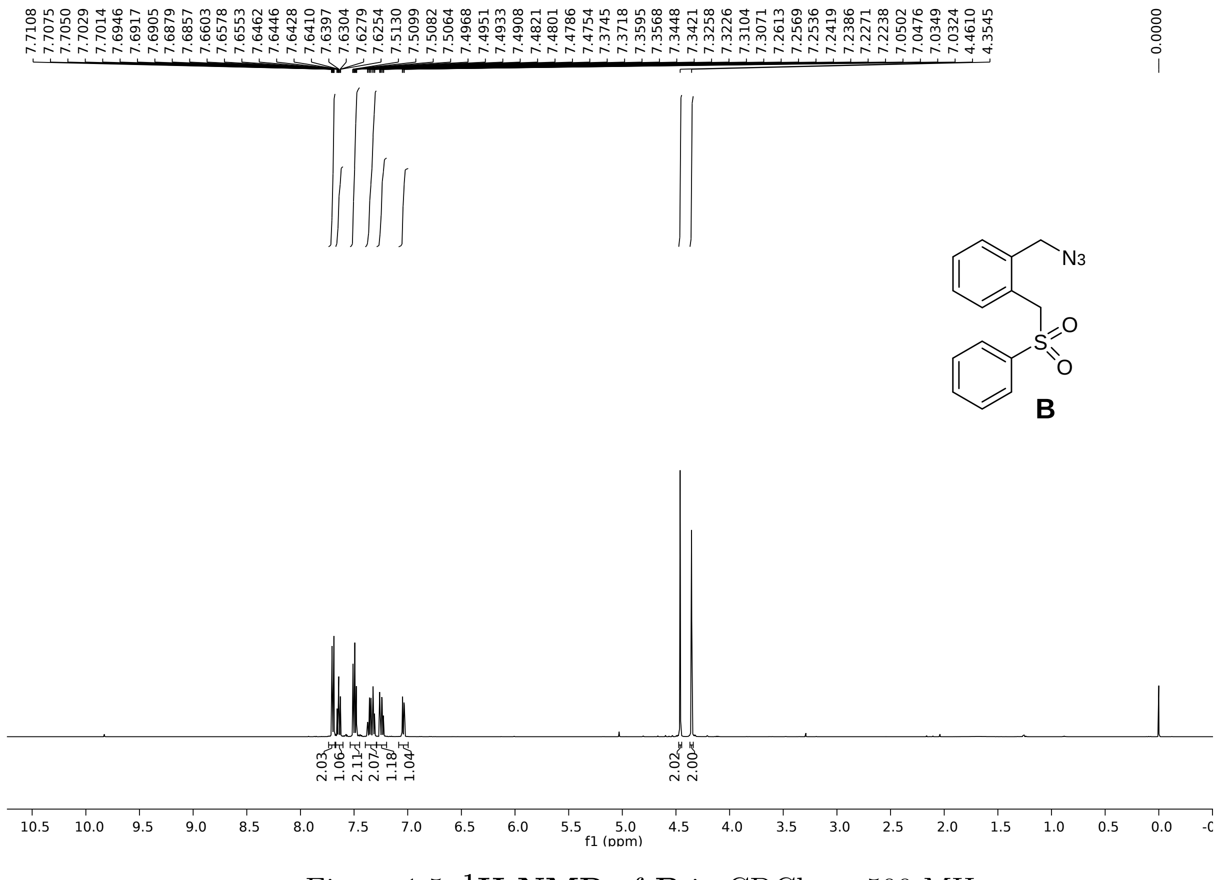

Figure 1.5: **^{1}H NMR** of **B** in $CDCl_3$ at 500 MHz

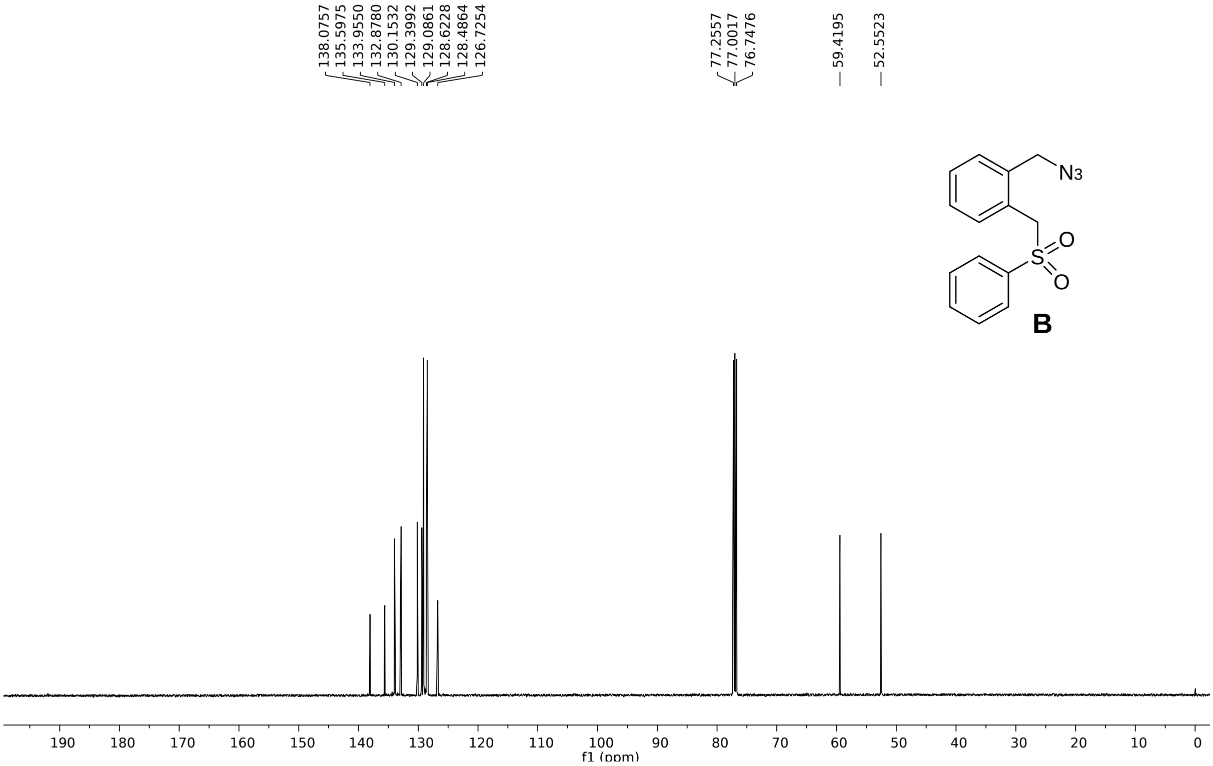

Figure 1.6: ^{13}**C NMR** of **B** in $CDCl_3$ at 126 MHz

1.5 Synthesis of C

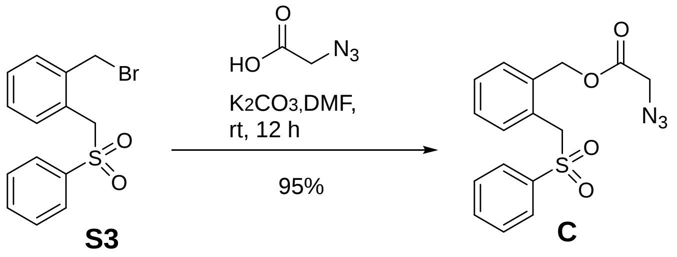

To a solution of bromide **S3** (0.20 g, 0.610 mmol), in anhydrous DMF (10 mL), azido acetic acid (0.093 g, 0.922 mmol) was added at room temperature. Anhydrous K_2CO_3 (0.127, 0.922 mmol) was added at room temperature and the reaction was stirred at the same temperature for 12 h. When TLC (6:1, pentane/EtOAc) showed complete disappearance of starting material, the reaction was quenched with addition of water. The solvents were removed under reduced pressure and the resulting residue was dissolved in ethyl acetate. The ethyl acetate layer was washed with water (2 times to remove traces of DMF) and brine. The combined organic layer was dried over anhyd. Na_2SO_4 and concentrated under reduced pressure. The crude product was purified by flash column chromatography to get the desired product **C** as

colorless solid. This solid was then re-crystalized from dichloromethane and pentane to get colorless needle-shaped crystals.

^{1}H NMR (600 MHz, $CDCl_3$) δ = 7.73 (dd, J = 8.4, 1.3 Hz, 2H), 7.66 (td, J = 7.4, 1.3 Hz, 1H), 7.52 (dd, J = 8.4, 7.4 Hz, 2H), 7.43 (dd, J = 7.7, 1.4 Hz, 1H), 7.37 (td, J = 7.5, 1.4 Hz, 1H), 7.30 - 7.23 (m, 1H), 7.05 (dd, J = 7.7, 1.3 Hz, 1H), 5.22 (s, 2H), 4.52 (s, 2H), 3.87 (s, 2H).

^{13}C NMR (151 MHz, $CDCl_3$) δ = 168.05, 138.24, 135.37, 133.98, 132.67, 130.97, 129.40, 129.17, 129.04, 128.51, 127.05, 64.72, 59.56, 50.27.

IR (ATR): $\tilde{\nu}$ (cm^{-1}) = 2985, 2939, 2192, 2103, 1741, 1310, 1295, 1195, 1132, 1082.

$C_{16}H_{15}N_3O_4S$ (MW = 345.37) calcd: 368.06755, found: 368.06764 $[M+Na]^+$ (ESI-HRMS).

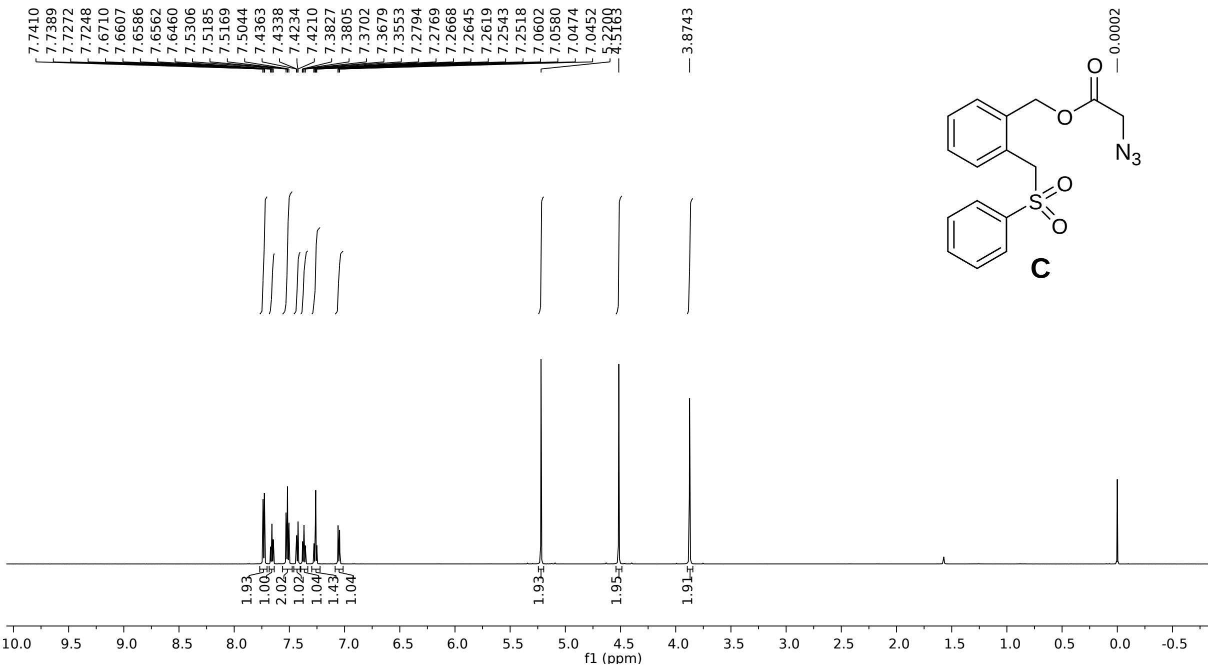

Figure 1.7: **^{1}H NMR** of **C** in $CDCl_3$ at 600 MHz

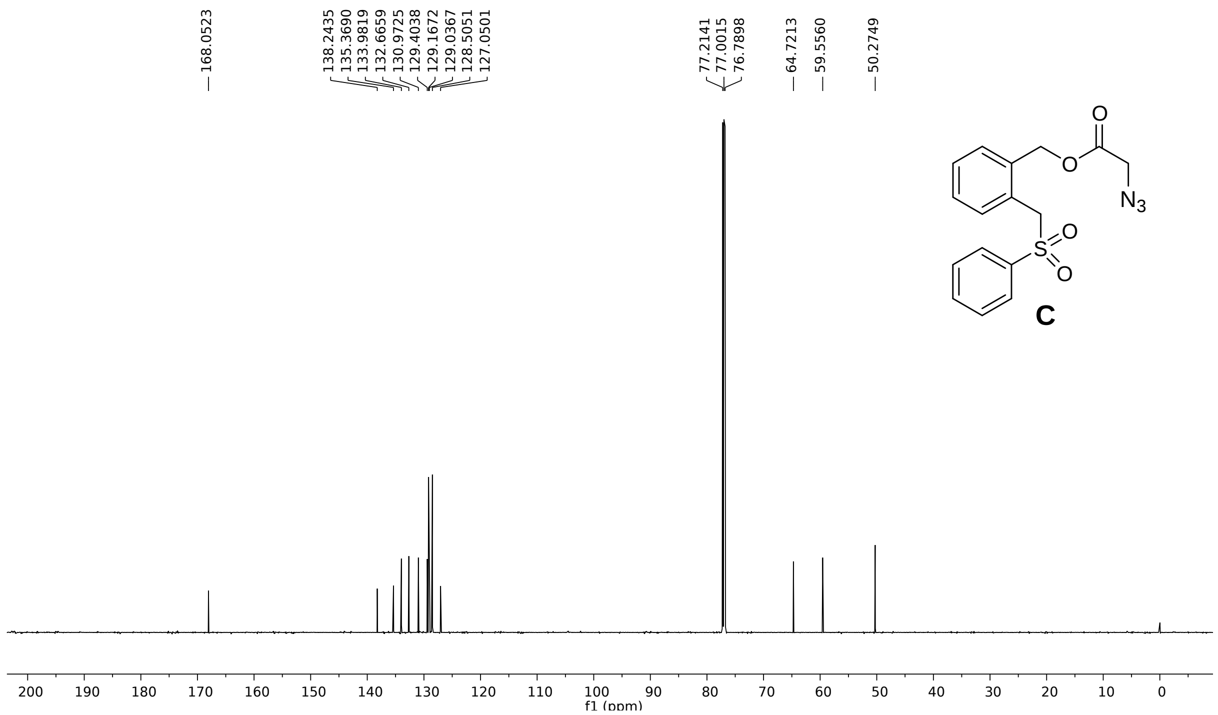

Figure 1.8: ^{13}**C NMR** of **C** in $CDCl_3$ at 151 MHz

1.6 Synthesis of D

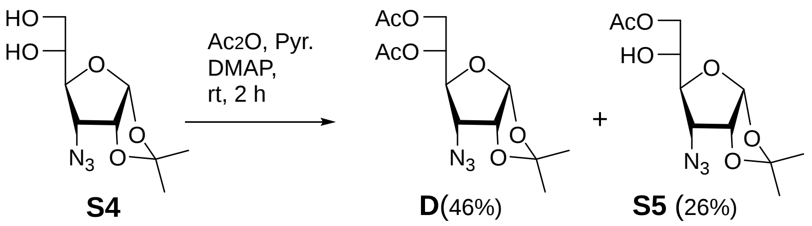

To a cooled solution of diol **S4** (0.30 g, 1.22 mmol) in anhydrous pyridine (15 mL), DMAP (0.002 g, 0.0122 mmol) followed by Ac_2O (0.17 mL, 1.83 mmol, 1.5 equiv) was added dropwise and the reaction was allowed to stir at room temperature for 2 h. When TLC (1:1, pentane/EtOAc) showed complete disappearance of starting material, the reaction mixture was concentrated under reduced pressure. The residue was extracted with ethyl acetate, washed with water several times to remove traces of pyridine and finally with brine. The combined organic layer was dried over anhyd. Na_2SO_4 and concentrated under reduced pressure. The crude product was purified by flash column chromatography to get diacetate product **D** (0.185 g, 46%) as a colorless solid and monoacetate product **S5** (0.092 g, 26%) as colorless oil. The diacetate product **D** was then re-crystallized from dichloromethane and pentane to get colorless needle-shaped crystals. Several attempts to crystallize monoacetate product **S5** failed, hence

S5 was excluded from this study.

^{1}H NMR (400 MHz, $CDCl_3$) δ = 5.79 (dd, J = 3.7, 0.7 Hz, 1H), 5.28 (tdd, J = 6.3, 3.5, 0.8 Hz, 1H), 4.75 (dd, J = 4.7, 3.7 Hz, 1H), 4.42 (ddd, J = 12.1, 3.5, 0.8 Hz, 1H), 4.29 - 4.03 (m, 2H), 3.43 (dd, J = 9.4, 4.7 Hz, 1H), 2.13 (s, 3H), 2.08 (s, 3H), 1.59 (s, 3H), 1.37 (s, 3H).

^{13}C NMR (101 MHz, $CDCl_3$) δ = 170.42, 169.97, 113.35, 103.99, 80.40, 75.19, 70.62, 62.45, 61.97, 26.40, 26.33, 20.75, 20.60.

IR (ATR): $\tilde{\nu}$ (cm^{-1}) = 2976, 2097, 1738, 1435, 1373, 1217, 1015.

$C_{13}H_{19}N_3O_7$ (MW = 329.30) calcd: 352.11152,
found: 352.11172 $[M+Na]^+$ (ESI-HRMS).

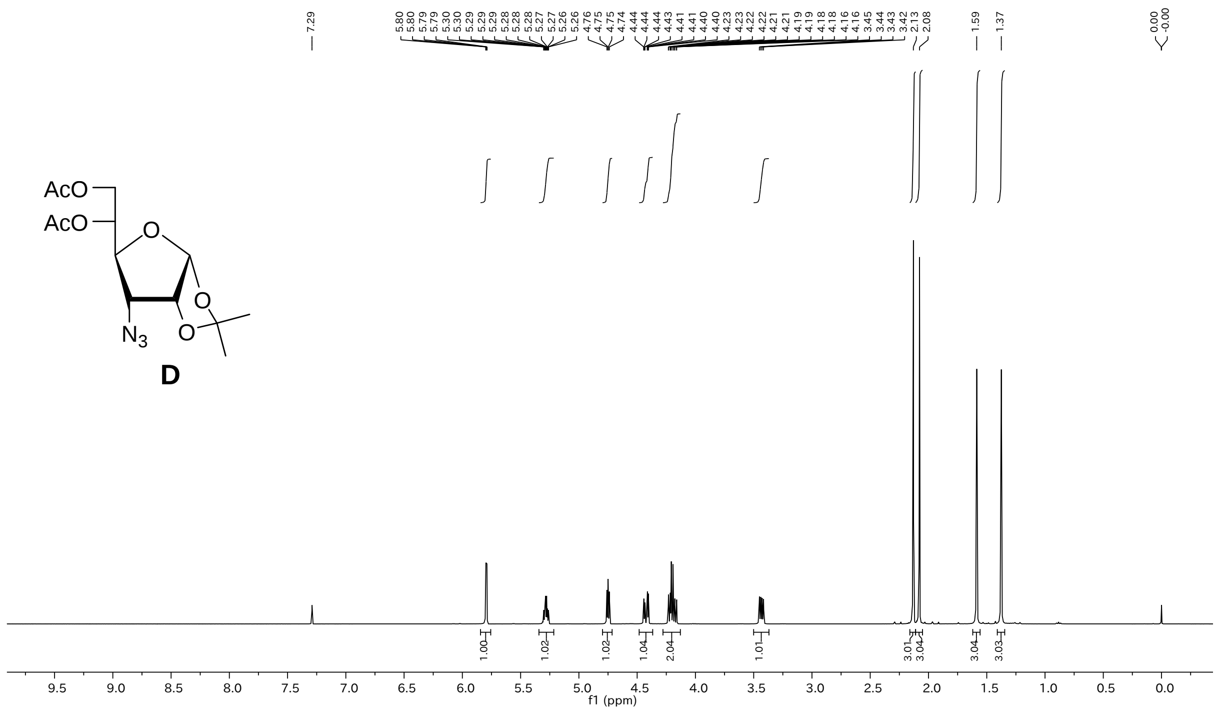

Figure 1.9: **^{1}H NMR** of **D** in $CDCl_3$ at 400 MHz

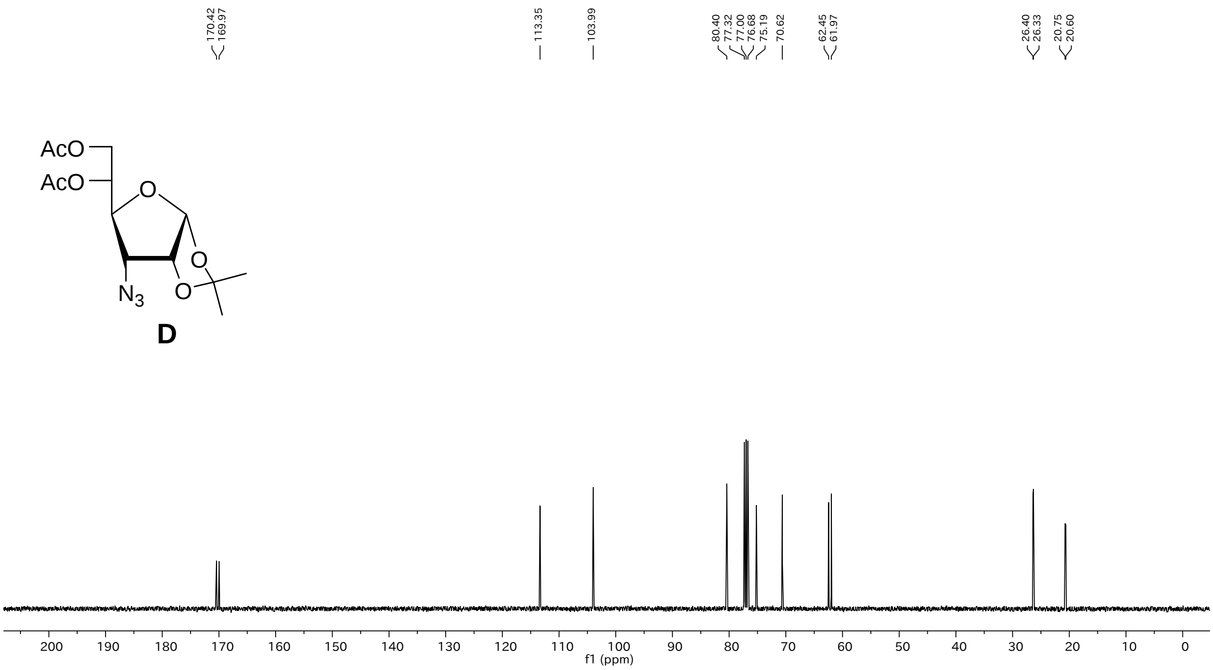

Figure 1.10: **^{13}C NMR** of **D** in $CDCl_3$ at 101 MHz

1.7 Synthesis of E and F

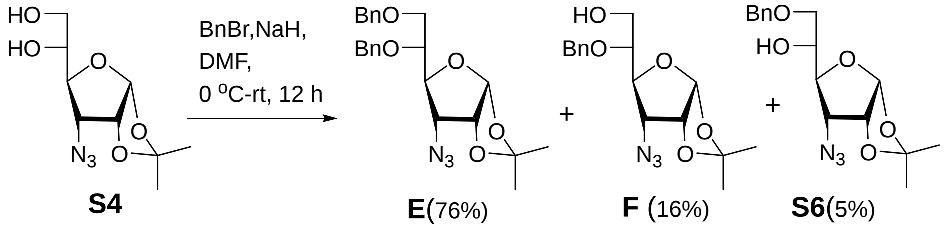

To a cooled solution of diol **S4** (2.0 g, 8.15 mmol) in anhydrous DMF (20 mL), NaH (0.489 g, 12.23 mmol, 1.5 equiv, 60% suspension in mineral oil) followed by BnBr (1.45 mL, 12.23 mmol, 1.5 equiv) was added dropwise and the reaction was allowed to stir at room temperature for 12 h. When TLC (5:1, pentane/EtOAc) showed complete disappearance of starting material, the reaction mixture was concentrated under reduced pressure. The residue was extracted with ethyl acetate, washed with water several times to remove traces of DMF and finally with brine. The combined organic layer was dried over anhyd. Na_2SO_4 and concentrated under reduced pressure. The crude product was purified by flash column chromatography to get dibenzyl ether product **E** (2.62 g, 76%) as colorless solid, monobenzyl ether product **F** (0.437 g, 16%) as colorless solid and dibenzyl ether product **S6** (0.136 g, 5%) as colorless oil. Dibenzyl **E** and monobenzyl **F** were then re-crystallized from dichloromethane and pentane to get colorless needle-shaped crystals for both the compounds. Several attempts to crystallize monobenzyl

product **S6** failed, hence **S6** was excluded from this study.

Data for E:

^{1}H NMR (400 MHz, $CDCl_3$) δ = 7.71 - 6.77 (m, 10H), 5.75 (d, J = 3.6 Hz, 1H), 4.88 - 4.65 (m, 3H), 4.63 - 4.42 (m, 2H), 4.32 (dd, J = 9.3, 3.0 Hz, 1H), 3.96 (td, J = 6.0, 3.0 Hz, 1H), 3.77 - 3.50 (m, 3H), 1.57 (s, 3H), 1.36 (s, 3H).

^{13}C NMR (101 MHz, $CDCl_3$) δ = 138.37, 137.94, 128.37, 128.31, 127.77, 127.69, 127.66, 127.63, 113.13, 103.97, 80.84, 78.18, 76.92, 73.68, 73.48, 69.64, 60.02, 26.58, 26.51.

IR (ATR): $\tilde{\nu}$ (cm^{-1}) = 2987, 2884, 2100, 1375, 1261, 1025.

$C_{23}H_{27}N_3O_5$ (MW = 425.47) calcd: 448.18482,
found: 448.18441 $[M+Na]^+$ (ESI-HRMS).

Data for F:

^{1}H NMR (400 MHz, $CDCl_3$) δ = 7.71 - 6.77 (m, 5H), 5.74 (d, J = 3.6 Hz, 1H), 4.94 - 4.50 (m, 3H), 4.22 (dd, J = 9.4, 3.6 Hz, 1H), 3.81 (td, J = 5.5, 3.6 Hz, 1H), 3.72 (d, J = 5.6 Hz, 2H), 3.55 (dd, J = 9.4, 4.8 Hz, 1H), 2.22 (s, 1H), 1.57 (s, 3H), 1.36 (s, 3H).

^{13}C NMR (101 MHz, $CDCl_3$) δ = 137.09, 127.51, 126.91, 126.87, 112.21, 102.90, 79.59, 77.88, 77.00, 72.68, 60.58, 59.65, 25.53, 25.47.

IR (ATR): $\tilde{\nu}$ (cm^{-1}) = 3458, 3084, 2991, 2878, 2102, 1374, 1260, 1208, 1157, 1109, 1014.

$C_{16}H_{21}N_3O_5$ (MW = 335.35) calcd: 358.13734,
found: 358.13749 $[M+Na]^+$ (ESI-HRMS).

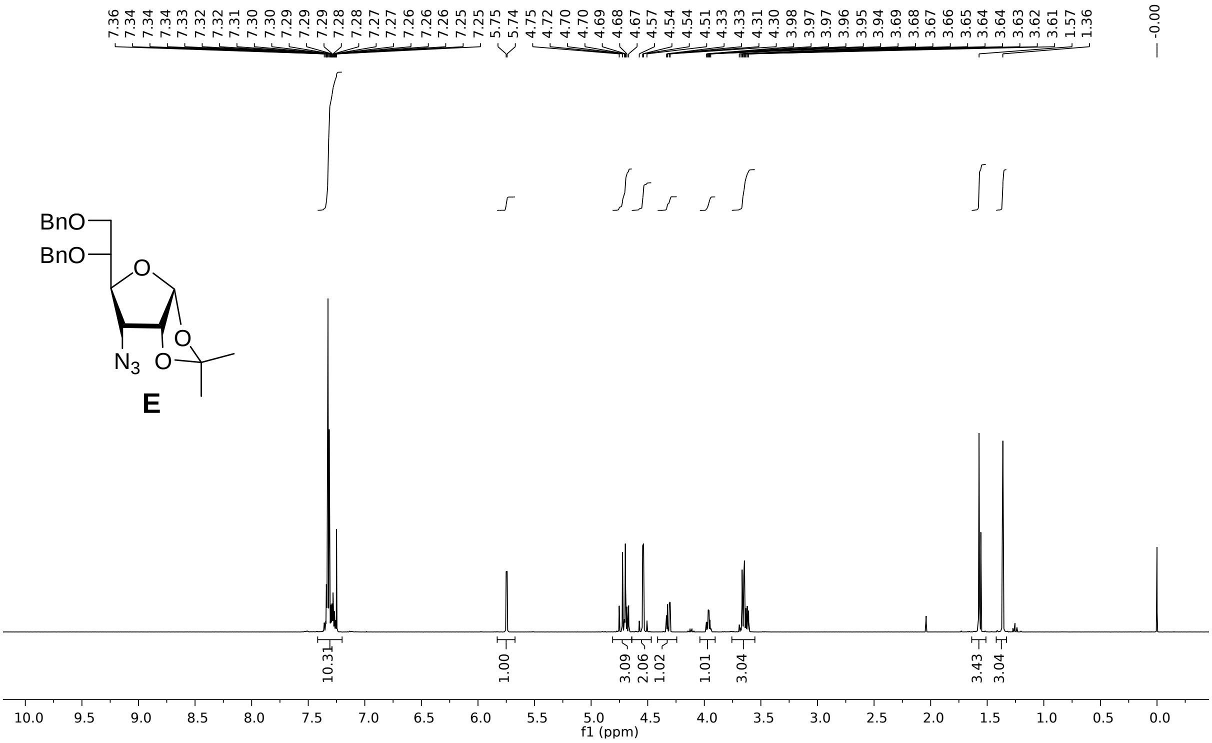

Figure 1.11: **^{1}H NMR** of **E** in $CDCl_3$ at 400 MHz

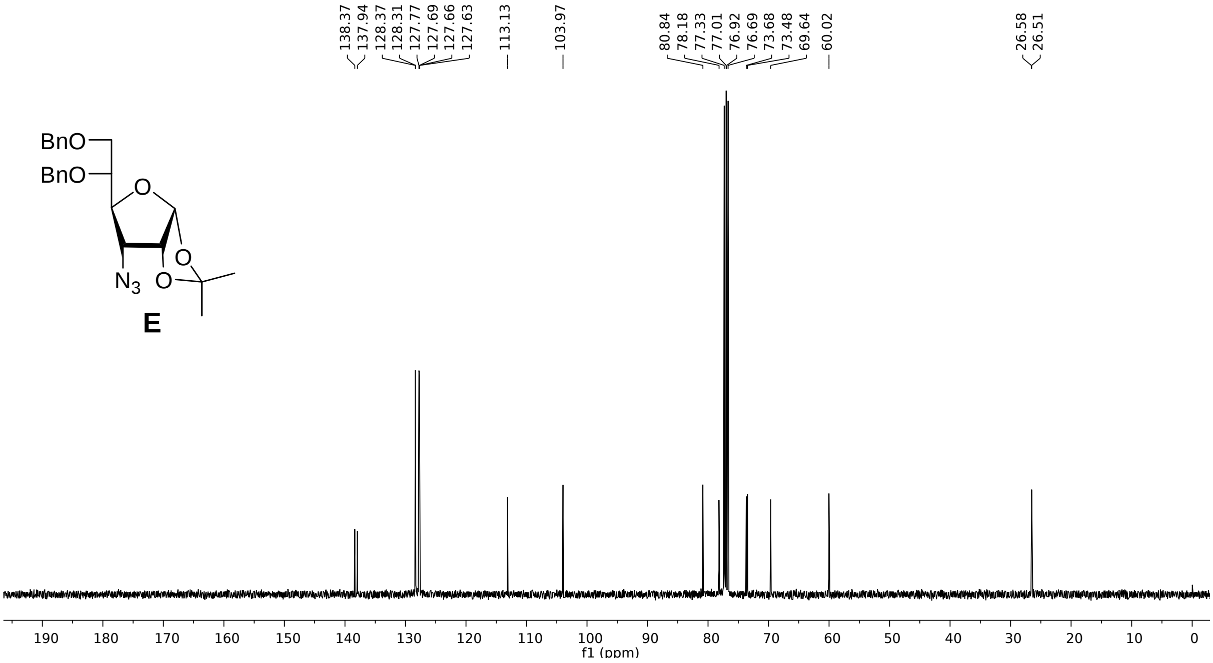

Figure 1.12: **^{13}C NMR** of **E** in $CDCl_3$ at 101 MHz

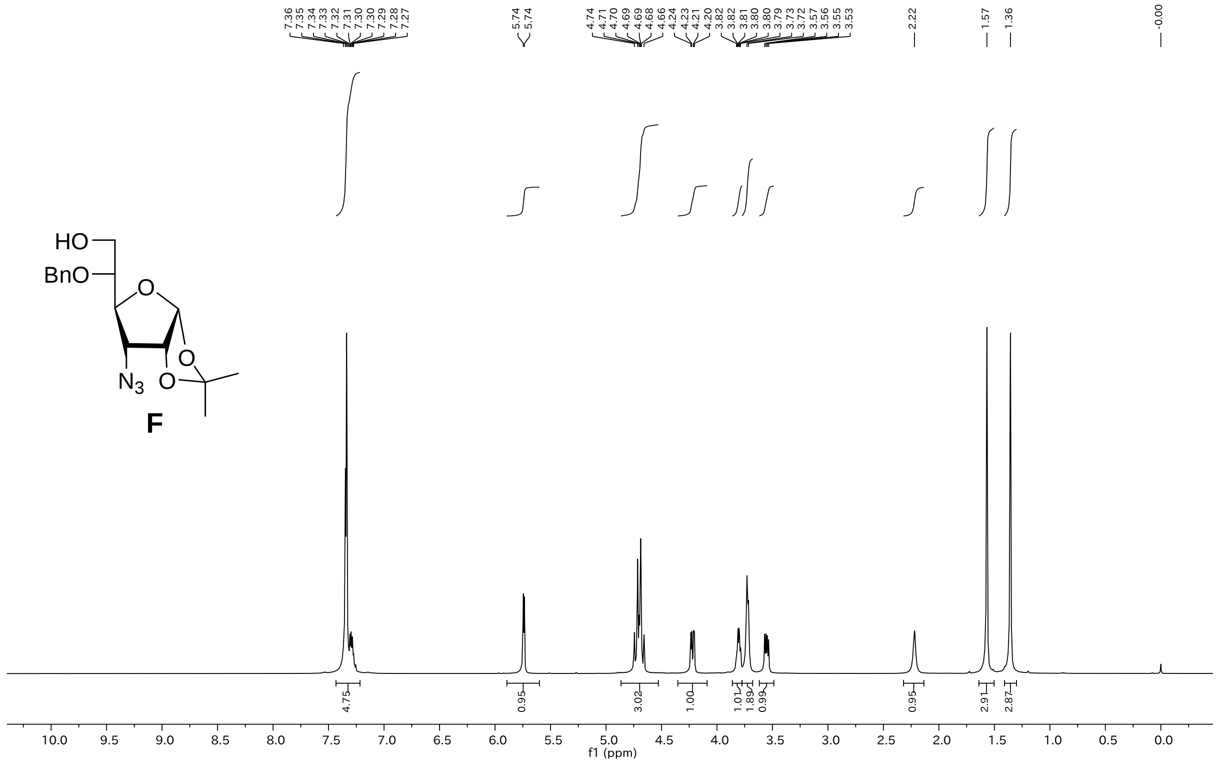

Figure 1.13: **^{1}H NMR** of **F** in $CDCl_3$ at 400 MHz

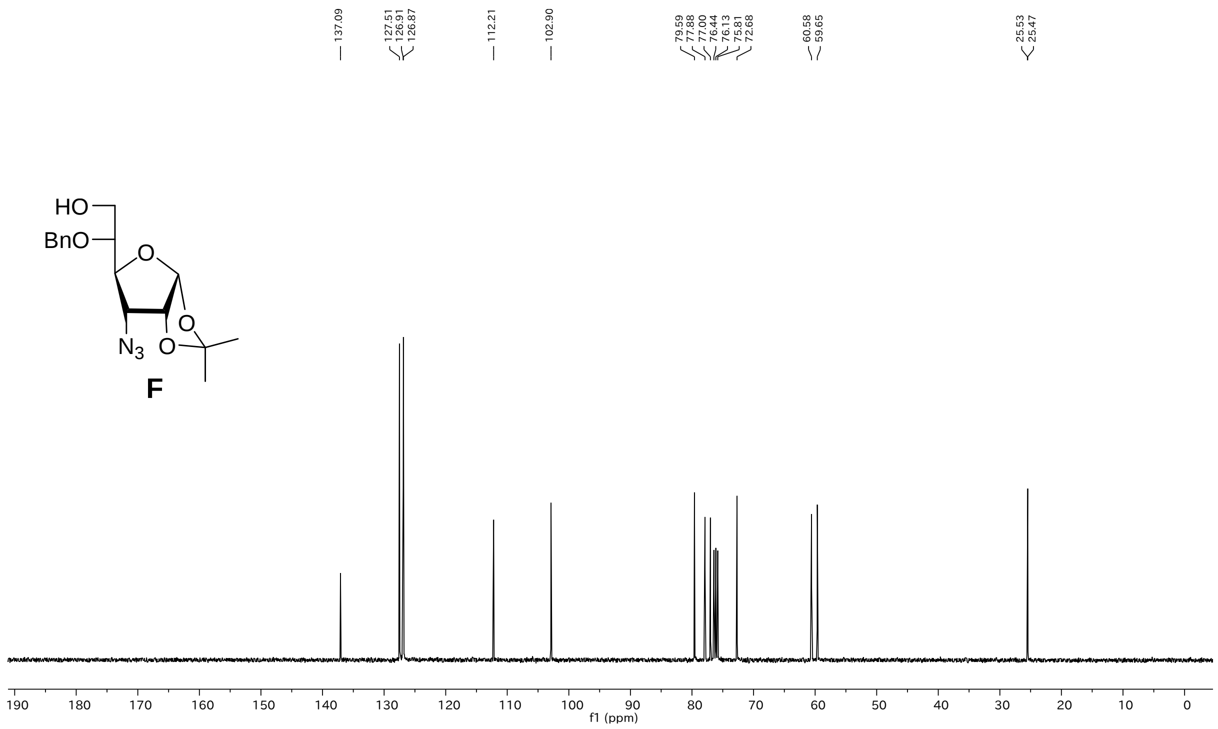

Figure 1.14: **^{13}C NMR** of **F** in $CDCl_3$ at 101 MHz

1.8 Crystal Structure Determination

Crystals were mounted in inert oil on glass fibres or nylon loops and transferred to the cold gas stream of the diffractometer (**A**-**C**, Rigaku/Oxford XtaLAB Synergy using mirror-focussed Cu Kα or Mo Kα radiation; **D**-**F**: Oxford Diffraction Nova A using mirror-focussed Cu Kα radiation). Absorption corrections were implemented on the basis of multi-scans. The structures were refined anisotropically on F^2 using the programs SHELXL-2017 or -2018[1]. Hydrogen atoms of OH groups were refined freely; other hydrogens were included using rigid methyl groups or a riding model starting from calculated positions.

Special features: Compounds **D**-**F** are pure enantiomers. Compound **B** is achiral but crystallized by chance in a chiral space group. The unambiguous Flack parameters confirmed the absolute configurations of **D**-**F** and assigned an absolute structure to compound **B**.

Crystallographic data are summarized in tables S1 and S2. Additionally, complete data have been deposited with the Cambridge Crystallographic Data Centre under the numbers CCDC 1994147-1994152. Copies of the data can be obtained free of charge from www.ccdc.cam.ac.uk/data_request/cif.

Table S1: Crystallographic data and structure refinement details for compounds **A**, **B** and **C**.

Compound	**A**	**B**	**C**
CCDC number	1994147	1994148	1994149
Formula	$C_{14}H_{16}N_4O_6$	$C_{14}H_{13}N_3O_2S$	$C_{16}H_{15}N_3O_4S$
M_r	336.31	287.33	345.37
Cryst. size (mm)	0.25 x 0.2 x 0.15	0.2 x 0.2 x 0.08	0.2 x 0.1 x 0.1
Crystal system	monoclinic	orthorhombic	monoclinic
Space group	$P2_1/n$	$P2_12_12_1$	$P2_1/n$
Temperature (°C)	−173	−173	−173
a (Å)	7.9911(3)	5.24853(16)	11.93490(12)
b (Å)	6.9594(3)	11.7841(4)	5.34969(6)
c (Å)	27.4823(10)	21.2140(7)	24.6264(3)
α (°)	90	90	90
β (°)	97.780(3)	90	97.0698(10)
γ (°)	90	90	90
V (Å^3)	1514.3	1312.07	1560.39
Z	4	4	4
D_x (Mg m^{-3})	1.475	1.455	1.47
λ (Å)	0.71073	0.71073	1.54184
μ (mm^{-1})	0.12	0.25	2.1
Transmissions	0.925 – 1.000	0.925 – 1.000	0.750 – 1.000
F(000)	704	600	720
$2\theta_{max}$	67.6	68	155.3
Refl. measured	59578	51782	48129
Refl. indep.	5539	4891	3302
R_{int}	0.034	0.035	0.037
Parameters	219	181	217
Restraints	0	0	0
$wR(F^2$, all refl.)	0.095	0.068	0.08
$R(F, >4\sigma(F))$	0.035	0.026	0.03
Flack parameter	–	−0.005(15)	–
S	1.04	1.05	1.09
Max. $\Delta\rho$ (e Å^{-3})	0.47, −0.26	0.33, −0.28	0.25, −0.49

Table S2: Crystallographic data and structure refinement details for compounds **D**, **E** and **F**.

Compound	**D**	**E**	**F**
CCDC number	1994150	1994151	1994152
Formula	$C_{13}H_{19}N_3O_7$	$C_{23}H_{27}N_3O_5$	$C_{16}H_{21}N_3O_5$
M_r	329.31	425.48	335.36
Cryst. size (mm)	0.2 x 0.1 x 0.06	0.25 x 0.2 x 0.06	0.25 x 0.25 x 0.15
Crystal system	monoclinic	monoclinic	monoclinic
Space group	$P2_1$	$P2_1$	$C2$
Temperature (°C)	−172	−173	−173
a (Å)	5.30735(11)	17.5539(2)	21.4349(4)
b (Å)	14.31292(18)	9.15924(7)	5.39906(10)
c (Å)	10.41010(14)	28.2584(3)	16.8187(4)
α (°)	90	90	90
β (°)	91.9918(14)	107.2470(12)	120.197(3)
γ (°)	90	90	90
V (Å^3)	790.31	4339.11	1682.28
Z	2	8	4
D_x (Mg m^{-3})	1.384	1.303	1.324
λ (Å)	1.54184	1.54184	1.54184
μ (mm^{-1})	0.97	0.76	0.83
Transmissions	0.834 – 1.000	0.893 – 1.000	0.643 – 1.000
$F(000)$	348	1808	712
$2\theta_{max}$	152.4	152.4	152.4
Refl. measured	32785	179933	33922
Refl. indep.	3307	18100	3390
R_{int}	0.043	0.053	0.35
Parameters	212	1125	224
Restraints	1	1	1
$wR(F^2$, all refl.)	0.070	0.080	0.067
$R(F, >4\sigma(F))$	0.027	0.031	0.025
Flack parameter	0.00(10)	0.09(6)	0.03(10)
S	1.04	1.03	1.07
Max. $\Delta\rho$ (e Å^{-3})	0.15, −0.19	0.24, −0.18	0.26, −0.14

Thermal ellipsoid plots

(at the 50% probability level unless otherwise stated)

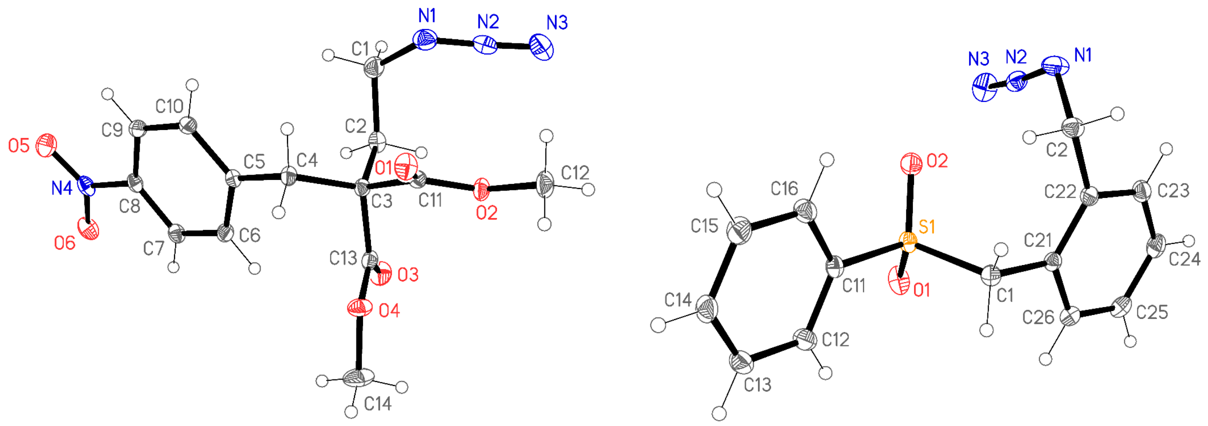

Figure 1.15: Structure of compound **A** (left) and **B** (right) in the crystal.

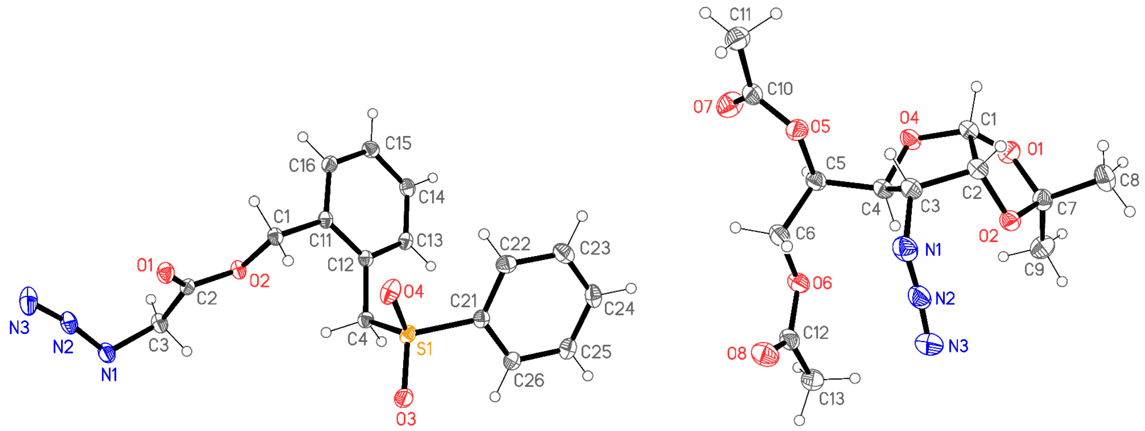

Figure 1.16: Structure of compound **C** (left) and **D** (right) in the crystal.

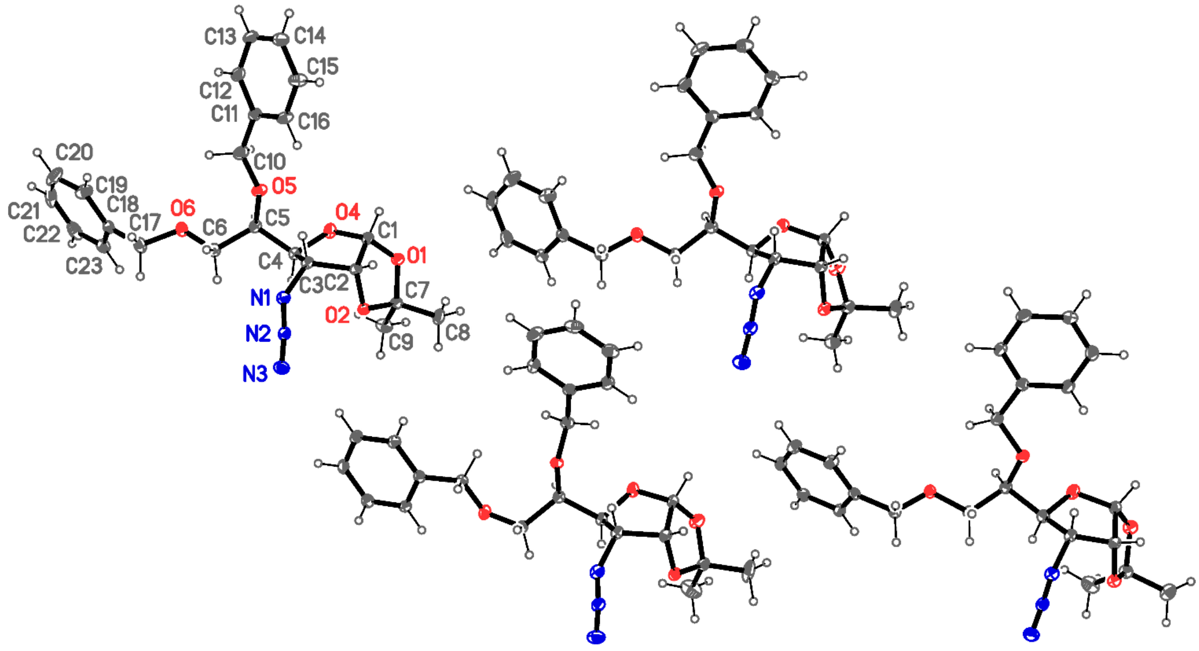

Figure 1.17: Structure of compound **E** in the crystal. Ellipsoids represent 30% probability levels. Only the first molecule is labelled; the others are numbered analogously, but with suffixes (‘, “, *) for molecules 2.4 respectively. The four independent molecules differ considerably in the orientation of their -OCH_2Ph side chains (see CIF files for details of torsion angles).

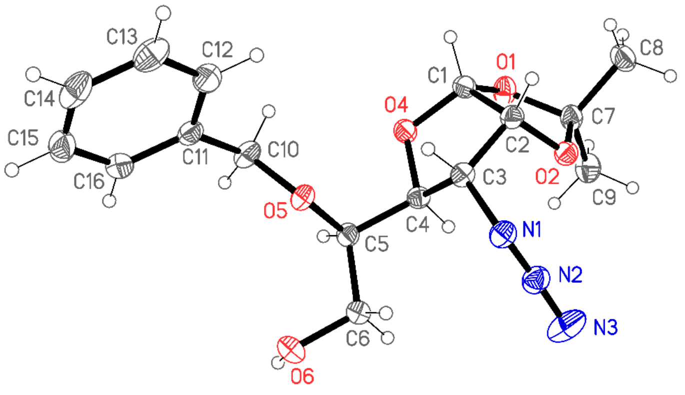

Figure 1.18: Structure of compound **F** in the crystal.

Packing diagram

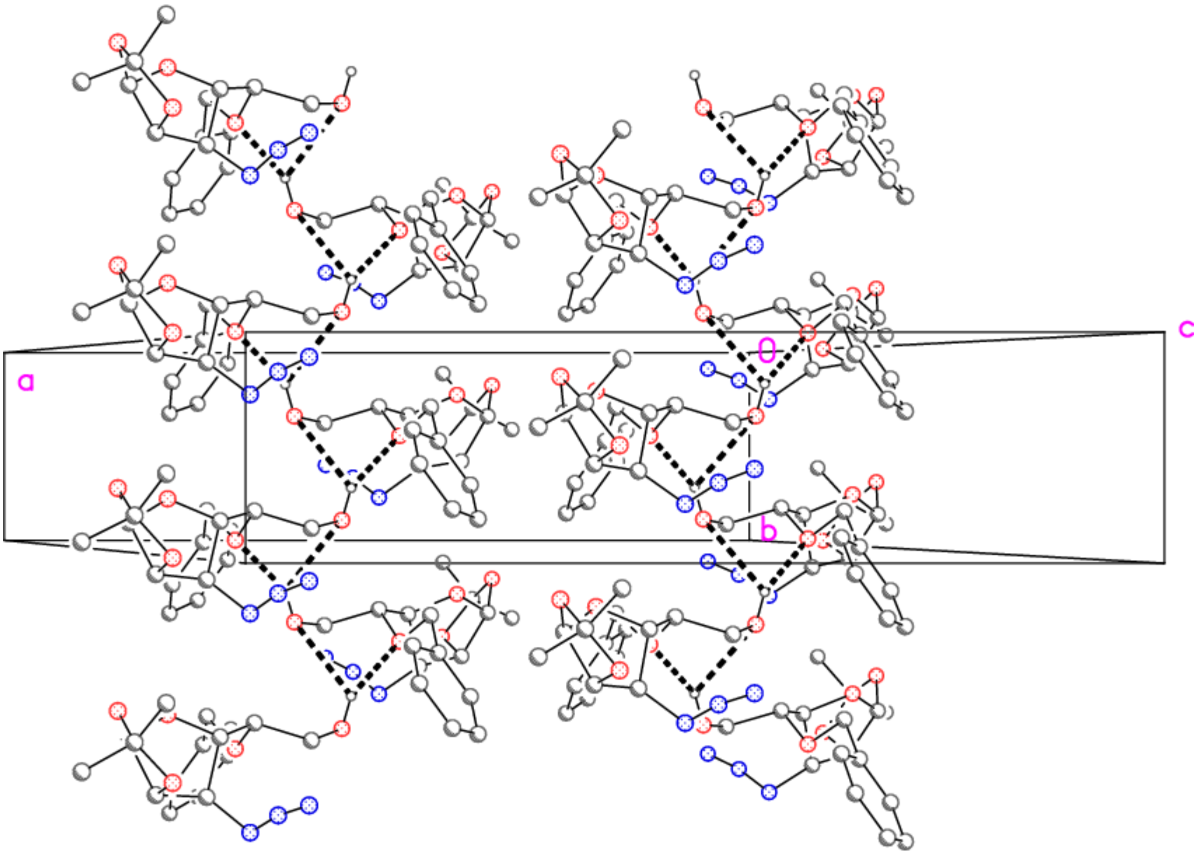

Figure 1.19: Packing diagram of compound **F** showing three-centre hydrogen bonds from H(06) to O5 and O6 of a neighbouring molecule, forming chains parallel to the *b* axis.

2 Quantification of the Interaction by QM Methods

2.1 Computational Details

All visualizations of calculated structures, molecular electrostatic potentials or dispersion interaction densities (DID) were created with UCSF Chimera[2] 1.10.2.

Quantum-mechanical calculations were performed with the ORCA 4.2.1[3] (DFT, MP2, DLPNO-CCSD(T)), MOLPRO2015.1[4] (W2-F12, W1-F12), TURBOMOLE 7.3.1[5],[6] (ESP) and xtb 6.2.1[7] (conformational search, GFN2-xTB) program packages. Møller-Plesset Perturbation Theory (SCS-MP2) applying the large def2-QZVPP basis set and Tight convergence criteria for energies and gradients as implemented in ORCA was used for geometry optimization of dimer and monomer structures. Opposite-spin and same-spin scaling parameters were chosen to $c_{OS} = 1.1$ and $c_{SS} = 2/3$, an improvement for gas phase structure optimization.[8] For SCS-MP2 the frozen core approximation was applied with default settings. *VeryTightPNO* settings were applied for the Local energy decomposition (LED) analyses in the DLPNO-CCSD(T)[9],[10] framework.[11] Default ECP-28[12] Stuttgart-Dresden effective core potentials (ECPs) were used for all elements with atomic numbers larger than Kr (Z = 36) in order to take into account scalar relativistic effects. The resolution-of-identity approximation for Coulomb and Exchange integrals (RIJK) was generally used with matching auxiliary basis sets to speed up the SCS-MP2 calculations, while the RI approximation for Coulomb integrals (RIJ) was applied to DFT calculations. The DFT-D4 dispersion correction scheme was applied for all non-composite DFT methods except ωB97X-V.

The CREST[13] program applying the iMTD-GC algorithm with the GFN2-xTB tight-binding semi-empirical method was used for automated conformer search. The obtained conformer rotamer ensemble was re-ranked with respect to Gibbs free energies with the ENSO[14],[15] script at PBEh-3c level of theory. Ro-vibrational were calculated with a modified rigid rotor harmonic oscillator statistical treatment[16] for T = 25.0 °C and 1 atm pressure based on harmonic frequency calculations at the geometry optimization level. Frequencies with wave numbers below 100 cm^{-1} were treated partially as rigid rotors to avoid errors in the harmonic approximation. Final conformational free energies were calculated at the DLPNO-CCSD(T)/CBS//SCS-MP2/def2-TZVPP level with *VeryTightPNO* settings for the lowest re-ranked conformers in a 2 kcal·mol^{-1} range.

Gas-phase Gibbs free energies were obtained by summing the gas phase single point energy E, the dispersion correction $E_{disp,D4}$, and the ro-vibrational correction G_{RRHO} (Eq. S1).

$$G_{tot} = E + E_{disp,D4} + G_{RRHO} \tag{S1}$$

DLPNO-CCSD(T) association energies and interaction energies in PES scans were basis set extrapolated by a two-point extrapolation scheme, applied to single point energies calculated with the def2-TZVPP and def2-QZVPP basis sets, extrapolation parameters for the SCF and the correlation energy, were set to 7.88 and 2.97, respectively as suggested by Neese and Valeev.[17] It is noteworthy that interaction energies obtained in the LED context are not extrapolated as LED contributions can not be extrapolated. Molecular electrostatic potentials with respect to a positively charged probe were calculated at PBE0/def2-SVP level. W2/W1-F12 calculations were conducted according to the published original protocol.[18]

2.2 Statistical measures

Statistical measure for a set $x_1, \cdots, x_n$ of data points with references $r_1, \cdots, r_n$ are :

- Mean deviation (MD):

$$\text{MD} = \frac{1}{n}\sum_{i}^{n}(x_i - r_i) \tag{S2}$$

- Mean absolute deviation (MAD):

$$\text{MAD} = \frac{1}{n}\sum_{i}^{n}(|x_i - r_i|) \tag{S3}$$

- Standard deviation (SD):

$$\text{SD} = \sqrt{\frac{1}{n-1}\sum_{i=1}^{n}\left((x_i - r_i) - \text{MD}\right)^2} \tag{S4}$$

- Absolute maximum deviation (AMAX):

$$\text{AMAX} = max\{|x_i - r_i|\} \tag{S5}$$

2.3 Local Energy Decomposition

Local Energy Decomposition analysis within the DLPNO-CCSD(T) framework is applied using the following partitioning of contributions (contributions in brackets are added):

$$\mathrm{E}_{int} = \mathrm{E}_{HF-elprep} + \mathrm{E}_{elstat} + \mathrm{E}_{exch} + \mathrm{E}_{C,elprep} + \mathrm{E}_{CT,XY}^{C-SP} + \mathrm{E}_{CT,YX}^{C-SP} + \mathrm{E}_{disp}^{C-SP} + \mathrm{E}^{C-WP} + \mathrm{E}^{(T)} \quad \text{(S6)}$$

$$\begin{aligned} &= \mathrm{E}_{0,HF-elprep} + \mathrm{E}_{0,elstat} + \mathrm{E}_{0,exch} + \left(\mathrm{E}_{HF-elprep}^{orb-relax} + \mathrm{E}_{elstat}^{orb-relax} + \mathrm{E}_{exch}^{orb-relax}\right) \\ &\quad + \left(\mathrm{E}_{C,elprep} + \mathrm{E}_{CT,XY}^{C-SP} + \mathrm{E}_{CT,YX}^{C-SP} + \mathrm{E}_{non-disp}^{(T)}\right) + \left(\mathrm{E}_{disp}^{C-SP} + \mathrm{E}^{C-WP} + \mathrm{E}_{disp}^{(T)}\right) \quad \text{(S7)} \\ &= \mathrm{E}_{0,HF-elprep} + \mathrm{E}_{0,elstat} + \mathrm{E}_{0,exch} + \mathrm{E}_{orb-relax} + \mathrm{E}_{non-disp} + \mathrm{E}_{disp} \quad \text{(S8)} \end{aligned}$$

Equation (S6) shows the standard LED partitioning, in eq. (S7) all HF contributions ($\mathrm{E}_{HFelprep}$, E_{elstat}, E_{exch}) are split into frozen contributions, denoted by a 0, and orbital relaxation contributions, according to ref. [19]. Furthermore, the $\mathrm{E}^{(T)}$ triples correction is partitioned into dispersive and non-dispersive triples corrections, according to ref. [20]. Expression (S8) displays the partitioning applied in this work.

2.4 Association Energies

Table S3: Association energies of systems **1-44** for various methods in kcal·mol^{-1}. Except for semi-empirical, composite and F12 methods the def2-QZVPP basis set was used.

#	GFN1-xTB	GFN2-xTB	PM6-D3H4X	PM7	HF-3c	B97-3c
1	−0.749	−0.348	−1.586	−1.099	−1.626	−1.097
2	−1.095	−0.881	−1.989	−1.362	−1.918	−1.265
3	−1.043	−0.894	−1.897	−1.455	−1.920	−1.427
4	−1.451	−1.500	−2.279	−1.507	−2.414	−1.874
5	−1.890	−1.657	−1.699	−4.083	−2.073	−1.620
6	−1.908	−1.787	−2.305	−4.028	−1.923	−1.616
7	−2.067	−1.717	−2.060	−3.630	−2.301	−1.673
8	−2.361	−1.844	−2.451	−4.035	−2.784	−2.043
9	−4.508	−4.537	2.748	−0.525	−5.210	−2.311
10	−7.545	−4.577	45.876	28.825	−7.708	−2.827
11	−0.873	−0.402	−1.889	−1.524	−2.009	−1.296
12	−1.371	−1.106	−2.384	−1.936	−2.729	−1.958
13	−0.888	−0.662	−1.764	−1.460	−2.096	−1.131
14	−1.445	−1.251	−2.628	−2.353	−3.048	−1.848
15	−6.088	−4.430	−9.317	−6.818	−10.051	−6.657
16	−2.881	−2.231	−3.985	−3.459	−4.985	−3.368
17	−1.813	−1.454	−3.811	−2.193	−4.275	−2.833
18	−2.979	−3.929	0.431	−0.796	−6.708	−3.227
19	−5.745	−4.379	43.660	26.783	−10.993	−4.048
20	−2.020	−1.779	−3.071	−3.438	−4.468	−2.929
21	−1.869	−1.539	−2.464	−1.994	−4.937	−3.071
22	−1.972	−1.658	−3.513	−4.167	−5.416	−3.340
23	−0.944	−1.206	−1.774	−1.980	−1.956	−1.480
24	−0.712	−1.086	−1.698	−1.635	−1.416	−1.445
25	−1.353	−1.207	−1.960	−1.700	−1.889	−1.582
26	−1.881	−1.734	−2.955	−2.621	−2.994	−2.168
27	−2.129	−1.849	−4.085	−2.888	−4.186	−3.035
28	−2.850	−3.974	−2.110	−1.867	−6.092	−3.298
29	−5.142	−4.318	29.814	17.948	−9.814	−3.907
30	−1.242	−1.254	−1.371	−1.152	−2.635	−1.814
31	−1.110	−0.936	−1.263	−1.027	−2.146	−1.475
32	−1.428	−1.615	−1.433	−1.129	−2.720	−1.696
33	−1.353	−3.266	1.776	1.130	−4.590	−1.790
34	−2.205	−3.489	−1.343	0.135	−7.284	−2.502
35	−0.833	−0.543	−1.270	−1.061	−1.096	−1.043
36	−1.253	−1.078	−1.680	−1.541	−1.841	−1.401
37	−1.008	−0.882	−1.448	−1.355	−1.641	−1.226
38	−1.337	−1.197	−1.825	−4.134	−2.148	−1.670
39	−1.639	−1.708	−1.907	−4.169	−2.388	−1.935
40	−1.393	−1.525	−1.747	−4.020	−2.338	−1.790
41	−1.364	−1.199	−0.875	−2.648	−3.209	−1.838
42	−1.993	−1.503	−0.949	−2.814	−3.359	−2.136
43	−1.324	−1.290	−1.331	−2.616	−3.412	−2.065
44	−2.012	−1.679	−1.328	−2.615	−3.361	−2.402

Table S4: Association energies of systems **1-44** for various methods in kcal·mol^{-1}. Except for semi-empirical, composite and F12 methods the def2-QZVPP basis set was used.

#	PBEh-3c	TPSS-D4	PBE-D4	B3LYP-D4	PBE0-D4	ωB97X-V	PWPB95
1	−2.229	−1.529	−1.814	−1.894	−1.876	−2.149	−1.908
2	−2.466	−1.589	−1.940	−1.848	−1.939	−2.129	−1.778
3	−2.719	−1.738	−2.026	−2.098	−2.074	−2.327	−2.076
4	−2.896	−2.067	−2.387	−2.421	−2.373	−2.798	−2.667
5	−2.268	−1.792	−1.959	−1.948	−1.887	−1.837	−1.782
6	−2.256	−1.720	−1.990	−1.793	−1.882	−1.847	−1.638
7	−2.465	−1.783	−1.984	−1.947	−1.938	−1.955	−1.866
8	−3.022	−2.091	−2.292	−2.229	−2.303	−2.479	−2.215
9	−2.866	−2.254	−2.397	−2.408	−2.378	−2.478	−2.230
10	−3.269	−2.690	−2.731	−2.781	−2.700	−2.776	−2.416
11	−2.222	−1.585	−1.857	−1.999	−1.953	−2.303	−1.975
12	−2.857	−2.202	−2.420	−2.494	−2.415	−2.647	−2.367
13	−2.153	−1.449	−1.751	−1.775	−1.821	−2.076	−1.654
14	−3.136	−2.191	−2.325	−2.701	−2.522	−3.084	−2.745
15	−8.451	−7.120	−7.263	−7.550	−7.649	−7.818	−6.976
16	−4.929	−3.465	−3.683	−4.091	−3.965	−4.383	−3.911
17	−4.443	−2.885	−3.120	−3.554	−3.418	−3.894	−3.364
18	−4.790	−3.348	−3.523	−4.134	−3.930	−4.493	−3.893
19	−5.988	−4.253	−4.150	−5.022	−4.789	−5.491	−4.771
20	−4.371	−2.911	−3.180	−3.469	−3.421	−3.837	−3.310
21	−4.552	−3.216	−3.497	−3.904	−3.808	−4.242	−3.686
22	−4.818	−3.331	−3.609	−4.087	−3.959	−4.353	−3.765
23	−2.408	−1.818	−2.038	−2.176	−2.058	−2.291	−2.166
24	−2.129	−1.603	−1.934	−2.031	−1.915	−2.252	−1.962
25	−2.440	−1.721	−2.007	−1.857	−1.940	−1.941	−1.646
26	−3.236	−2.382	−2.511	−2.673	−2.527	−2.771	−2.577
27	−4.445	−3.025	−3.254	−3.411	−3.385	−3.606	−3.137
28	−4.665	−3.370	−3.544	−3.818	−3.709	−3.946	−3.441
29	−5.360	−4.012	−3.962	−4.472	−4.233	−4.556	−4.049
30	−2.755	−1.948	−2.117	−2.208	−2.071	−2.121	−2.066
31	−2.266	−1.689	−1.925	−1.959	−1.877	−2.001	−1.856
32	−2.312	−1.849	−2.121	−1.981	−2.023	−1.962	−1.659
33	−2.223	−1.881	−2.069	−2.145	−1.997	−2.054	−1.879
34	−2.718	−2.358	−2.477	−2.534	−2.372	−2.403	−2.163
35	−1.627	−1.378	−1.624	−1.662	−1.613	−1.928	−1.674
36	−2.142	−1.724	−1.965	−1.924	−1.949	−2.085	−1.827
37	−1.830	−1.583	−1.808	−1.764	−1.769	−1.874	−1.620
38	−2.434	−1.819	−2.035	−2.086	−2.034	−2.181	−1.880
39	−2.776	−2.029	−2.305	−2.231	−2.230	−2.256	−1.997
40	−2.552	−1.954	−2.215	−2.165	−2.132	−2.128	−1.851
41	−2.327	−2.033	−2.210	−2.296	−2.199	−2.268	−2.024
42	−2.708	−2.266	−2.509	−2.486	−2.404	−2.330	−2.099
43	−2.307	−2.211	−2.354	−2.439	−2.330	−2.296	−2.017
44	−2.684	−2.470	−2.684	−2.650	−2.541	−2.381	−2.181

Table S5: Association energies of systems **1-44** for various methods in kcal·mol^{-1}. Except for semi-empirical, composite and F12 methods the def2-QZVPP basis set was used.

#	B2PLYP	SCS-MP2	DLPNO-CCSD(T)/CBS	W1/W2-F12	W1 or W2?
1	−1.862	−1.919	−2.079	−2.226	W2
2	−1.868	−1.877	−2.055	−2.218	W2
3	−2.188	−2.329	−2.311	−2.344	W1
4	−2.585	−2.946	−2.820	−2.767	W1
5	−1.892	−1.989	−1.960	−2.105	W2
6	−1.836	−1.943	−1.928	−2.083	W2
7	−2.003	−2.203	−2.099	−2.040	W1
8	−2.305	−2.549	−2.488	−2.441	W1
9	−2.426	−2.705	−2.517		
10	−2.813	−3.197	−2.958		
11	−1.948	−1.907	−2.200	−2.375	W2
12	−2.431	−2.422	−2.622	−2.712	W2
13	−1.752	−1.698	−1.961	−2.165	W2
14	−2.763	−3.018	−3.035	−3.237	W2
15	−7.265	−7.006	−7.602	−7.708	W2
16	−4.002	−4.015	−4.308	−4.535	W2
17	−3.429	−3.470	−3.768	−3.779	W1
18	−3.957	−4.067	−4.330		
19	−4.773	−5.084	−5.453		
20	−3.426	−3.463	−3.786	−3.992	W2
21	−3.858	−3.973	−4.284		
22	−4.034	−4.208	−4.515		
23	−2.264	−2.360	−2.359	−2.528	W2
24	−2.085	−2.176	−2.237	−2.482	W2
25	−1.859	−1.872	−1.971	−2.135	W2
26	−2.822	−3.194	−2.883	−3.131	W2
27	−3.396	−3.589	−3.584	−3.598	W1
28	−3.740	−3.956	−3.920		
29	−4.340	−4.728	−4.643		
30	−2.269	−2.474	−2.341	−2.469	W2
31	−1.979	−2.178	−2.158	−2.342	W2
32	−1.958	−2.028	−2.028		
33	−2.116	−2.382	−2.181		
34	−2.575	−3.106	−2.963		
35	−1.618	−1.547	−1.790	−2.000	W2
36	−1.985	−1.982	−2.092	−2.079	W1
37	−1.731	−1.631	−1.847	−2.024	W2
38	−2.046	−2.101	−2.202	−2.444	W2
39	−2.316	−2.419	−2.383	−2.379	W1
40	−2.156	−2.158	−2.239	−2.432	W2
41	−2.217	−2.324	−2.354		
42	−2.492	−2.613	−2.464		
43	−2.365	−2.568	−2.499		
44	−2.652	−2.874	−2.659		

2.5 Potential Energy Surface Scans

Table S6: Interaction energies of the potential energy surface scan of system **1** for various methods at N $\cdots$ N2 distances between 1.8 and 6.0 Å, energies in kcal·mol^{-1}. DLPNO-CCSD(T)/CBS is abbreviated as DLPNO/CBS.

Dist.	PBEh-3c	PBE0	B3LYP	ωB97X-V	B2PLYP	SCS-MP2	DLPNO/CBS	W2-F12
1.8	121.473	115.754	121.225	122.645	118.944	115.345	119.085	117.984
1.9	86.917	83.182	87.495	88.312	85.262	81.663	84.949	84.095
2.0	60.701	58.503	61.798	62.244	59.839	56.766	59.164	58.573
2.1	41.324	40.251	42.729	42.877	41.060	38.557	40.282	39.769
2.2	27.269	26.983	28.805	28.747	27.415	25.426	26.534	26.172
2.3	17.249	17.485	18.780	18.606	17.654	16.108	16.784	16.512
2.4	10.236	10.793	11.671	11.453	10.785	9.611	9.952	9.775
2.5	5.428	6.157	6.716	6.486	6.042	5.174	5.310	5.172
2.6	2.215	3.010	3.331	3.116	2.839	2.219	2.228	2.106
2.7	0.140	0.926	1.079	0.894	0.737	0.314	0.266	0.132
2.8	−1.133	−0.409	−0.364	−0.525	−0.586	−0.859	−0.932	−1.080
2.9	−1.852	−1.223	−1.239	−1.380	−1.369	−1.530	−1.585	−1.768
3.0	−2.195	−1.676	−1.723	−1.841	−1.785	−1.865	−1.939	−2.103
3.1	−2.291	−1.891	−1.940	−2.046	−1.954	−1.980	−2.040	−2.208
3.2	−2.230	−1.949	−1.979	−2.082	−1.964	−1.956	−2.009	−2.167
3.3	−2.076	−1.902	−1.911	−2.005	−1.877	−1.850	−1.889	−2.040
3.4	−1.871	−1.793	−1.776	−1.866	−1.732	−1.697	−1.739	−1.865
3.5	−1.647	−1.653	−1.604	−1.696	−1.558	−1.524	−1.561	−1.670
3.6	−1.420	−1.495	−1.420	−1.510	−1.375	−1.345	−1.374	−1.471
3.7	−1.202	−1.330	−1.238	−1.322	−1.195	−1.170	−1.197	−1.279
3.8	−1.001	−1.171	−1.063	−1.144	−1.024	−1.006	−1.029	−1.100
3.9	−0.821	−1.020	−0.901	−0.979	−0.866	−0.856	−0.875	−0.935
4.0	−0.661	−0.880	−0.756	−0.828	−0.724	−0.720	−0.736	−0.788
4.1	−0.520	−0.750	−0.628	−0.691	−0.599	−0.599	−0.612	−0.657
4.2	−0.399	−0.634	−0.516	−0.571	−0.489	−0.492	−0.501	−0.542
4.3	−0.296	−0.530	−0.418	−0.467	−0.393	−0.398	−0.411	−0.441
4.4	−0.211	−0.439	−0.334	−0.376	−0.311	−0.317	−0.327	−0.354
4.5	−0.139	−0.357	−0.263	−0.296	−0.241	−0.246	−0.259	−0.279
4.6	−0.079	−0.286	−0.203	−0.228	−0.181	−0.185	−0.203	−0.214
4.7	−0.031	−0.224	−0.152	−0.170	−0.130	−0.132	−0.149	−0.159
4.8	0.009	−0.171	−0.109	−0.121	−0.087	−0.087	−0.101	−0.111
4.9	0.040	−0.125	−0.072	−0.079	−0.051	−0.048	−0.065	−0.071
5.0	0.066	−0.085	−0.042	−0.042	−0.020	−0.015	−0.032	−0.037
5.1	0.086	−0.050	−0.017	−0.012	0.005	0.013	−0.003	−0.008
5.2	0.102	−0.021	0.005	0.014	0.027	0.037	0.019	0.017
5.3	0.115	0.004	0.024	0.035	0.046	0.057	0.041	0.037
5.4	0.125	0.025	0.039	0.054	0.061	0.075	0.061	0.054
5.5	0.133	0.043	0.052	0.069	0.074	0.089	0.076	0.069
5.6	0.140	0.058	0.063	0.082	0.084	0.101	0.090	0.081
5.7	0.144	0.070	0.072	0.093	0.093	0.111	0.101	0.090
5.8	0.148	0.081	0.080	0.101	0.101	0.119	0.108	0.098
5.9	0.150	0.089	0.086	0.108	0.107	0.125	0.115	0.105
6.0	0.152	0.096	0.091	0.114	0.111	0.131	0.119	0.110

Table S7: Interaction energies of the potential energy surface scan of system **11** for various methods at O$\cdots$N2 distances between 1.8 and 6.0 Å, energies in kcal·mol^{-1}. DLPNO-CCSD(T)/CBS is abbreviated as DLPNO/CBS.

Dist.	PBEh-3c	PBE0	B3LYP	ωB97X-V	B2PLYP	SCS-MP2	DLPNO/CBS	W2-F12
1.8	101.134	95.578	100.405	101.446	98.906	96.586	98.875	97.866
1.9	70.829	66.894	70.593	71.242	69.176	67.053	68.726	68.012
2.0	48.511	45.866	48.645	48.958	47.381	45.626	46.622	46.134
2.1	32.344	30.703	32.747	32.789	31.651	30.256	30.851	30.384
2.2	20.802	19.919	21.376	21.236	20.454	19.384	19.590	19.236
2.3	12.695	12.358	13.352	13.112	12.606	11.814	11.715	11.482
2.4	7.104	7.140	7.778	7.496	7.197	6.641	6.396	6.196
2.5	3.331	3.604	3.979	3.689	3.548	3.181	2.872	2.676
2.6	0.857	1.263	1.449	1.175	1.148	0.933	0.599	0.405
2.7	−0.703	−0.240	−0.180	−0.428	−0.373	−0.474	−0.791	−0.997
2.8	−1.626	−1.164	−1.179	−1.400	−1.286	−1.304	−1.602	−1.804
2.9	−2.115	−1.692	−1.743	−1.940	−1.786	−1.748	−2.007	−2.214
3.0	−2.314	−1.954	−2.014	−2.188	−2.011	−1.937	−2.158	−2.362
3.1	−2.326	−2.040	−2.090	−2.247	−2.057	−1.965	−2.156	−2.347
3.2	−2.222	−2.016	−2.042	−2.189	−1.991	−1.893	−2.059	−2.233
3.3	−2.052	−1.923	−1.921	−2.057	−1.860	−1.764	−1.915	−2.065
3.4	−1.849	−1.789	−1.760	−1.886	−1.696	−1.608	−1.735	−1.872
3.5	−1.635	−1.637	−1.581	−1.702	−1.518	−1.441	−1.546	−1.672
3.6	−1.427	−1.479	−1.398	−1.515	−1.339	−1.275	−1.378	−1.478
3.7	−1.230	−1.320	−1.224	−1.333	−1.170	−1.117	−1.209	−1.295
3.8	−1.048	−1.168	−1.062	−1.164	−1.013	−0.972	−1.053	−1.128
3.9	−0.886	−1.028	−0.913	−1.010	−0.870	−0.839	−0.913	−0.977
4.0	−0.744	−0.898	−0.781	−0.871	−0.742	−0.720	−0.788	−0.842
4.1	−0.618	−0.779	−0.666	−0.746	−0.630	−0.614	−0.672	−0.724
4.2	−0.510	−0.672	−0.565	−0.637	−0.533	−0.521	−0.572	−0.619
4.3	−0.418	−0.578	−0.477	−0.542	−0.447	−0.439	−0.485	−0.528
4.4	−0.340	−0.494	−0.401	−0.460	−0.373	−0.368	−0.411	−0.449
4.5	−0.274	−0.419	−0.337	−0.387	−0.311	−0.306	−0.351	−0.380
4.6	−0.218	−0.354	−0.282	−0.324	−0.257	−0.252	−0.300	−0.321
4.7	−0.172	−0.297	−0.235	−0.270	−0.210	−0.205	−0.251	−0.269
4.8	−0.134	−0.247	−0.193	−0.224	−0.170	−0.164	−0.204	−0.225
4.9	−0.102	−0.204	−0.159	−0.184	−0.136	−0.129	−0.165	−0.187
5.0	−0.075	−0.166	−0.130	−0.148	−0.108	−0.098	−0.139	−0.154
5.1	−0.053	−0.133	−0.104	−0.118	−0.083	−0.072	−0.111	−0.125
5.2	−0.034	−0.105	−0.082	−0.092	−0.061	−0.049	−0.085	−0.101
5.3	−0.019	−0.080	−0.062	−0.070	−0.042	−0.029	−0.053	−0.080
5.4	−0.005	−0.059	−0.046	−0.051	−0.026	−0.012	−0.035	−0.062
5.5	0.006	−0.041	−0.032	−0.033	−0.012	0.002	−0.019	−0.046
5.6	0.015	−0.025	−0.019	−0.019	0.000	0.015	−0.005	−0.033
5.7	0.024	−0.011	−0.008	−0.006	0.011	0.026	0.006	−0.021
5.8	0.030	0.000	0.002	0.004	0.020	0.035	0.016	−0.012
5.9	0.036	0.010	0.010	0.014	0.028	0.043	0.024	−0.003
6.0	0.041	0.019	0.017	0.022	0.034	0.049	0.030	0.004

Table S8: Interaction energies of the potential energy surface scan of system **35** for various methods at F $\cdots$ N2 distances between 1.8 and 6.0 Å, energies in kcal·mol^{-1}. DLPNO-CCSD(T)/CBS is abbreviated as DLPNO/CBS.

Dist.	PBEh-3c	PBE0	B3LYP	ωB97X-V	B2PLYP	SCS-MP2	DLPNO/CBS	W2-F12
1.8	76.673	73.783	77.116	77.236	75.377	73.015	74.221	73.645
1.9	52.607	50.561	53.014	52.958	51.502	49.656	50.435	49.825
2.0	35.375	33.966	35.723	35.530	34.445	33.050	33.355	32.877
2.1	23.170	22.257	23.472	23.190	22.417	21.392	21.334	20.994
2.2	14.623	14.094	14.894	14.566	14.046	13.320	13.069	12.789
2.3	8.718	8.478	8.966	8.631	8.306	7.817	7.478	7.219
2.4	4.708	4.670	4.937	4.610	4.440	4.134	3.771	3.515
2.5	2.049	2.140	2.249	1.952	1.893	1.726	1.365	1.116
2.6	0.342	0.495	0.508	0.240	0.266	0.200	−0.125	−0.382
2.7	−0.705	−0.538	−0.576	−0.819	−0.728	−0.724	−1.006	−1.267
2.8	−1.301	−1.151	−1.213	−1.424	−1.295	−1.245	−1.483	−1.743
2.9	−1.597	−1.485	−1.545	−1.733	−1.576	−1.499	−1.710	−1.950
3.0	−1.698	−1.636	−1.676	−1.847	−1.671	−1.583	−1.757	−1.988
3.1	−1.676	−1.662	−1.682	−1.832	−1.653	−1.561	−1.705	−1.923
3.2	−1.581	−1.615	−1.608	−1.744	−1.564	−1.477	−1.600	−1.799
3.3	−1.446	−1.525	−1.488	−1.615	−1.437	−1.360	−1.466	−1.646
3.4	−1.293	−1.409	−1.348	−1.465	−1.294	−1.228	−1.317	−1.482
3.5	−1.136	−1.280	−1.203	−1.307	−1.149	−1.094	−1.178	−1.320
3.6	−0.983	−1.151	−1.059	−1.154	−1.007	−0.963	−1.038	−1.166
3.7	−0.840	−1.025	−0.923	−1.012	−0.874	−0.841	−0.908	−1.024
3.8	−0.711	−0.905	−0.800	−0.879	−0.754	−0.729	−0.789	−0.895
3.9	−0.596	−0.794	−0.690	−0.758	−0.647	−0.628	−0.684	−0.780
4.0	−0.494	−0.692	−0.592	−0.652	−0.551	−0.537	−0.586	−0.678
4.1	−0.405	−0.600	−0.507	−0.558	−0.468	−0.457	−0.501	−0.588
4.2	−0.329	−0.518	−0.432	−0.476	−0.395	−0.386	−0.425	−0.510
4.3	−0.265	−0.445	−0.369	−0.404	−0.333	−0.324	−0.362	−0.441
4.4	−0.210	−0.381	−0.314	−0.341	−0.279	−0.270	−0.307	−0.382
4.5	−0.165	−0.323	−0.267	−0.286	−0.233	−0.223	−0.258	−0.331
4.6	−0.126	−0.273	−0.226	−0.240	−0.193	−0.182	−0.215	−0.287
4.7	−0.095	−0.230	−0.190	−0.199	−0.158	−0.146	−0.177	−0.248
4.8	−0.068	−0.191	−0.160	−0.163	−0.129	−0.115	−0.144	−0.216
4.9	−0.046	−0.158	−0.134	−0.132	−0.103	−0.088	−0.118	−0.187
5.0	−0.028	−0.128	−0.111	−0.106	−0.081	−0.065	−0.093	−0.163
5.1	−0.013	−0.103	−0.091	−0.083	−0.061	−0.045	−0.071	−0.142
5.2	0.000	−0.081	−0.074	−0.063	−0.044	−0.027	−0.049	−0.124
5.3	0.010	−0.062	−0.059	−0.045	−0.030	−0.012	−0.030	−0.109
5.4	0.019	−0.046	−0.046	−0.030	−0.018	0.001	−0.016	−0.095
5.5	0.027	−0.032	−0.034	−0.017	−0.007	0.012	−0.004	−0.084
5.6	0.033	−0.019	−0.024	−0.006	0.003	0.022	0.005	−0.074
5.7	0.039	−0.009	−0.015	0.003	0.011	0.030	0.011	−0.066
5.8	0.043	0.000	−0.008	0.011	0.018	0.037	0.017	−0.059
5.9	0.047	0.007	−0.002	0.018	0.024	0.043	0.024	−0.053
6.0	0.050	0.013	0.003	0.024	0.029	0.047	0.030	−0.048

2.6 Local Energy Decomposition Scans

Table S9: Local Energy Decomposition analysis contributions to the DLPNO-CCSD(T) interaction energy of system **1** at N···N2 distances between 1.8 and 6.0 Å, energies in kcal·mol^{-1}.

Dist.	$E_{0,HF-elprep}$	$E_{0,elstat}$	$E_{0,exch}$	$E_{orb-relax}$	E_{disp}	$E_{non-disp}$	E_{int}
1.8	605.591	−296.007	−103.980	−57.631	−19.840	−8.207	119.925
1.9	466.165	−236.025	−83.162	−38.088	−17.004	−6.272	85.615
2.0	357.703	−187.006	−66.199	−25.653	−14.374	−4.646	59.825
2.1	273.607	−147.311	−52.445	−17.545	−12.276	−3.199	40.832
2.2	208.609	−115.417	−41.348	−12.159	−10.497	−2.142	27.046
2.3	158.529	−89.970	−32.439	−8.530	−8.934	−1.419	17.237
2.4	120.066	−69.800	−25.324	−6.056	−7.684	−0.844	10.358
2.5	90.625	−53.911	−19.672	−4.350	−6.490	−0.557	5.645
2.6	68.169	−41.466	−15.206	−3.162	−5.555	−0.278	2.502
2.7	51.100	−31.772	−11.696	−2.327	−4.798	−0.026	0.480
2.8	38.175	−24.259	−8.953	−1.734	−4.116	0.115	−0.773
2.9	28.422	−18.462	−6.822	−1.310	−3.573	0.264	−1.480
3.0	21.090	−14.009	−5.174	−1.003	−3.056	0.290	−1.860
3.1	15.598	−10.599	−3.906	−0.778	−2.574	0.264	−1.996
3.2	11.497	−7.997	−2.937	−0.613	−2.223	0.285	−1.987
3.3	8.447	−6.017	−2.199	−0.489	−1.905	0.276	−1.887
3.4	6.184	−4.512	−1.639	−0.396	−1.645	0.270	−1.738
3.5	4.512	−3.372	−1.217	−0.324	−1.441	0.279	−1.563
3.6	3.281	−2.508	−0.900	−0.269	−1.244	0.260	−1.381
3.7	2.376	−1.854	−0.663	−0.226	−1.082	0.242	−1.206
3.8	1.715	−1.360	−0.486	−0.191	−0.933	0.217	−1.039
3.9	1.233	−0.987	−0.355	−0.163	−0.805	0.193	−0.885
4.0	0.882	−0.705	−0.258	−0.141	−0.715	0.191	−0.745
4.1	0.629	−0.491	−0.187	−0.123	−0.657	0.208	−0.620
4.2	0.447	−0.330	−0.135	−0.107	−0.573	0.187	−0.510
4.3	0.316	−0.207	−0.097	−0.094	−0.501	0.167	−0.417
4.4	0.222	−0.114	−0.069	−0.083	−0.442	0.152	−0.333
4.5	0.156	−0.043	−0.049	−0.074	−0.394	0.141	−0.263
4.6	0.109	0.012	−0.035	−0.066	−0.347	0.124	−0.203
4.7	0.075	0.053	−0.024	−0.059	−0.306	0.112	−0.149
4.8	0.052	0.085	−0.017	−0.053	−0.270	0.101	−0.102
4.9	0.036	0.109	−0.012	−0.048	−0.240	0.090	−0.064
5.0	0.025	0.128	−0.008	−0.043	−0.214	0.083	−0.030
5.1	0.017	0.142	−0.006	−0.039	−0.191	0.077	−0.000
5.2	0.011	0.152	−0.004	−0.035	−0.171	0.069	0.023
5.3	0.008	0.160	−0.003	−0.031	−0.152	0.062	0.044
5.4	0.005	0.166	−0.002	−0.028	−0.137	0.058	0.063
5.5	0.003	0.170	−0.001	−0.025	−0.122	0.053	0.077
5.6	0.002	0.173	−0.001	−0.023	−0.110	0.048	0.090
5.7	0.002	0.174	−0.001	−0.020	−0.099	0.044	0.100
5.8	0.001	0.175	−0.000	−0.018	−0.089	0.039	0.107
5.9	0.001	0.175	−0.000	−0.016	−0.081	0.034	0.113
6.0	0.000	0.174	−0.000	−0.014	−0.074	0.031	0.117

Table S10: Local Energy Decomposition analysis contributions to the DLPNO-CCSD(T) interaction energy of system **11** at O···N2 distances between 1.8 and 6.0 Å, energies in kcal·mol^{-1}.

Dist.	$E_{0,HF-elprep}$	$E_{0,elstat}$	$E_{0,exch}$	$E_{orb-relax}$	E_{disp}	$E_{non-disp}$	E_{int}
1.8	640.594	−355.816	−110.627	−50.988	−18.059	−5.387	99.718
1.9	486.204	−276.396	−87.234	−33.950	−15.150	−3.995	69.478
2.0	367.687	−213.554	−68.396	−22.929	−12.848	−2.627	47.332
2.1	277.059	−164.187	−53.326	−15.683	−10.776	−1.637	31.450
2.2	208.019	−125.661	−41.348	−10.853	−9.152	−0.889	20.115
2.3	155.624	−95.778	−31.889	−7.597	−7.827	−0.362	12.171
2.4	116.010	−72.728	−24.464	−5.380	−6.642	−0.027	6.769
2.5	86.175	−55.044	−18.673	−3.855	−5.684	0.255	3.175
2.6	63.793	−41.545	−14.181	−2.796	−4.810	0.381	0.841
2.7	47.066	−31.288	−10.718	−2.054	−4.078	0.465	−0.606
2.8	34.613	−23.525	−8.063	−1.530	−3.486	0.529	−1.462
2.9	25.374	−17.670	−6.038	−1.156	−2.937	0.524	−1.903
3.0	18.544	−13.266	−4.503	−0.886	−2.497	0.515	−2.093
3.1	13.511	−9.962	−3.344	−0.689	−2.180	0.548	−2.115
3.2	9.814	−7.486	−2.473	−0.544	−1.861	0.515	−2.035
3.3	7.107	−5.633	−1.822	−0.436	−1.599	0.486	−1.897
3.4	5.131	−4.248	−1.337	−0.354	−1.378	0.458	−1.728
3.5	3.692	−3.211	−0.977	−0.292	−1.175	0.414	−1.549
3.6	2.649	−2.435	−0.712	−0.243	−1.000	0.366	−1.376
3.7	1.894	−1.854	−0.516	−0.205	−0.878	0.351	−1.208
3.8	1.350	−1.417	−0.373	−0.175	−0.765	0.327	−1.053
3.9	0.959	−1.088	−0.269	−0.151	−0.667	0.303	−0.913
4.0	0.679	−0.839	−0.193	−0.131	−0.608	0.305	−0.787
4.1	0.479	−0.650	−0.138	−0.114	−0.530	0.279	−0.673
4.2	0.337	−0.505	−0.098	−0.101	−0.470	0.262	−0.575
4.3	0.236	−0.393	−0.070	−0.089	−0.419	0.246	−0.489
4.4	0.165	−0.306	−0.049	−0.079	−0.367	0.223	−0.414
4.5	0.115	−0.238	−0.035	−0.070	−0.322	0.202	−0.348
4.6	0.080	−0.185	−0.024	−0.063	−0.283	0.180	−0.295
4.7	0.055	−0.142	−0.017	−0.056	−0.250	0.164	−0.245
4.8	0.038	−0.107	−0.012	−0.050	−0.223	0.154	−0.200
4.9	0.026	−0.079	−0.008	−0.045	−0.199	0.144	−0.161
5.0	0.018	−0.056	−0.006	−0.041	−0.176	0.131	−0.129
5.1	0.012	−0.036	−0.004	−0.037	−0.157	0.120	−0.101
5.2	0.008	−0.020	−0.003	−0.033	−0.140	0.110	−0.077
5.3	0.006	−0.007	−0.002	−0.029	−0.124	0.108	−0.049
5.4	0.004	0.004	−0.001	−0.026	−0.112	0.099	−0.032
5.5	0.003	0.014	−0.001	−0.024	−0.100	0.092	−0.016
5.6	0.002	0.022	−0.001	−0.021	−0.091	0.085	−0.004
5.7	0.001	0.029	−0.000	−0.019	−0.082	0.078	0.007
5.8	0.001	0.035	−0.000	−0.017	−0.074	0.072	0.016
5.9	0.001	0.040	−0.000	−0.015	−0.067	0.065	0.023
6.0	0.000	0.044	−0.000	−0.013	−0.062	0.060	0.029

Table S11: Local Energy Decomposition analysis contributions to the DLPNO-CCSD(T) interaction energy of system **35** at F···N2 distances between 1.8 and 6.0 Å, energies in kcal·mol^{-1}.

Dist.	$E_{0,HF-elprep}$	$E_{0,elstat}$	$E_{0,exch}$	$E_{orb-relax}$	E_{disp}	$E_{non-disp}$	E_{int}
1.8	550.620	−333.458	-91.957	−30.769	−14.396	−5.024	75.016
1.9	412.676	−253.845	−71.425	−20.657	−12.141	−3.558	51.050
2.0	308.224	−192.368	−55.170	−14.029	−10.216	−2.530	33.911
2.1	229.434	−145.175	−42.388	−9.630	−8.694	−1.715	21.832
2.2	170.213	−109.140	−32.401	−6.678	−7.353	−1.141	13.501
2.3	125.856	−81.757	−24.644	−4.679	−6.309	−0.631	7.835
2.4	92.754	−61.051	−18.653	−3.313	−5.292	−0.388	4.056
2.5	68.131	−45.456	−14.051	−2.373	−4.504	−0.159	1.589
2.6	49.882	−33.762	−10.534	−1.720	−3.853	0.023	0.037
2.7	36.404	−25.024	−7.860	−1.264	−3.270	0.126	−0.889
2.8	26.484	−18.519	−5.839	−0.942	−2.768	0.180	−1.403
2.9	19.208	−13.690	−4.318	−0.713	−2.362	0.217	−1.659
3.0	13.889	−10.115	−3.180	−0.548	−2.003	0.228	−1.729
3.1	10.013	−7.473	−2.332	−0.428	−1.703	0.230	−1.694
3.2	7.197	−5.524	−1.704	−0.340	−1.466	0.238	−1.599
3.3	5.158	−4.087	−1.240	−0.275	−1.240	0.215	−1.470
3.4	3.685	−3.030	−0.899	−0.225	−1.056	0.198	−1.326
3.5	2.626	−2.250	−0.649	−0.187	−0.928	0.207	−1.183
3.6	1.865	−1.676	−0.468	−0.158	−0.800	0.195	−1.042
3.7	1.321	−1.251	−0.336	−0.135	−0.704	0.194	−0.911
3.8	0.933	−0.937	−0.240	−0.116	−0.610	0.179	−0.791
3.9	0.657	−0.704	−0.171	−0.101	−0.535	0.171	−0.683
4.0	0.461	−0.530	−0.122	−0.088	−0.479	0.173	−0.584
4.1	0.323	−0.400	−0.086	−0.078	−0.422	0.163	−0.499
4.2	0.226	−0.301	−0.061	−0.069	−0.370	0.153	−0.423
4.3	0.157	−0.226	−0.043	−0.062	−0.328	0.143	−0.359
4.4	0.109	−0.169	−0.030	−0.055	−0.292	0.137	−0.301
4.5	0.075	−0.124	−0.021	−0.049	−0.255	0.124	−0.251
4.6	0.052	−0.090	−0.015	−0.044	−0.226	0.114	−0.208
4.7	0.036	−0.062	−0.010	−0.040	−0.200	0.106	−0.170
4.8	0.025	−0.040	−0.007	−0.036	−0.177	0.098	−0.137
4.9	0.017	−0.022	−0.005	−0.032	−0.158	0.091	−0.109
5.0	0.011	−0.008	−0.003	−0.028	−0.141	0.084	−0.085
5.1	0.008	0.004	−0.002	−0.025	−0.126	0.078	−0.063
5.2	0.005	0.013	−0.002	−0.022	−0.113	0.075	−0.043
5.3	0.004	0.021	−0.001	−0.020	−0.101	0.070	−0.027
5.4	0.002	0.028	−0.001	−0.018	−0.091	0.065	−0.014
5.5	0.002	0.034	−0.001	−0.016	−0.082	0.060	−0.003
5.6	0.001	0.038	−0.000	−0.014	−0.074	0.054	0.005
5.7	0.001	0.042	−0.000	−0.012	−0.066	0.048	0.012
5.8	0.000	0.045	−0.000	−0.011	−0.061	0.044	0.018
5.9	0.000	0.048	−0.000	−0.009	−0.055	0.041	0.025
6.0	0.000	0.050	−0.000	−0.008	−0.050	0.039	0.030

2.7 NBO SOPT Hydrogen Bonding Estimate

Table S12: Dimer interaction energies for all systems at PBE0-D4/def2-QZVPP and DLPNO-CCSD(T)/def2-QZVPP level of theory, NBO SOPT estimate of the hydrogen bonding contribution to the interaction energy at the PBE0-D4/def2-QZVPP level and a scaled estimate $SOPT_{scaled}$(PBE0-D4), the scaling factor results from the ratio of E_{int}(LED) to E_{int}(PBE0-D4), the last line gives the mean values, all values in kcal·mol^{-1}.

#	E_{int}(PBE0-D4)	E_{int}(LED)	E_{SOPT}(PBE0-D4)	Scalingfactor	E_{SOPT}^{scaled}(PBE0-D4)
1	−1.92	−2.00	−0.13	1.04	−0.14
2	−1.93	−1.98	−0.24	1.03	−0.25
3	−2.10	−2.28	−0.09	1.09	−0.10
4	−2.35	−2.81	0.00	1.20	0.00
5	−1.91	−1.84	−0.10	0.97	−0.10
6	−1.87	−1.82	−0.22	0.97	−0.21
7	−1.96	−2.01	0.00	1.03	0.00
8	−2.33	−2.44	0.00	1.05	0.00
9	−2.42	−2.53	0.00	1.05	0.00
10	−2.74	−2.92	0.00	1.07	0.00
11	−2.03	−2.12	−0.17	1.05	−0.18
12	−2.46	−2.45	0.00	0.99	0.00
13	−1.85	−1.92	−0.29	1.04	−0.30
14	−2.59	−3.01	0.00	1.16	0.00
15	−7.87	−7.71	−7.55	0.98	0.00
16	−4.12	−4.29	−0.59	1.04	−0.61
17	−3.56	−3.77	−0.35	1.06	−0.37
18	−4.14	−4.46	−0.34	1.08	−0.37
19	−5.16	−5.65	−0.20	1.10	−0.22
20	−3.55	−3.74	−0.62	1.05	−0.65
21	−3.97	−4.25	−0.75	1.07	−0.80
22	−4.15	−4.48	−0.77	1.08	−0.83
23	−2.08	−2.30	−0.10	1.11	−0.11
24	−1.90	−2.17	0.00	1.14	0.00
25	−1.93	−1.84	−0.30	0.95	−0.29
26	−2.58	−2.79	−0.24	1.08	−0.26
27	−3.47	−3.53	−0.48	1.02	−0.49
28	−3.83	−3.92	−0.48	1.03	−0.49
29	−4.47	−4.72	−0.26	1.06	−0.27
30	−2.10	−2.24	−0.24	1.06	−0.26
31	−1.87	−2.02	−0.06	1.08	−0.06
32	−2.01	−1.92	−0.30	0.96	−0.29
33	−2.01	−2.15	0.00	1.07	0.00
34	−2.41	−2.74	0.00	1.14	0.00
35	−1.65	−1.73	−0.14	1.05	−0.15
36	−1.98	−2.06	−0.37	1.04	−0.39
37	−1.80	−1.79	−0.32	1.00	−0.32
38	−2.06	−2.07	−0.24	1.00	−0.24
39	−2.25	−2.30	−0.43	1.03	−0.44
40	−2.15	−2.11	−0.37	0.98	−0.36
41	−2.23	−2.24	−0.25	1.00	−0.25
42	−2.41	−2.40	−0.44	0.99	−0.44
43	−2.37	−2.37	−0.24	1.00	−0.24
44	−2.55	−2.53	−0.42	0.99	−0.42
Mean	–	–	−0.24	–	−0.25

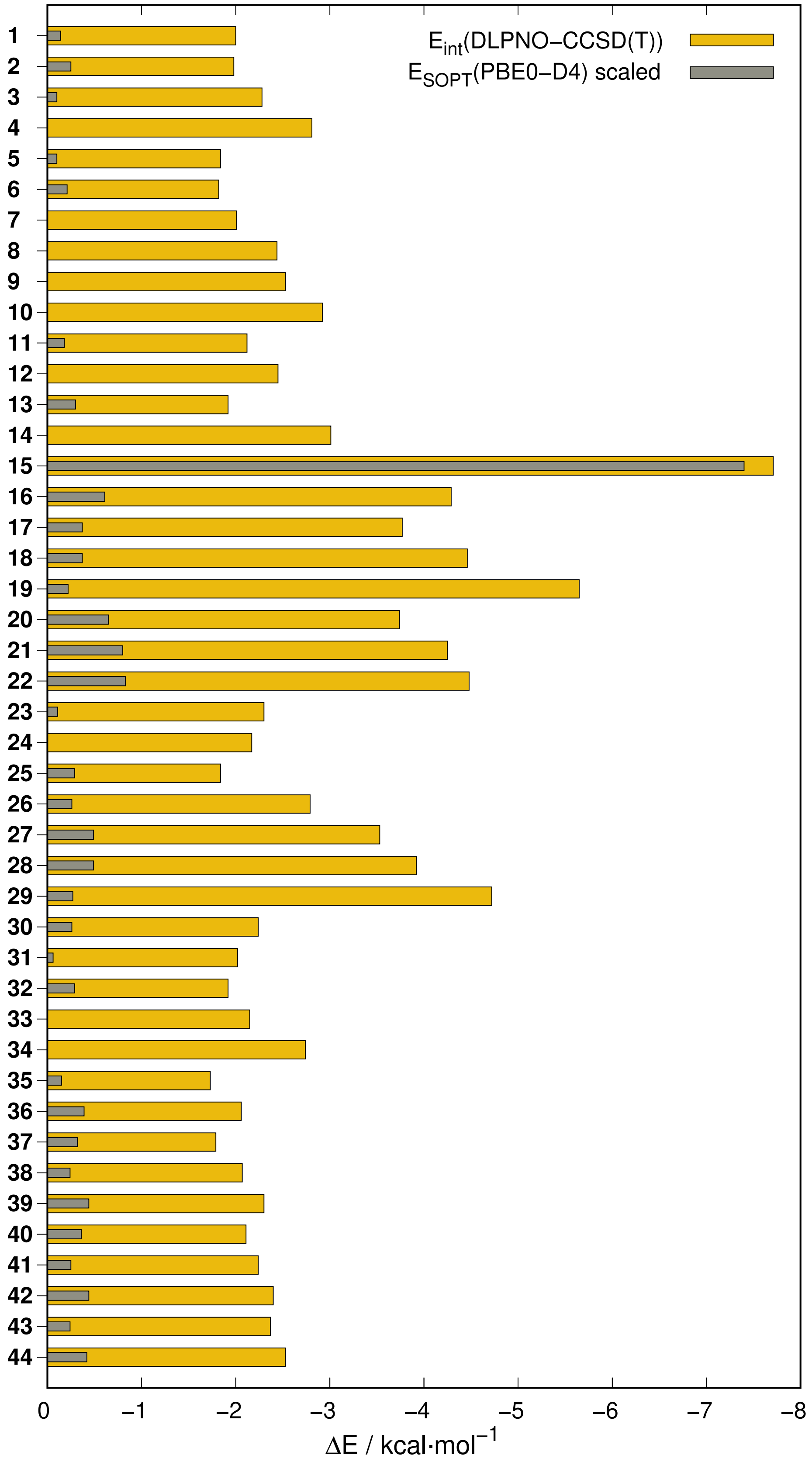

Figure 2.1: Scaled NBO SOPT hydrogen bonding estimate at the PBE0-D4/def2-QZVPP level and the interaction energy at DLPNO-CCSD(T)/def2-QZVPP level for all dimer systems.

2.8 Local Energy Decomposition for the Dimer Systems

Table S13: Local Energy Decomposition analysis contributions to the DLPNO-CCSD(T) interaction energy of all dimer systems in kcal·mol^{-1}. The last column shows the ratio between the dispersion contribution and the total interaction energy.

#	$E_{0,HF-elprep}$	$E_{0,elstat}$	$E_{0,exch}$	$E_{orb-relax}$	E_{disp}	$E_{non-disp}$	E_{int}	E_{disp}/E_{int}
1	14.453	−9.878	−3.638	−0.732	−2.459	0.250	−2.004	1.227
2	12.730	−8.853	−2.430	−0.838	−2.742	0.150	−1.983	1.383
3	12.540	−8.996	−2.310	−0.707	−3.118	0.313	−2.279	1.368
4	14.532	−9.541	−2.917	−0.648	−4.068	−0.167	−2.808	1.449
5	13.951	−9.172	−3.387	−0.531	−2.886	0.181	−1.843	1.566
6	13.401	−8.948	−2.639	−0.647	−2.991	0.005	−1.819	1.644
7	12.542	−8.577	−2.460	−0.589	−3.170	0.242	−2.012	1.576
8	14.914	−10.220	−2.919	−0.735	−3.574	0.097	−2.438	1.466
9	14.843	−10.221	−2.751	−0.748	−3.791	0.138	−2.530	1.498
10	18.610	−12.866	−3.467	−1.144	−4.357	0.302	−2.923	1.491
11	14.938	−10.905	−3.675	−0.745	−2.278	0.543	−2.123	1.073
12	15.783	−11.467	−3.419	−1.351	−2.925	0.933	−2.446	1.196
13	12.057	−8.621	−2.368	−0.872	−2.487	0.368	−1.922	1.294
14	15.806	−11.356	−3.109	−1.112	−3.985	0.742	−3.014	1.322
15	43.105	−33.743	−8.247	−6.279	−3.719	1.173	−7.711	0.482
16	17.672	−13.661	−3.324	−1.695	−3.991	0.707	−4.292	0.930
17	19.058	−13.992	−3.597	−1.724	−4.163	0.646	−3.771	1.104
18	20.731	−15.477	−3.717	−2.205	−4.670	0.877	−4.461	1.047
19	28.696	−21.617	−5.074	−3.267	−5.898	1.507	−5.652	1.044
20	19.955	−14.650	−3.847	−1.847	−4.013	0.664	−3.739	1.073
21	21.023	−15.501	−3.737	−2.300	−4.439	0.703	−4.251	1.044
22	22.487	−16.398	−4.004	−2.518	−4.776	0.732	−4.476	1.067
23	10.988	−8.318	−2.088	−0.444	−2.825	0.383	−2.303	1.227
24	13.075	−9.365	−2.425	−0.580	−2.938	0.062	−2.172	1.353
25	13.206	−9.133	−2.540	−0.703	−2.776	0.103	−1.843	1.506
26	17.492	−11.838	−3.375	−0.939	−4.586	0.458	−2.788	1.645
27	20.204	−14.288	−3.803	−1.476	−4.493	0.329	−3.527	1.274
28	20.649	−14.741	−3.737	−1.682	−4.776	0.366	−3.922	1.218
29	26.965	−19.276	−4.844	−2.314	−6.029	0.776	−4.722	1.277
30	12.558	−8.941	−2.378	−0.583	−3.258	0.364	−2.238	1.456
31	13.558	−9.347	−2.575	−0.583	−3.199	0.124	−2.022	1.582
32	14.011	−9.745	−2.462	−0.757	−2.972	0.007	−1.918	1.550
33	13.981	−9.536	−2.526	−0.689	−3.474	0.093	−2.151	1.615
34	19.108	−11.553	−3.229	−2.658	−4.130	−0.279	−2.742	1.506
35	13.004	−9.515	−2.988	−0.521	−1.933	0.224	−1.729	1.118
36	9.504	−7.057	−1.738	−0.647	−2.387	0.265	−2.061	1.158
37	9.872	−7.276	−2.000	−0.559	−2.060	0.227	−1.795	1.148
38	14.331	−9.823	−3.644	−0.597	−2.592	0.250	−2.074	1.250
39	12.469	−8.970	−2.236	−0.704	−3.066	0.201	−2.305	1.330
40	12.597	−9.004	−2.517	−0.628	−2.772	0.212	−2.111	1.313
41	11.425	−8.387	−2.024	−0.642	−2.752	0.139	−2.241	1.228
42	13.599	−9.699	−2.372	−0.756	−3.267	0.094	−2.401	1.361
43	12.161	−8.601	−2.268	−0.680	−3.089	0.102	−2.375	1.301
44	14.753	−10.116	−2.707	−0.819	−3.638	−0.002	−2.528	1.439

2.9 DFT-D4 Dispersion Energy vs LED Dispersion Energy (Dimer Systems)

Table S14: B3LYP-D4 dispersion corrections for the dimer and both fragments of the systems **1-44** as well as the resulting D4 energy differences, unscaled and scaled by 1.25 respectively. The last column shows the LED dispersion contribution, all values in kcal·mol^{-1}.

#	D4(Dimer)	D4(Frag1)	D4(Frag2)	ΔE_{disp}(B3LYP-D4)	$\Delta E_{scal,disp}$(B3LYP-D4)	ΔE_{disp}(LED)
1	−6.733	−1.366	−3.438	−1.929	−2.411	−2.459
2	−11.766	−6.295	−3.436	−2.034	−2.543	−2.742
3	−15.668	−9.917	−3.437	−2.314	−2.892	−3.118
4	−14.359	−7.721	−3.432	−3.206	−4.008	−4.068
5	−8.234	−2.353	−3.428	−2.453	−3.066	−2.886
6	−14.055	−8.250	−3.428	−2.377	−2.971	−2.991
7	−18.320	−12.360	−3.429	−2.532	−3.165	−3.170
8	−15.916	−9.611	−3.430	−2.875	−3.593	−3.574
9	−16.404	−9.828	−3.428	−3.148	−3.935	−3.791
10	−17.395	−10.416	−3.424	−3.555	−4.444	−4.357
11	−6.063	−0.901	−3.437	−1.726	−2.157	−2.278
12	−6.398	−0.901	−3.428	−2.070	−2.587	−2.925
13	−10.686	−5.330	−3.437	−1.919	−2.399	−2.487
14	−11.969	−5.333	−3.434	−3.203	−4.003	−3.985
15	−7.424	−1.664	−3.433	−2.326	−2.908	−3.720
16	−15.639	−8.867	−3.439	−3.333	−4.166	−3.991
17	−18.418	−11.464	−3.442	−3.512	−4.389	−4.163
18	−18.866	−11.515	−3.445	−3.907	−4.883	−4.670
19	−19.858	−11.636	−3.445	−4.778	−5.972	−5.898
20	−13.556	−6.945	−3.440	−3.172	−3.965	−4.013
21	−14.288	−7.375	−3.442	−3.471	−4.339	−4.439
22	−15.197	−7.999	−3.443	−3.755	−4.693	−4.776
23	−12.187	−6.574	−3.431	−2.182	−2.727	−2.825
24	−9.330	−3.680	−3.435	−2.215	−2.769	−2.938
25	−12.821	−7.155	−3.431	−2.235	−2.793	−2.776
26	−14.304	−7.159	−3.429	−3.716	−4.646	−4.586
27	−21.032	−13.864	−3.436	−3.731	−4.664	−4.493
28	−21.231	−13.704	−3.437	−4.089	−5.112	−4.776
29	−22.141	−13.619	−3.436	−5.086	−6.358	−6.029
30	−14.892	−8.831	−3.425	−2.636	−3.295	−3.258
31	−11.209	−5.227	−3.429	−2.553	−3.191	−3.199
32	−13.455	−7.564	−3.430	−2.460	−3.075	−2.972
33	−11.873	−5.578	−3.427	−2.868	−3.585	−3.474
34	−12.936	−6.225	−3.423	−3.288	−4.110	−4.010
35	−6.017	−1.054	−3.434	−1.530	−1.912	−1.933
36	−16.490	−11.202	−3.431	−1.857	−2.322	−2.387
37	−7.314	−2.192	−3.431	−1.691	−2.114	−2.060
38	−7.428	−1.887	−3.432	−2.109	−2.637	−2.592
39	−18.972	−13.173	−3.428	−2.371	−2.964	−3.066
40	−9.054	−3.387	−3.428	−2.238	−2.798	−2.772
41	−7.916	−2.083	−3.432	−2.401	−3.002	−2.752
42	−19.838	−13.681	−3.428	−2.730	−3.412	−3.267
43	−8.613	−2.457	−3.430	−2.725	−3.407	−3.089
44	−21.029	−14.520	−3.426	−3.084	−3.855	−3.638

2.10 DFT-D4 Corrected DFAs vs DLPNO-CCSD(T) Scans

Table S15: Interaction energy for system **13** for various DFAs and DLPNO-CCSD(T) at O···N2 distances between 1.8 and 6.0 Å, all energies in kcal·mol^{-1}.

Dist.	PBE-D4	BLYP-D4	PBE0-D4	B3LYP-D4	DLPNO-CCSD(T)
1.8	81.887	86.597	83.504	87.183	85.640
1.9	58.357	61.881	59.387	62.161	60.651
2.0	40.896	43.492	41.479	43.533	42.103
2.1	28.086	29.969	28.353	29.839	28.495
2.2	18.779	20.121	18.844	19.886	18.736
2.3	12.090	13.027	12.038	12.738	11.733
2.4	7.339	7.986	7.233	7.677	6.795
2.5	4.016	4.461	3.896	4.156	3.475
2.6	1.735	2.051	1.626	1.760	1.250
2.7	0.208	0.450	0.122	0.179	−0.188
2.8	−0.777	−0.571	−0.834	−0.821	−1.078
2.9	−1.378	−1.178	−1.407	−1.409	−1.578
3.0	−1.712	−1.496	−1.717	−1.712	−1.829
3.1	−1.859	−1.621	−1.844	−1.823	−1.921
3.2	−1.881	−1.618	−1.851	−1.808	−1.889
3.3	−1.824	−1.530	−1.784	−1.710	−1.784
3.4	−1.716	−1.397	−1.669	−1.568	−1.644
3.5	−1.577	−1.243	−1.527	−1.405	−1.483
3.6	−1.426	−1.079	−1.375	−1.232	−1.315
3.7	−1.271	−0.916	−1.222	−1.062	−1.151
3.8	−1.118	−0.766	−1.071	−0.903	−0.997
3.9	−0.971	−0.628	−0.928	−0.756	−0.857
4.0	−0.835	−0.502	−0.797	−0.622	−0.725
4.1	−0.710	−0.391	−0.676	−0.504	−0.609
4.2	−0.595	−0.297	−0.566	−0.401	−0.507
4.3	−0.492	−0.215	−0.468	−0.311	−0.421
4.4	−0.400	−0.144	−0.380	−0.233	−0.343
4.5	−0.319	−0.084	−0.303	−0.166	−0.274
4.6	−0.247	−0.034	−0.235	−0.109	−0.217
4.7	−0.184	0.007	−0.176	−0.062	−0.163
4.8	−0.129	0.043	−0.124	−0.020	−0.118
4.9	−0.082	0.073	−0.079	0.015	−0.079
5.0	−0.041	0.098	−0.041	0.044	−0.046
5.1	−0.005	0.117	−0.007	0.068	−0.015
5.2	0.025	0.134	0.021	0.088	0.008
5.3	0.051	0.148	0.046	0.106	0.027
5.4	0.073	0.160	0.067	0.121	0.045
5.5	0.092	0.170	0.085	0.134	0.063
5.6	0.108	0.178	0.100	0.144	0.077
5.7	0.121	0.184	0.112	0.152	0.086
5.8	0.132	0.190	0.123	0.160	0.093
5.9	0.141	0.195	0.131	0.166	0.099
6.0	0.149	0.198	0.139	0.171	0.104

Table S16: Interaction energy for system **5** for various DFAs and DLPNO-CCSD(T) at P $\cdots$ N2 distances between 1.8 and 6.0 Å, all energies in kcal·mol^{-1}.

Dist.	PBE-D4	BLYP-D4	PBE0-D4	B3LYP-D4	DLPNO-CCSD(T)
1.8	219.477	232.272	228.326	237.918	237.677
1.9	169.953	180.711	176.777	185.031	183.296
2.0	129.325	138.211	134.280	141.280	138.689
2.1	96.844	104.114	100.286	106.156	103.127
2.2	71.365	77.227	73.669	78.510	75.459
2.3	51.712	56.365	53.201	57.119	54.164
2.4	36.769	40.390	37.697	40.800	38.078
2.5	25.522	28.228	26.083	28.449	26.127
2.6	17.130	19.006	17.469	19.161	17.372
2.7	11.056	12.348	11.255	12.443	11.068
2.8	6.706	7.571	6.823	7.625	6.587
2.9	3.629	4.184	3.706	4.213	3.478
3.0	1.493	1.826	1.556	1.841	1.346
3.1	0.045	0.232	0.107	0.236	−0.065
3.2	−0.904	−0.804	−0.836	−0.809	−0.949
3.3	−1.495	−1.439	−1.419	−1.454	−1.462
3.4	−1.833	−1.788	−1.749	−1.813	−1.735
3.5	−1.994	−1.937	−1.905	−1.973	−1.842
3.6	−2.035	−1.953	−1.944	−1.999	−1.841
3.7	−1.996	−1.883	−1.904	−1.938	−1.770
3.8	−1.904	−1.761	−1.815	−1.823	−1.662
3.9	−1.783	−1.610	−1.697	−1.678	−1.530
4.0	−1.645	−1.448	−1.565	−1.520	−1.388
4.1	−1.500	−1.287	−1.426	−1.360	−1.245
4.2	−1.356	−1.133	−1.288	−1.206	−1.107
4.3	−1.217	−0.991	−1.155	−1.061	−0.979
4.4	−1.086	−0.861	−1.031	−0.928	−0.862
4.5	−0.964	−0.746	−0.915	−0.809	−0.754
4.6	−0.851	−0.645	−0.808	−0.703	−0.659
4.7	−0.749	−0.557	−0.711	−0.610	−0.575
4.8	−0.657	−0.480	−0.624	−0.528	−0.501
4.9	−0.575	−0.413	−0.546	−0.457	−0.433
5.0	−0.501	−0.357	−0.476	−0.395	−0.374
5.1	−0.435	−0.309	−0.414	−0.342	−0.322
5.2	−0.377	−0.267	−0.359	−0.296	−0.279
5.3	−0.325	−0.231	−0.311	−0.256	−0.239
5.4	−0.280	−0.200	−0.268	−0.221	−0.206
5.5	−0.241	−0.173	−0.230	−0.192	−0.176
5.6	−0.206	−0.150	−0.197	−0.166	−0.149
5.7	−0.175	−0.130	−0.169	−0.143	−0.125
5.8	−0.148	−0.112	−0.143	−0.123	−0.102
5.9	−0.125	−0.097	−0.121	−0.106	−0.086
6.0	−0.104	−0.083	−0.102	−0.090	−0.071

2.11 DFT-D4 Dispersion Energy vs LED Dispersion Energy (Scans)

Table S17: D4 dispersion energy differences for system **13** at O···N2 distances between 1.8 and 6.0 Å, all energies in kcal·mol^{-1}. The last column shows the LED dispersion energy difference for comparison.

Dist.	ΔE_{disp}(PBE-D4)	ΔE_{disp}(BLYP-D4)	ΔE_{disp}(PBE0-D4)	ΔE_{disp}(B3LYP-D4)	ΔE_{disp}(LED)
1.8	−3.041	−6.202	−2.681	−4.650	−17.645
1.9	−2.961	−6.015	−2.616	−4.520	−15.286
2.0	−2.850	−5.757	−2.524	−4.340	−13.038
2.1	−2.716	−5.449	−2.415	−4.126	−11.227
2.2	−2.576	−5.124	−2.299	−3.899	−9.590
2.3	−2.434	−4.795	−2.182	−3.671	−8.380
2.4	−2.293	−4.467	−2.065	−3.443	−7.172
2.5	−2.153	−4.142	−1.950	−3.217	−6.173
2.6	−2.015	−3.822	−1.835	−2.993	−5.337
2.7	−1.879	−3.509	−1.722	−2.774	−4.585
2.8	−1.746	−3.205	−1.611	−2.559	−3.965
2.9	−1.616	−2.912	−1.501	−2.349	−3.432
3.0	−1.490	−2.632	−1.394	−2.147	−2.967
3.1	−1.368	−2.367	−1.290	−1.953	−2.567
3.2	−1.252	−2.118	−1.189	−1.769	−2.212
3.3	−1.141	−1.887	−1.092	−1.595	−1.932
3.4	−1.036	−1.675	−0.999	−1.433	−1.703
3.5	−0.938	−1.481	−0.911	−1.282	−1.471
3.6	−0.846	−1.305	−0.829	−1.143	−1.277
3.7	−0.762	−1.147	−0.751	−1.016	−1.128
3.8	−0.684	−1.006	−0.679	−0.901	−0.972
3.9	−0.613	−0.880	−0.612	−0.798	−0.854
4.0	−0.548	−0.769	−0.551	−0.705	−0.749
4.1	−0.489	−0.672	−0.495	−0.622	−0.662
4.2	−0.436	−0.586	−0.444	−0.547	−0.590
4.3	−0.389	−0.510	−0.397	−0.482	−0.536
4.4	−0.346	−0.444	−0.356	−0.424	−0.476
4.5	−0.308	−0.386	−0.318	−0.372	−0.430
4.6	−0.274	−0.335	−0.284	−0.327	−0.391
4.7	−0.243	−0.290	−0.253	−0.286	−0.346
4.8	−0.216	−0.251	−0.226	−0.251	−0.308
4.9	−0.191	−0.217	−0.201	−0.220	−0.275
5.0	−0.170	−0.187	−0.179	−0.192	−0.246
5.1	−0.150	−0.160	−0.160	−0.167	−0.218
5.2	−0.133	−0.136	−0.142	−0.146	−0.196
5.3	−0.117	−0.116	−0.126	−0.126	−0.177
5.4	−0.104	−0.097	−0.112	−0.109	−0.159
5.5	−0.091	−0.081	−0.100	−0.094	−0.144
5.6	−0.080	−0.066	−0.088	−0.080	−0.130
5.7	−0.070	−0.054	−0.078	−0.068	−0.118
5.8	−0.061	−0.042	−0.069	−0.058	−0.107
5.9	−0.053	−0.032	−0.060	−0.048	−0.097
6.0	−0.045	−0.023	−0.053	−0.039	−0.088

Table S18: D4 dispersion energy differences for system **5** at P $\cdots$ N2 distances between 1.8 and 6.0 Å, all energies in kcal·mol^{-1}. The last column shows the LED dispersion energy difference for comparison.

Dist.	ΔE_{disp}(PBE-D4)	ΔE_{disp}(BLYP-D4)	ΔE_{disp}(PBE0-D4)	ΔE_{disp}(B3LYP-D4)	ΔE_{disp}(LED)
1.8	–3.430	–7.407	–2.934	–5.469	–32.903
1.9	–3.382	–7.273	–2.902	–5.386	–28.201
2.0	–3.322	–7.113	–2.858	–5.284	–24.303
2.1	–3.210	–6.839	–2.772	–5.098	–21.071
2.2	–3.096	–6.560	–2.683	–4.909	–18.244
2.3	–2.983	–6.285	–2.595	–4.721	–15.834
2.4	–2.879	–6.031	–2.514	–4.548	–13.692
2.5	–2.818	–5.875	–2.469	–4.445	–11.867
2.6	–2.813	–5.843	–2.471	–4.434	–10.227
2.7	–2.721	–5.614	–2.401	–4.281	–8.921
2.8	–2.597	–5.311	–2.303	–4.073	–7.823
2.9	–2.463	–4.985	–2.198	–3.849	–6.789
3.0	–2.326	–4.651	–2.088	–3.619	–5.838
3.1	–2.186	–4.313	–1.977	–3.385	–5.145
3.2	–2.046	–3.976	–1.865	–3.149	–4.460
3.3	–1.906	–3.643	–1.752	–2.914	–3.868
3.4	–1.768	–3.317	–1.639	–2.682	–3.327
3.5	–1.631	–3.004	–1.526	–2.454	–2.889
3.6	–1.499	–2.705	–1.415	–2.235	–2.509
3.7	–1.371	–2.424	–1.306	–2.025	–2.196
3.8	–1.250	–2.163	–1.201	–1.826	–1.906
3.9	–1.134	–1.923	–1.100	–1.640	–1.676
4.0	–1.027	–1.705	–1.004	–1.468	–1.431
4.1	–0.927	–1.508	–0.913	–1.310	–1.254
4.2	–0.834	–1.331	–0.828	–1.167	–1.104
4.3	–0.750	–1.175	–0.749	–1.037	–0.972
4.4	–0.673	–1.036	–0.676	–0.921	–0.838
4.5	–0.604	–0.913	–0.609	–0.817	–0.769
4.6	–0.541	–0.806	–0.549	–0.725	–0.680
4.7	–0.485	–0.711	–0.494	–0.643	–0.595
4.8	–0.435	–0.629	–0.444	–0.571	–0.536
4.9	–0.390	–0.556	–0.399	–0.508	–0.469
5.0	–0.350	–0.493	–0.359	–0.452	–0.418
5.1	–0.315	–0.438	–0.323	–0.402	–0.374
5.2	–0.283	–0.389	–0.291	–0.359	–0.332
5.3	–0.255	–0.346	–0.262	–0.320	–0.307
5.4	–0.229	–0.309	–0.237	–0.286	–0.276
5.5	–0.207	–0.276	–0.214	–0.256	–0.247
5.6	–0.187	–0.247	–0.193	–0.230	–0.223
5.7	–0.169	–0.221	–0.175	–0.206	–0.200
5.8	–0.153	–0.199	–0.158	–0.186	–0.180
5.9	–0.139	–0.179	–0.144	–0.167	–0.162
6.0	–0.126	–0.161	–0.131	–0.151	–0.147

2.12 Gas Phase Conformational Study

Table S19: Relative free energies of gas phase conformers for **A** and **B**, final rerank at the DLPNO-CCSD(T)/CBS//SCS-MP2/def2-TZVPP level of theory, duplicate conformers (generated by reoptimization) were sorted out, all energies in kcal·mol^{-1}.

System **A**			System **B**		
Conformer #	Motif present?	ΔG	Conformer #	Motif present?	ΔG
1	No	0.000	1	No	0.000
2	Yes	0.036	2	No	0.284
3	Yes	0.093	3	No	0.414
4	Yes	0.123	4	No	0.619
5	Yes	0.136	5	No	0.787
6	Yes	0.243	6	No	1.201
7	Yes	0.288	7	Yes	1.295
8	No	0.370	8	Yes	1.300
9	No	0.420	9	No	1.311
10	No	0.553	10	No	1.326
11	No	0.603	11	No	1.327
12	No	0.645	12	No	1.653
13	No	0.722	13	Yes	2.074
14	Yes	0.724			
15	Yes	0.787			
16	Yes	0.813			
17	No	0.873			
18	No	0.874			
19	No	0.966			
X-ray	Yes	0.189	X-ray	Yes	3.128

2.13 LED Analysis of Constrained X-ray Structure Fragments

Table S20: LED analysis on intermolecular model systems for X-ray structures **A** and **B** at DLPNO-CCSD(T)/def2-QZVPP//SCS-MP2/def2-QZVPP, the descriptor (X,Y) indicates if the fragments (PCH and azide moiety) were saturated with hydrogen or methyl groups. **B(Me-rep.)** is a methane dimer obtained by constrained optimization of hydrogen-saturated methyl groups from system **B(Me,Me)** and gives an estimate of the methyl group repulsion, all values in kcal·mol^{-1}.

**System**	**A(H,Me)**	**B(Me,Me)**	**B(Me-rep.)**	B(Me,Me)-B(Me-rep.)	**B(H,Me)**
$E_{0,HF-elprep}$	20.941	28.754	12.040	16.714	20.999
$E_{0,elstat}$	−11.969	−16.398	−5.901	−10.497	−13.008
$E_{0,exch}$	−4.269	−5.053	−2.550	−2.503	−3.610
$E_{orb-relax}$	−1.092	−1.897	−0.391	−1.507	−1.312
E_{disp}	−5.036	−5.467	−1.929	−3.538	−4.239
$E_{non-disp}$	−0.363	−0.286	−0.292	0.006	−0.016
E_{int}	−1.787	−0.347	0.977	−1.325	−1.186

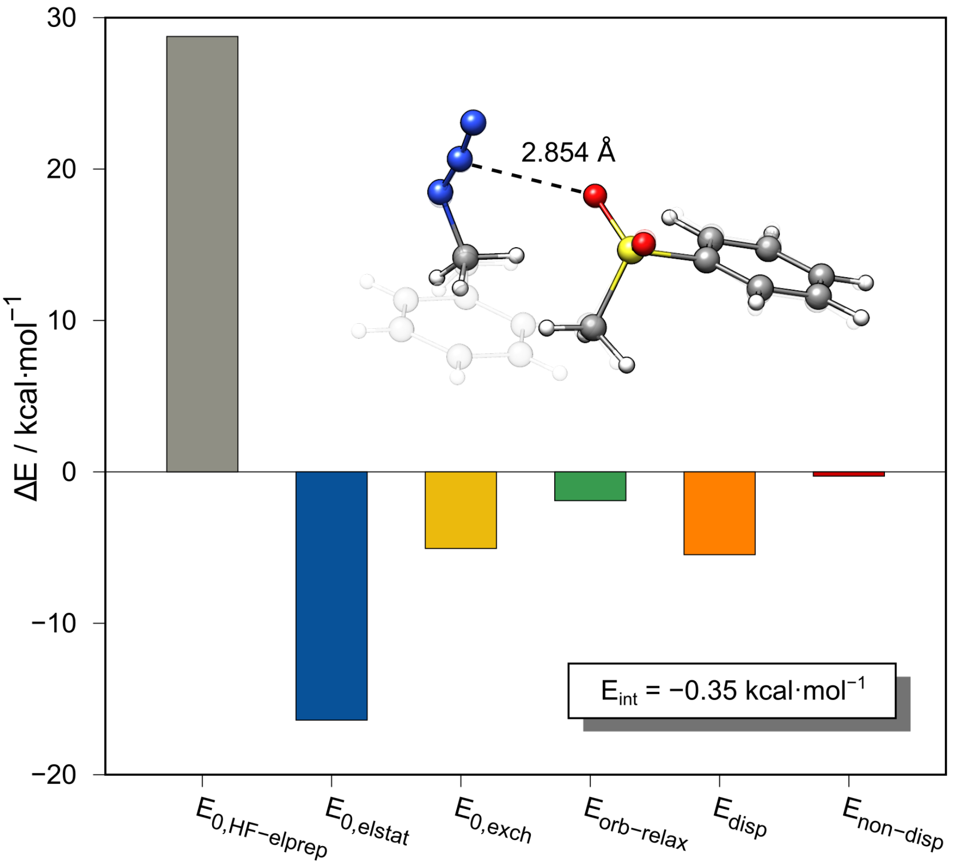

Figure 2.2: LED analysis on intermolecular model system **B(Me,Me)** at the DLPNO-CCSD(T)/def2-QZVPP//SCS-MP2/def2-QZVPP, fragments were saturated with methyl groups. The corresponding X-ray structure is depicted transparently.

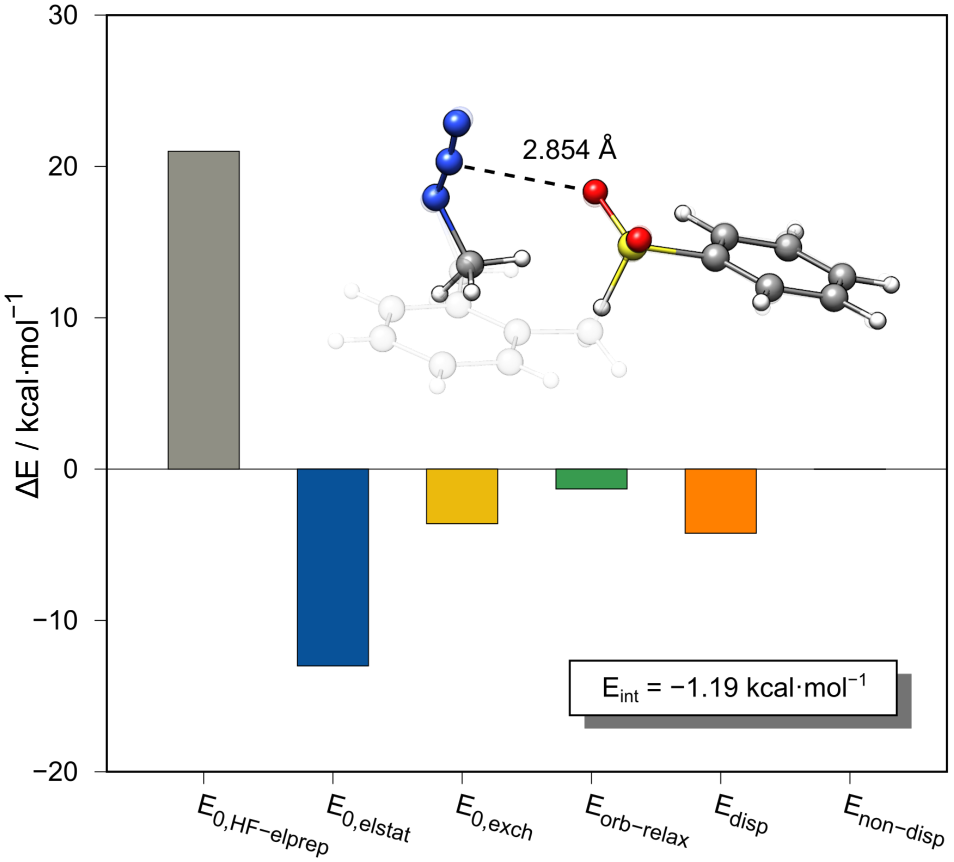

Figure 2.3: LED analysis on intermolecular model system **B(H,Me)** at the DLPNO-CCSD(T)/def2-QZVPP//SCS-MP2/def2-QZVPP, fragments were saturated with methyl groups (azido fragment) and hydrogen. The corresponding X-ray structure is depicted transparently.

3 References

[1] G. M. Sheldrick, *Acta Cryst. C* **2015**, *71*, 3–8.

[2] E. F. Pettersen, T. D. Goddard, C. C. Huang, G. S. Couch, D. M. Greenblatt, E. C. Meng, T. E. Ferrin, *J. Comput. Chem.* **2004**, *25*, 1605–1612.

[3] F. Neese, *Wiley Interdiscip. Rev. Comput. Mol. Sci.* **2012**, *2*, 73–78.

[4] H. J. Werner, P. J. Knowles, G. Knizia, F. R. Manby, M. Schütz, *Wiley Interdiscip. Rev. Comput. Mol. Sci.* **2012**, *2*, 242–253.

[5] F. Furche, R. Ahlrichs, C. Hättig, W. Klopper, M. Sierka, F. Weigend, *Wiley Interdiscip. Rev. Comput. Mol. Sci.* **2014**, *4*, 91–100.

[6] TURBOMOLE V7.0 2015, a development of University of Karlsruhe and Forschungszentrum Karlsruhe GmbH, 1989-2007, TURBOMOLE GmbH, since 2007; available from *http://www.turbomole.com.*

[7] C. Bannwarth, S. Ehlert, S. Grimme, *J. Chem. Theory Comput.* **2019**, *15*, 1652–1671.

[8] T. Risthaus, M. Steinmetz, S. Grimme, *J. Comput. Chem.* **2014**, *35*, 1509–1516.

[9] C. Riplinger, B. Sandhoefer, A. Hansen, F. Neese, *J. Chem. Phys.* **2013**, *139*, 134101.

[10] C. Riplinger, P. Pinski, U. Becker, E. F. Valeev, F. Neese, *J. Chem. Phys.* **2016**, *144*.

[11] F. Pavošević, C. Peng, P. Pinski, C. Riplinger, F. Neese, E. F. Valeev, *J. Chem. Phys.* **2017**, *146*.

[12] D. Andrae, U. Häußermann, M. Dolg, H. Stoll, H. Preuß, *Theor. Chim. Acta* **1990**, *77*, 123–141.

[13] P. Pracht, F. Bohle, S. Grimme, *Phys. Chem. Chem. Phys.* **2020**.

[14] S. Grimme, C. Bannwarth, S. Dohm, A. Hansen, J. Pisarek, P. Pracht, J. Seibert, F. Neese, *Angew. Chem. - Int. Ed.* **2017**, *56*, 14763–14769.

[15] F. Bohle, ENSO, Mulliken Center for Theoretical Chemistry, Universität Bonn, **2020**.

[16] S. Grimme, *Chem. Eur. J.* **2012**, *18*, 9955–9964.

[17] F. Neese, E. F. Valeev, *J. Chem. Theory Comput.* **2011**, *7*, 33–43.

[18] A. Karton, J. M. Martin, *J. Chem. Phys.* **2012**, *136*.

[19] A. Altun, F. Neese, G. Bistoni, *J. Chem. Theory Comput.* **2019**, *15*, 215–228.

[20] Q. Lu, F. Neese, G. Bistoni, *Phys. Chem. Chem. Phys.* **2019**, *21*, 11569–11577.
